# Research on Synthesis, Structure, and Catalytic Performance of Tetranuclear Copper(I) Clusters Supported by 2-Mercaptobenz-zole-Type Ligands

**DOI:** 10.3390/molecules29174228

**Published:** 2024-09-06

**Authors:** Tingyu Zhu, Wangyuan Zhan, Weibin Fan, Xiaofeng Zhang

**Affiliations:** 1 State Key Laboratory of Structural Chemistry, Fujian Institute of Research on the Structure of Matter, University of Chinese Academy of Sciences, Fuzhou 350002, China; zhutingyu@fjirsm.ac.cn (T.Z.); zhanwangyuan@fjirsm.ac.cn (W.Z.); fanweibin@fjirsm.ac.cn (W.F.); 2 College of Chemistry and Materials Science, Fujian Normal University, Fuzhou 350007, China; 3 Fujian College, University of Chinese Academy of Sciences, Fuzhou 350002, China

**Keywords:** tetrahedral copper(I) clusters, regulation of clusters, catalytic reaction, AIBN, fluorescence materials

## Abstract

Tetrahedral copper(I) clusters [Cu_4_(MBIZ)_4_(PPh_3_)_2_] (**2**), [Cu_4_(MBOZ)_4_(PPh_3_)_4_] (**6**) (MBIZ = 2-mercaptobenzimidazole, MBOZ = 2-mercaptobenzoxazole) were prepared by regulation of the copper-thiolate clusters [Cu_6_(MBIZ)_6_] (**1**) and [Cu_8_(MBOZ)_8_I]^−^ (**5**) with PPh_3_. With the presence of iodide anion, the regulation provided the iodide-containing clusters [Cu^I^_4_(MBIZ)_3_(PPh_3_)_3_I] (**3**) and [Cu^I^_4_(MBOZ)_3_(PPh_3_)_3_I] (**7**). The cyclic voltammogram of **3** in MeCN (0.1 M ^n^Bu_4_NPF_6_, 298 K) at a scan rate of 100 mV s^−1^ shows two oxidation processes at E_p_^a^ = +0.11 and +0.45 V with return waves observed at E_p_^c^ = +0.25 V (vs. Fc^+^/Fc). Complex **3** has a higher capability to lose and gain electrons in the redox processes than complexes **2**, **4**, **4**′, **6,** and **7**. Its thermal stability was confirmed by thermogravimetric analysis. The catalytic performance of **3** was demonstrated by the catalytic transformation of iodobenzenes to benzonitriles using AIBN as the cyanide source. The nitrile products show potential applications in the preparation of 1,3,5-triazine compounds for organic fluorescence materials.

## 1. Introduction

Copper cluster compounds exhibit versatile properties and potential applications in the fields of catalysis [1,2,3,4,5,6], photoluminescent materials [7,8,9,10], sensing [11,12,13], biology and medicine [14,15,16,17], and electronics [18,19]. The tetranuclear copper(I) clusters have received great attention as a result of structural certainty for the convenience of study on the relationship between their configuration and performance. A number of clusters have been reported in the literature. They were mostly supported by small molecule ligands containing carbon [5,7,20], nitrogen [1,21,22], oxygen [23,24,25], halogen [6,8,9,10], phosphine [26,27,28], sulfur [2,3,4,29,30,31], and selenium [14,32,33,34] as coordination atoms. Among which, the [Cu^I^_4_] clusters, such as copper-halide [8,9,10] and copper-selenolate [34] clusters, were usually studied for the sensing applications on account of their good performance in photoluminescence; copper-acetate clusters were shown to be potential precursors for the chemical vapor deposition of copper onto substrate materials in the study of the deep submicron integrated circuits [24]. The copper-acetylide [5], copper-alkylamide [1], and copper-thiolate clusters [2,3,4,29,30] were reported to show chemical catalytic reforming or have biological catalysis in enzymes or cells.

**Figure 1:**
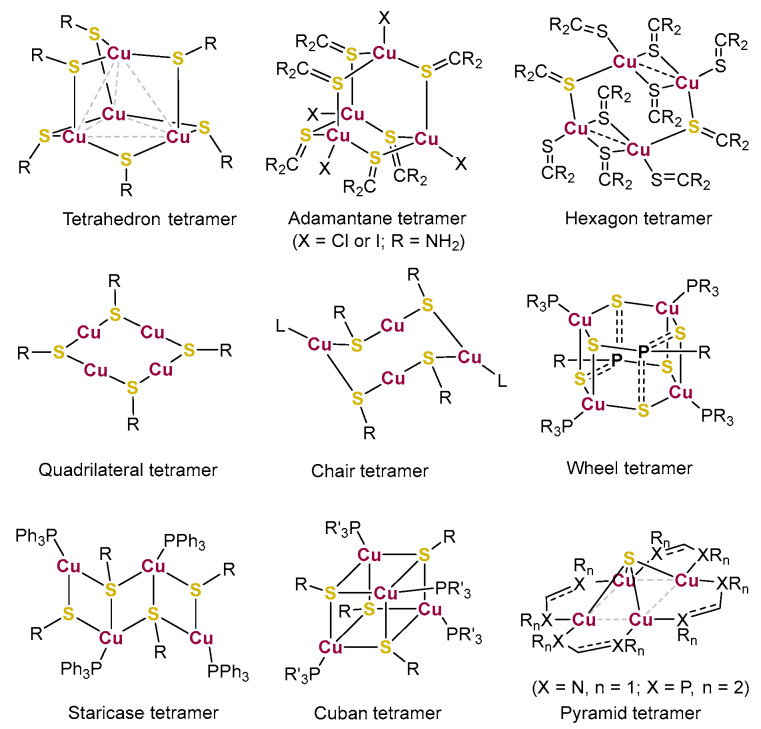
Various tetranuclear [Cu^I^_4_] clusters bridging by *μ*_x_-SR ligands (x = 2, 3, or 4).

Tetranuclear [Cu^I^_4_(SR)_x_] clusters show abundant structural diversity with various *μ*_x_-SR bridging ligands, which play important roles in the configuration and stability of the skeleton of the clusters (Figure 1). The tetrahedron-type tetramers were usually formed by self-assembling of four Cu^+^ ions and six *μ*_2_-SR thiolates (or three dithiolates), with the Cu···Cu distances ranging from 2.6 to 2.9 Å [35,36,37,38,39]. The coordination of six *μ*_2_-SR thione ligands (e.g., thiourea) to four Cu^+^ ions with the support of four halide ions (Cl^−^ or I^−^) results in the formation of the adamantane tetramers with a lengthening of Cu···Cu distances between 3.6 and 3.9 Å [40,41]. As electron-deficient ligands, thione ligands were also employed to produce hexagon tetramers [42,43,44,45,46]. Moreover, the *μ*_2_-SR bridging mode was applied in the construction of the quadrilateral-type [47,48,49,50,51] and the chair-type tetramers [52,53,54,55,56], in which the Cu^+^ ions were generally coordinated by electron-rich thiolates (e.g., BuS^−^, AdS^−^ (Ad = adamantyl)) side by side. The wheel-type tetramers were obtained by bridging four [Cu^I^(PR_3_)] units via tripodal [ArPS_3_]^2−^ ligands [57,58,59]. The combination of the *μ*_2_-SR and *μ*_3_-SR coordination modes gave rise to the staircase tetramers with the assistance of four triphenylphosphines as the terminal ligands [60,61,62,63,64]. In contrast, the bonding of four thiolates to four Cu^+^ ions in the *μ*_3_-SR mode only afforded the cubane-type tetramers [4,30]. A type of pyramid [Cu_4_S] tetramers were synthesized by binding of four Cu^+^ ions with a *μ*_4_-sulfur bridge under the support of four chelate ligands at the base [65,66,67,68]. Though there have been a significant number of copper(I) tetramers reported in the literature, the study of tetranuclear [Cu^I^] clusters with three or more types of ligands remains challenging and stimulating.

Aryl nitriles have wide applications not only in organic synthesis but also in materials sciences. The nitrile group can be easily transformed into a variety of functional groups, such as aldehydes, amines, amides, tetrazoles, triazoles, pyrimidines, triazine, etc. 1,3,5-triazine core was recognized as an electron acceptor that can make π-conjugated D-A molecules for application in organic electronic devices. The great significance has consistently stimulated the development of research methods for their preparation. 

Encouraged by these reasons, we start to study the synthesis of tetranuclear copper(I)-thiolate clusters with nitrogen-containing mercaptan ligands (2-mercaptobenzimidazole (MBIZ), 2-mercaptobenzoxazole (MBOZ), and 2-mercaptobenzothiazole (MBTZ). The clusters are studied to catalyze the transformation of aryl iodides to aryl nitriles. The latters are used to produce octupolar π-conjugated molecules for the study of fluorescent materials.

## 2. Results and Discussions

**Scheme 1:**
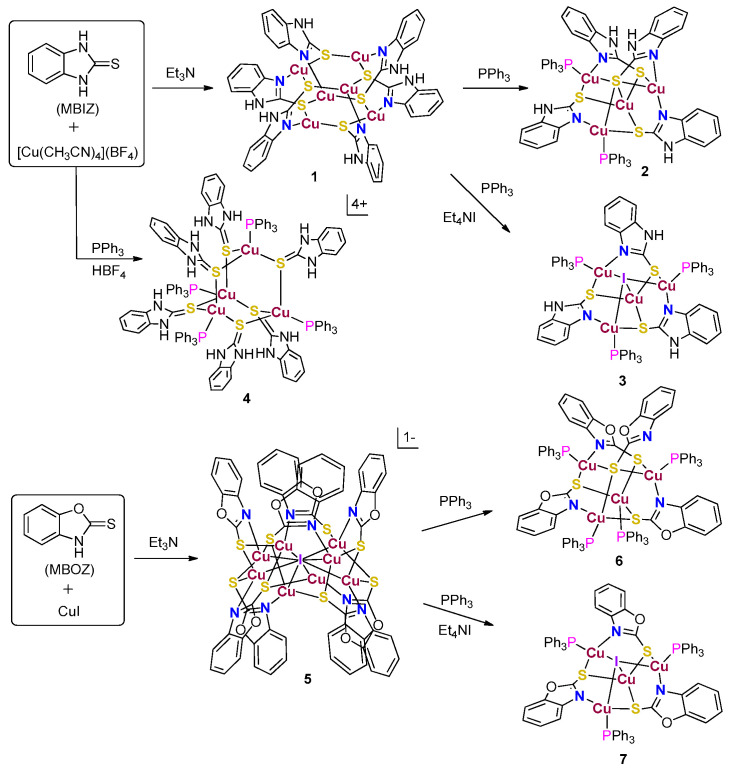
Synthetic routes for the preparation of Cu(I) clusters **1**–**7**.

**Figure 2:**
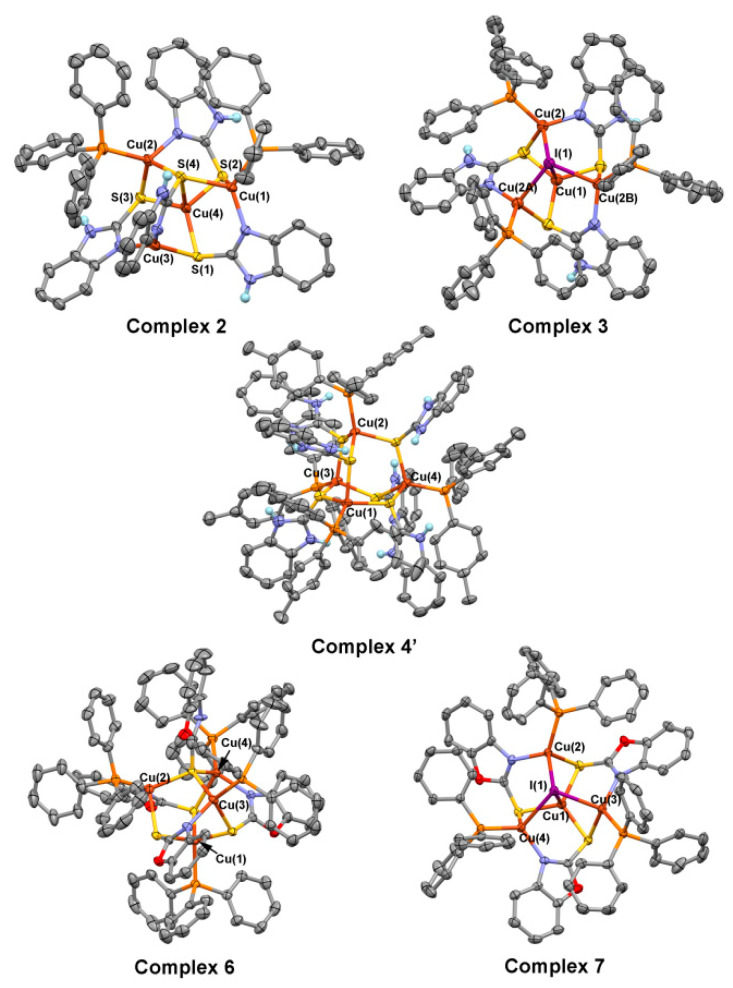
Crystal structures of complexes **2**, **3**, **4**′, **6**, and **7**.

We characterized all tetranuclear copper clusters by SC-XRD, XRD, and NMR (showed in Supplementary Materials). In air, the stirring of [Cu(CH_3_CN)_4_]BF_4_ with 2-mercaptobenzimidazole (MBIZ) in MeCN afforded a hexanuclear Cu(I) cluster [Cu^I^_6_(MBIZ)_6_]·THF (**1**) (Scheme 1) [69]. The compound was regulated by the addition of PPh_3_ to generate a tetranuclear Cu(I) cluster [Cu^I^_4_(MBIZ)_4_(PPh_3_)_2_]·3.5THF (**2**) with the Cu-S bond lengths and the Cu···Cu distances ranging from 2.26 to 2.85 Å and 2.68 to 4.26 Å, respectively. The regulation would lead to the formation of [Cu^I^_4_(MBIZ)_3_(PPh_3_)_3_I]·3THF·CH_3_CN (**3**) with the presence of Et_4_NI [31,70,71,72,73,74]. The iodide anion is located on the top of the cluster, with the Cu-I bond lengths being between 2.78 and 3.12 Å. The Cu-S bond lengths and the Cu···Cu distances are presented as 2.250(1)/2.402(1) Å and 2.862(1)/4.069(1) Å under the condition of P-3 symmetry. Meanwhile, the reaction of [Cu(CH_3_CN)_4_]BF_4_ with MBIZ and PPh_3_ (or P(*p*-CH_3_-Ph)_3_) in the presence of HBF_4_ in MeCN provided the adamantane-type [Cu^I^_4_(MBIZ)_6_(PPh_3_)_4_](BF_4_)_4_ (**4**) and [Cu^I^_4_(MBIZ)_6_(P(*p*-CH_3_-Ph)_3_)_4_] (BF_4_)_4_ (**4**′) clusters, in which any two Cu^I^ ions are bridged by one MBIZ-thione molecule with the Cu-S bond lengths and the Cu···Cu distances varying from 2.35 to 2.37 Å (**4**′ = 2.35 to 2.42 Å) and 4.05 to 4.06 Å (**4**′ = 3.96 to 4.18 Å). In comparison, the reaction of 2-mercaptobenzoxazole (MBOZ) with CuI produced an octo-nuclear cylinder-type Cu(I) cluster [Cu^I^_8_(MBOZ)_8_I](H_3_O)·CH_3_CN (**5**) [75]. An iodide anion is sited in the middle of this cylinder with the Cu-I bond lengths of 3.122(1) Å and 3.189(1) Å alternately. The cluster was regulated by PPh_3_ to form a tetranuclear Cu(I) cluster [Cu^I^_4_(MBOZ)_4_(PPh_3_)_4_] (**6**) [61,76]. In comparison to complex **2**, the top mercaptan ligand of complex **6** offers only the sulfur donor binding to the top three Cu^+^ ions, in accompany with an additional PPh_3_ binding to the bottom Cu^+^ ion. The change in the coordination mode leads to a lengthening of the S_top_···Cu_bottom_ distances from 2.847(2) to 3.695(1) Å. With the addition of Et_4_NI, the regulation produced the iodide-containing cluster [Cu^I^_4_(MBOZ)_3_(PPh_3_)_3_I]·CHCl_3_ (**7**) with a structure similar to complex **3**. The crystal structures of complexes **2**, **3**, **4**′, **6**, and **7** are shown in Figure 2. Unexpectedly, the reaction of the analogous ligand 2-mercaptobenzothiazole (MBTZ) with either [Cu(CH_3_CN)_4_]BF_4_ or CuI only afforded the dimer complex **8**. No corresponding cluster was obtained under the similar conditions.

**Figure 3:**
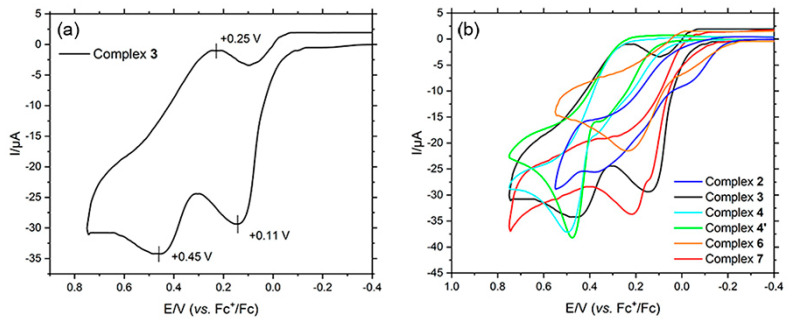
(**a**) Cyclic voltammogram of complex **3** in MeCN (0.1 M *^n^*Bu_4_NPF_6_) at 298 K; (**b**) Comparison of the oxidation processes of complexes **2**, **3**, **4**, **4**′, **6**, and **7**.

The cyclic voltammogram of complex **3** in MeCN (0.1 M *^n^*Bu_4_NPF_6_, 298 K) at a scan rate of 100 mV s^−1^ shows two oxidation processes at *E*_p_^a^ = +0.11 and +0.45 V with return waves observed at *E*_p_^c^ = +0.25 V (vs. Fc^+^/Fc) (Figure 3). It has inferior comparability with the fully irreversible process, with no return wave observed at *E*_p_^a^ = +0.15 and +0.21 V for complex **7** under the same experimental conditions. This suggests a degree of decreased kinetic activity for the [Cu^I^_4_] cluster with the ligand MBOZ. Without the support of iodide, the [Cu^I^_4_] cluster **2** shows two electrochemical oxidation processes at *E*_p_^a^ = −0.03 and +0.36 V without a reduction process. This contrasts with the oxidation process observed at *E*_p_^a^ = −0.03 and +0.22 V for complex **6**. As for complex **4** and **4**′, their oxidation potentials are similar; they show two electrochemical oxidation processes at *E*_p_^a^ = +0.36 and +0.49 V (*E*_p_^a^ of **4**′ = +0.35 and +0.48 V) without a reduction process. (The oxidation-reduction potentials of complex **2**, **3**, **4**, **4**′, **6**, and **7** are shown in Table 1) It is not difficult to observe through a cyclic voltammogram that the oxidation and reduction peak area of complex **3** is larger than others, which means that when complex **3** reacts on the electrode surface, its electron transfer amount is maximized (Figure S11). And complex **3** is the only one that undergoes oxidation processes at both the ligand center and cluster core (Figures S7 and S10). The above result indicates that complex **3** has a higher capability to lose and gain electrons upon oxidation/reduction than complexes **2**, **4**, **4**′, **6**, and **7**. The chemical redox stability of complex **3** was checked by electrochemical cycling tests (Figure S8). 

**Figure 4:**
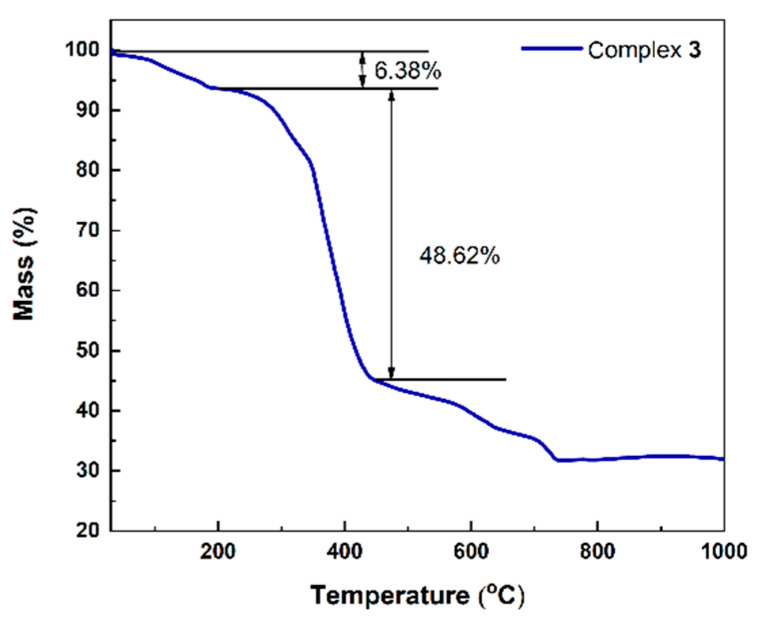
Thermogravimetric analysis curve of complex **3**.

The thermal stability of complex **3** was examined by the technique of thermogravimetric analysis (Figure 4). The first weight loss process occurs at 25–190 °C with a weight loss of about 6.38%. It is attributed to the escape of solvent molecules in the crystal sample. The second weight loss process occurs at 220–450 °C with a weight loss of about 48.62%. The significant weight loss is believed to result from the damage to the complex. According to the test results of differential scanning calorimetry, we can conclude that complex **3** did not undergo a significant phase transition below 180 °C (Figure S12). The result shows that complex **3** is stable for thermal catalytic reactions.

**Figure 5:**
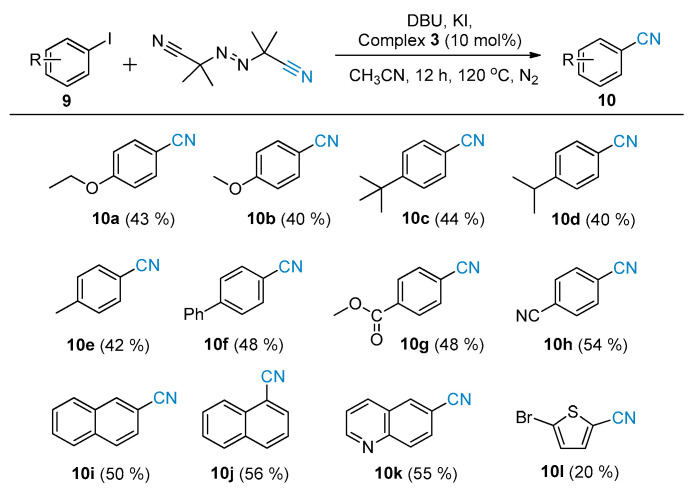
Scope of iodobenzenes with respect to compound **10** ^a^. ^a^ Reaction conditions: **9** (0.2 mmol), AIBN (0.3 mmol), KI (0.6 mmol), DBU (0.6 mmol), complex **3** (10 mol%), CH_3_CN (2.0 mL), N_2_, 120 °C, 12 h, isolated yields.

Research on organic catalysis by transition-metal clusters has attracted more and more interest, for example, the catalytic cyanation reaction. Excessive cyanide anion might be unfavorable for catalytic cyanation [77]. The cyanide source has always been a key factor. 2,2′-Azobis(2-methylpropionitrile) (AIBN) was considered as one of the low-toxicity cyanide sources for coupling reactions [78,79,80]. Thus, the stirring of iodobenzenes (0.2 mmol), AIBN (0.3 mmol), complex **3** (10 mol%), KI (0.6 mmol), and 1,8-diazabicyclo [5.4.0]undec-7-ene (DBU, 0.6 mmol) in CH_3_CN for 12 h at 120 °C provided the corresponding products **10** in normal yields of 40–56% (Figure 5). The low yield of **10l** could be due to the variability of the bromide group on the substrate.

**Scheme 2:**
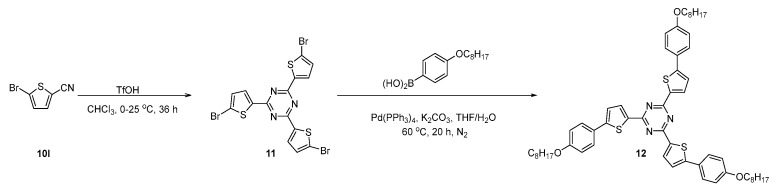
Synthetic route for 2,4,6-tris(5-(4-(octyloxy)phenyl)thiophen-2-yl)-1,3,5-triazine.

**Figure 6:**
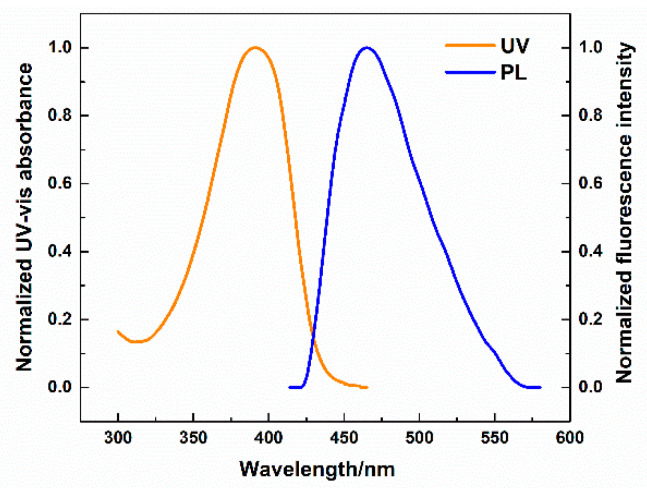
Studies of 2,4,6-tris(5-(4-(octyloxy)phenyl)thiophen-2-yl)-1,3,5-triazine for its fluorescence property.

Aryl nitriles possess versatile utilities and are useful building blocks in the organic synthesis for organic optoelectronic materials. The trimerization of compound **10l** using trifluoromethanesulfonic acid as catalyst provided 2,4,6-tris(5-bromothiophen-2-yl)-1,3,5-triazine (compound **11**). The reaction of compound **11** with three equivalents of 4-octoxyphenylboronic acid afforded 2,4,6-tris(5-(4-(octyloxy)phenyl)thiophen-2-yl)-1,3,5-triazine (compound **12**) in the presence of the catalyst Pd(PPh_3_)_4_ (Scheme 2). The CH_2_Cl_2_ solution of compound **12** (1.0 × 10^−5^ mol/L) emitted its characteristic fluorescence at 465 nm under the excitation at 391 nm (Figure 6), demonstrating its application potential in photo-electrochemistry.

## 3. Experimental Section

### 3.1. General

**Chemicals.** Unless otherwise stated, all inorganic reactions and manipulations are performed under air atmosphere. Complex [Cu(CH_3_CN)_4_]BF_4_, 2,4,6-tris(5-bromothiophen-2-yl)-1,3,5-triazine and (4-(octyloxy)phenyl)boronic acid were prepared according to the reported methods [81,82,83,84]. We characterized compounds by SC-XRD [85,86,87], XRD, and NMR; and some of these compounds were analyzed by CV (cyclic voltammetry) [88], TGA (thermogravimetric analysis), DSC (differential scanning calorimetry), elemental analysis, UV−vis spectra and fluorescence spectra (showed in Supplementary Materials).

### 3.2. Synthesis Procedures

#### 3.2.1. Synthesis of Complex **2**, **3**, **4**, **4**′, **6**, **7**

The synthesis procedure of complex **1**, **5**, and **8** can be found in the Supporting Information.

**[Cu^I^_4_(MBIZ)_4_(PPh_3_)_2_]·3.5THF (2).** The PPh_3_ (26.20 mg, 0.1 mmol) was added to a colorless solution of complex **1** (67.42 mg, 0.05 mmol) in CH_3_CN/THF (2/2 mL). The reaction mixture was stirred at 25 °C for 15 min. The solution was filtered and evaporated at room temperature, then the product was deposited as colorless crystals in three days (38.42 mg, 31.31%). ^1^H NMR (400 MHz, CD_3_CN) δ 9.88 (s, 4H), 7.42 (t, *J* = 8.7 Hz, 12H), 7.21–7.12 (m, 8H), 7.10–7.02 (m, 18H), 7.01–6.85 (m, 8H), 3.67 (s, 14H), 1.89–1.74 (m, 14H). ^13^C NMR (101 MHz, CD_3_CN) δ 134.29, 129.80, 128.67. Anal. Calcd. for C_78_H_78_Cu_4_N_8_O_3.5_P_2_S_4_: C, 57.27; H, 4.81; N, 6.85. Found: C, 57.75; H, 4.97; N, 6.73.

**[Cu^I^_4_(MBIZ)_3_(PPh_3_)_3_I]·3THF·CH_3_CN (3).** The Et_4_NI (25.72 mg, 0.1 mmol) was added to a colorless solution of complex **1** (67.42 mg, 0.05 mmol) in CH_3_CN/THF (2/2 mL). The reaction mixture was stirred at 25 °C for 15 min to form a pale yellow solution. The resulting pale yellow solution was treated with PPh_3_ (26.20 mg, 0.1 mmol) and stirred for 15 min. The colorless solution was filtered and evaporated at room temperature, then the product was deposited as colorless crystals in three days (47.26 mg, 33.64%). ^1^H NMR (400 MHz, CDCl_3_) δ 8.23 (s, 3H), 7.07–6.32 (m, 57H). ^13^C NMR (101 MHz, CDCl_3_) δ 134.29, 129.80, 128.67. Anal. Calcd. for C_89_H_87_Cu_4_IN_7_O_3_P_3_S_3_: C, 57.08; H, 4.68; N, 5.24. Found: C, 57.85; H, 4.82; N, 5.31.

**[Cu^I^_4_(MBIZ)_6_(PPh_3_)_4_](BF_4_)_4_ (4).** 2-mercaptobenzoxazole (MBIZ) (15.00 mg, 0.1 mmol) and HBF_4_ (13.17 mg, 0.15 mmol) were dissolved in THF (2 mL) by stirring for 15 min. The solution of [Cu(CH_3_CN)]BF_4_ (31.50 mg, 0.1 mmol) in MeCN (2 mL) was added to the solution of MBIZ dropwise. The mixture was stirred at 25 °C for 15 min. The resulting colorless solution was treated with PPh_3_ (26.20 mg, 0.1 mmol) and stirred for 15 min. The solution was filtered and evaporated at room temperature, then the product was deposited as colorless microcrystals in three days (28.52 mg, 44.00%). The microcrystals were dissolved in CH_3_CN + CHCl_3_ (4 mL) and sonicated for 30 min. The solution was filtered, and the filtrate was treated with Et_2_O (10 mL) to obtain some white precipitate as the product. ^1^H NMR (400 MHz, CD_3_CN/CDCl_3_) δ 11.69 (s, 12H), 7.40–7.26 (m, 60H), 7.25–7.12 (m, 24H). ^13^C NMR (101 MHz, CD_3_CN/CDCl_3_) δ 133.40, 130.10, 128.80. Anal. Calcd. for C_114_H_96_B_4_Cu_4_F_16_N_12_P_4_S_6_: C, 53.66; H, 3.79; N, 6.59. Found: C, 53.48; H, 3.84; N, 6.54.

**[Cu^I^_4_(MBIZ)_6_(P(*p*-CH_3_-Ph)_3_)_4_]·(BF_4_)_4_ (4′).** 2-mercaptobenzoxazole (MBIZ) (15.00 mg, 0.1 mmol) and HBF_4_ (13.17 mg, 0.15 mmol) were dissolved in THF (2 mL) by stirring for 15 min. The solution of [Cu(CH_3_CN)]BF_4_ (31.50 mg, 0.1 mmol) in MeCN (2 mL) was added to the solution of MBIZ dropwise. The mixture was stirred at 25 °C for 15 min. The resulting colorless solution was treated with tri(*p*-tolyl)phosphine (30.40 mg, 0.1 mmol) and stirred for 15 min. The solution was filtered and evaporated at room temperature, then the product was deposited as colorless microcrystals in three days (30.36 mg, 43.71%). The microcrystals were dissolved in CH_3_CN + CHCl_3_ (4 mL) and sonicated for 30 min. The solution was filtered, and the filtrate was treated with Et_2_O (10 mL) to obtain some white precipitate as the product. ^1^H NMR (400 MHz, CDCl_3_) δ 11.07 (s, 12H), 7.08 (d, *J* = 15.4 Hz, 24H), 6.78–6.59 (m, 48H), 1.93 (s, 36H). ^13^C NMR (101 MHz, CDCl_3_) δ 156.61, 139.40, 132.04, 130.81, 129.09, 127.77, 123.83, 111.74, 21.24. Anal. Calcd. for C_126_H_120_B_4_Cu_4_F_16_N_12_P_4_S_6_: C, 55.64; H, 4.45; N, 6.18. Found: C, 55.37; H, 4.48; N, 6.14.

**[Cu^I^_4_(MBOZ)_4_(PPh_3_)_4_] (6).** The PPh_3_ was added to the colorless solution of complex **5** (94.84 mg, 0.05 mmol) in CH_3_CN/THF (2/2 mL) (26.20 mg, 0.1 mmol). The reaction mixture was stirred at 25 °C for 15 min. The solution was filtered and evaporated at room temperature, then the product was deposited as colorless crystals in three days (46.15 mg, 32.31%). ^1^H NMR (400 MHz, CDCl_3_) δ 7.39 (t, *J* = 8.6 Hz, 24H), 7.20–7.02 (m, 36H), 6.97–6.55 (m, 16H). ^13^C NMR (101 MHz, CDCl_3_) δ 141.36, 133.88, 129.25, 128.33. Anal. Calcd. for C_100_H_76_Cu_4_N_4_O_4_P_4_S_4_: C, 63.08; H, 4.02; N, 2.94. Found: C, 62.79; H, 4.09; N, 2.91.

**[Cu^I^_4_(MBOZ)_3_(PPh_3_)_3_I]·CHCl_3_ (7).** The Et_4_NI (25.72 mg, 0.1 mmol) was added to the colorless solution of complex **5** (94.84 mg, 0.05 mmol) in CH_3_CN/THF (2/2 mL). The reaction mixture was stirred at 25 °C for 15 min to form a pale yellow solution. The resulting pale yellow solution was treated with PPh_3_ (26.20 mg, 0.1 mmol) and stirred for 15 min. The colorless solution was filtered and evaporated at room temperature, then the product was deposited as colorless microcrystals in three days (52.32 mg, 40.13%). The microcrystals were recrystallized from CH_3_CN/CHCl_3_ to afford the product as some colorless crystals. ^1^H NMR (400 MHz, CDCl_3_) δ 7.60–7.35 (m, 18H), 7.06 (s, 27H), 6.84 (d, *J* = 4.7 Hz, 6H), 6.54–6.34 (m, 6H). ^13^C NMR (101 MHz, CDCl_3_) δ 151.02, 133.92, 129.35, 128.28, 122.65, 122.34, 116.94, 109.51. Anal. Calcd. for C_76_H_58_Cl_3_Cu_4_IN_3_O_3_P_3_S_3_: C, 52.53; H, 3.36; N, 2.42. Found: C, 53.05; H, 3.43; N, 2.44.

#### 3.2.2. General Procedure for the Synthesis of Compound **10**

Aromatic iodides **9** (0.20 mmol), Azodiisobutyronitrile (AIBN, 0.30 mmol, 49.26 mg), complex **3** (10% mmol, 32.00 mg), potassium iodide (0.60 mmol, 99.60 mg), 1,8-Diazabicyclo [5.4.0]undec-7-ene (DBU, 0.60 mmol, 91.34 mg), and CH_3_CN (2 mL) were mixed in a 50 mL Teflon screw-cap sealed tube. The mixture was vigorously stirred at 120 °C under N_2_ atmosphere for 12 h (oil bath). After cooling to room temperature, the reaction mixture was diluted with dichloromethane (20 mL), filtered through a pad of silica gel, and concentrated under reduced pressure. The crude product was purified on a silica gel column eluted with petroleum and ether/ethyl acetate (8:1 *v*/*v*) to yield the products **10**. NMR spectra are presented in pages S16–S27 (SI).

4-Ethoxybenzonitrile (**10a**): yield, 43% (11.10 mg); white solid. M.p. 62–63 °C. ^1^H NMR (400 MHz, CDCl_3_) δ 7.56 (d, *J* = 8.8 Hz, 2H), 6.92 (d, *J* = 8.8 Hz, 2H), 4.06 (q, *J* = 7.0 Hz, 2H), 1.43 (t, *J* = 7.0 Hz, 3H). ^13^C NMR (101 MHz, CDCl_3_) δ 162.34, 134.04, 119.41, 115.23, 103.72, 64.01, 14.65.

4-Methoxybenzonitrile (**10b**): yield, 40% (10.63 mg); pale yellow solid. M.p. 59–60 °C. ^1^H NMR (400 MHz, CDCl_3_) δ 7.58 (d, *J* = 8.4 Hz, 2H), 6.94 (d, *J* = 8.5 Hz, 2H), 3.85 (s, 3H). ^13^C NMR (101 MHz, CDCl_3_) δ 162.94, 134.09, 119.36, 114.85, 104.04, 55.66. 

4-*tert*-Butylbenzonitrile **(10c)**: yield, 44% (12.77 mg); colorless liquid; ^1^H NMR (400 MHz, CDCl_3_) δ 7.58 (d, *J* = 8.4 Hz, 2H), 7.47 (d, *J* = 8.4 Hz, 2H), 1.32 (s, 9H). ^13^C NMR (101 MHz, CDCl_3_) δ 156.73, 132.05, 126.26, 119.26, 109.37, 35.35, 31.03.

4-Isopropylbenzonitrile (**10d**): yield, 40% (10.66 mg); colorless liquid; **^1^**H NMR (400 MHz, CDCl_3_) δ 7.57 (d, *J* = 8.0 Hz, 2H), 7.31 (d, *J* = 8.1 Hz, 2H), 3.01–2.91 (m, 1H), 1.25 (d, *J* = 6.8 Hz, 6H). ^13^C NMR (101 MHz, CDCl_3_) δ 154.48, 132.35, 127.42, 119.31, 109.69, 34.50, 23.66.

4-Methylbenzonitrile (**10e**): yield, 42% (9.80 mg); colorless liquid; ^1^H NMR (400 MHz, CDCl_3_) δ 7.52 (d, *J* = 8.1 Hz, 2H), 7.26 (d, *J* = 8.0 Hz, 2H), 2.41 (s, 3H). ^13^C NMR (101 MHz, CDCl_3_) δ 143.72, 131.98, 129.83, 119.14, 109.22, 21.80. 

*p*-Phenylbenzonitrile (**10f**): yield, 48% (17.20 mg); white solid; ^1^H NMR (400 MHz, CDCl_3_) δ 7.71 (q, *J* = 8.3 Hz, 4H), 7.59 (d, *J* = 7.6 Hz, 2H), 7.49 (t, *J* = 7.5 Hz, 2H), 7.43 (t, *J* = 7.2 Hz, 1H).^13^C NMR (101 MHz, CDCl_3_) δ 145.77, 139.27, 132.71, 129.23, 128.78, 127.84, 127.34, 119.07, 111.00.

4-Cyanobenzoicacidmethylester (**10g**): yield, 48% (14.40 mg); white solid; ^1^H NMR (400 MHz, CDCl_3_) δ 8.12 (d, *J* = 8.3 Hz, 2H), 7.73 (d, *J* = 8.3 Hz, 2H), 3.95 (s, 3H). ^13^C NMR (101 MHz, CDCl_3_) δ 165.52, 134.00, 132.32, 130.19, 118.06, 116.47, 52.83. 

1,4-Benzodinitrile (**10h**): yield, 54% (13.80 mg); white solid; ^1^H NMR (400 MHz, CDCl_3_) δ 7.80 (s, 4H). ^13^C NMR (101 MHz, CDCl_3_) δ 132.93, 117.14, 116.84. 

2-Cyanonaphthylene (**10i**): yield, 50% (15.30 mg); white solid; ^1^H NMR (400 MHz, CDCl_3_) δ 8.22 (s, 1H), 7.90 (t, *J* = 8.7 Hz, 3H), 7.68–7.56 (m, 3H). ^13^C NMR (101 MHz, CDCl_3_) δ 134.74, 134.26, 132.33, 129.30, 129.15, 128.52, 128.16, 127.76, 126.44, 119.37, 109.45.

1-Cyanonaphthalene (**10j**): yield, 56% (17.20 mg); white solid; ^1^H NMR (400 MHz, CDCl_3_) δ 8.24 (d, *J* = 8.3 Hz, 1H), 8.08 (d, *J* = 8.3 Hz, 1H), 7.92 (t, *J* = 6.6 Hz, 2H), 7.69 (t, *J* = 7.6 Hz, 1H), 7.62 (t, *J* = 7.5 Hz, 1H), 7.52 (t, *J* = 7.7 Hz, 1H). ^13^C NMR (101 MHz, CDCl_3_) δ 133.40, 133.02, 132.75, 132.45, 128.77, 128.71, 127.66, 125.25, 125.04, 117.95, 110.27. 

Quinoline-6-carbonitrile (**10k**): yield, 55% (16.90 mg); white solid; ^1^H NMR (400 MHz, CDCl_3_) δ 9.05 (s, 1H), 8.20 (dd, *J* = 13.6, 8.3 Hz, 3H), 7.84 (d, *J* = 8.7 Hz, 1H), 7.54 (dd, *J* = 8.2, 4.1 Hz, 1H). ^13^C NMR (101 MHz, CDCl_3_) δ 153.32, 149.17, 136.53, 134.23, 131.14, 130.25, 127.66, 122.85, 118.59, 110.50. 

5-Bromothiophene-2-acetonitrile (**10l**): yield, 20% (7.52 mg); yellow liquid; ^1^H NMR (400 MHz, CDCl_3_) δ 7.37 (d, *J* = 4.0 Hz, 1H), 7.08 (d, *J* = 4.0 Hz, 1H). ^13^C NMR (101 MHz, CDCl_3_) δ 138.01, 130.70, 120.08, 113.06, 111.17.

#### 3.2.3. Experimental Procedures for the Synthesis of Compounds **11** and **12**

2,4,6-tris(5-bromothiophen-2-yl)-1,3,5-triazine (**11**). Trifluoromethanesulfonic acid was added to a stirred solution of 10l (7 mmol, 1.32 g) in dry CHCl_3_ (20 mL) (14 mmol, 2.10 g) at 0 °C. The mixture was stirred for 36 h at room temperature. After removal of the solvent under reduced pressure, the residue was neutralized with an aqueous NaHCO_3_. The formed precipitate was collected by filtration, washed with water, methanol, acetone, and hexane in this order, and then dried under vacuum to afford 11 as an off-white solid (yield = 2.56 g, 65%). ^1^H NMR (400 MHz, CDCl_3_) δ 7.97 (d, J = 4.0 Hz, 1H), 7.17 (d, J = 4.0 Hz, 1H). ^13^C NMR (101 MHz, CDCl_3_) δ 166.86, 142.30, 132.17, 131.81, 120.85.

2,4,6-tris(5-(4-(octyloxy)phenyl)thiophen-2-yl)-1,3,5-triazine (**12**). The Pd(PPh_3_)_4_ (0.05 mmol, 0.06 g) and aqueous K_2_CO_3_ (2.0 M, 8 mL; N_2_ bubbled before use) were added to a solution of (4-(octyloxy)phenyl)boronic acid (2 mmol, 0.5 g) and 2,4,6-tris(5-bromothiophen-2-yl)-1,3,5-triazine (0.5 mmol, 0.28 g) in dry THF (20 mL). The mixture was vigorously stirred for 20 h at 60 °C under N_2_ atmosphere. The mixture was cooled and poured into a separatory funnel together with water. The aqueous solution was extracted with CH_2_Cl_2_ (3 × 50 mL), and the combined organic layers were dried over anhydrous Na_2_SO_4_ overnight. The solvent was removed and the crude product was purified on a silica gel column eluted with petroleum and CH_2_Cl_2_ (2:1 *v*/*v*) to afford 2,4,6-tris(5-(4-(octyloxy)phenyl)thiophen-2-yl)-1,3,5-triazine as a light yellow solid in yield 54% (0.25 g). ^1^H NMR (400 MHz, CDCl_3_) δ 8.04 (d, *J* = 3.8 Hz, 1H), 7.52 (d, *J* = 8.6 Hz, 2H), 7.15 (t, 1H), 6.81 (d, *J* = 8.7 Hz, 2H), 3.87 (t, *J* = 6.5 Hz, 2H), 1.81–1.61 (m, 2H), 1.42–1.29 (m, 2H), 1.29–1.08 (m, 8H), 0.80 (t, *J* = 6.6 Hz, 3H). ^13^C NMR (101 MHz, CDCl_3_) δ 167.24, 159.68, 151.17, 139.28, 132.75, 127.48, 126.66, 123.26, 114.99, 68.27, 31.98, 29.55, 29.41, 26.20, 22.82, 14.28.

## 4. Conclusions

In summary, we described the synthesis of a series of tetrahedral copper-thiolate tetramers by the regulation of multi-nuclear copper clusters with PPh_3_. The insertion of iodide anion to the cluster improved the redox activity and stability, which was supported by the study of electrochemistry and thermogravimetric analysis. The reaction performance was demonstrated by the catalytic cyanation of iodobenzenes using AIBN as the cyanide source. The products show potential applications as luminescent layer materials.

## Figures and Tables

**Table 1:** Oxidation–reduction potential (vs. Fc^+^/Fc) of complex **2**, **3**, **4**, **4**′, **6**, and **7**.

Complex	*E*_p_^a^	*E*_p_^c^	Complex	*E*_p_^a^	*E*_p_^c^
**2**	−0.03 V, +0.36 V	-	**4**′	+0.35 V, +0.48 V	-
**3**	+0.11 V, +0.45 V	+0.25 V	**6**	−0.03 V, +0.22 V	-
**4**	+0.36 V, +0.49 V	-	**7**	+0.15 V, +0.21 V	-

## Data Availability

The data underlying this study are available in the published article and its online Supplementary Material.

## Supplementary Materials

The following supporting information can be downloaded at: https://www.mdpi.com/article/10.3390/molecules29174228/s1. Accession codes: CCDC 2336741, CCDC 2336735, CCDC 2374006, CCDC 2374007, CCDC 2374008, and CCDC 2336717 contain the supplementary crystallographic data for this paper. These data can be obtained free of charge via www.ccdc.cam.ac.uk/data_request/cif (accessed on 3 September 2024), or by emailing da-ta_request@ccdc.cam.ac.uk, or by contacting The Cambridge Crystallographic Data Centre, 12 Union Road, Cambridge CB2 1EZ, UK; Fax: +44-1223-336033.
