# Supplementary material for: Research on Synthesis, Structure, and Catalytic Performance of Tetranuclear Copper(I) Clusters Supported by 2-Mercaptobenz-zole-Type Ligands
Source: Molecules. 2024 Sep 6;29(17):4228. doi: 10.3390/molecules29174228 (PMC11396812; doi:10.3390/molecules29174228)

checkCIF/PLATON report

Structure factors have been supplied for datablock(s) full

THIS REPORT IS FOR GUIDANCE ONLY. IF USED AS PART OF A REVIEW PROCEDURE FOR PUBLICATION, IT SHOULD NOT REPLACE THE EXPERTISE OF AN EXPERIENCED CRYSTALLOGRAPHIC REFEREE.

No syntax errors found. CIF dictionary Interpreting this report

Datablock: full Complex 2

Bond precision: C-C = 0.0063 A Wavelength=1.54184

Cell:	a=14.9470(6)	b=15.3365(4)	c=18.2930(6)
	alpha=84.801(2)	beta=88.059(3)	gamma=64.577(3)
Temperature:	150 K		

	Calculated	Reported
Volume	3771.7(2)	3771.7(2)
Space group	P -1	P -1
Hall group	-P 1	-P 1
Moiety formula	2(C64 H50 Cu4 N8 P2 S4), 7(C4 H8 O)	C64 H50 Cu4 N8 P2 S4, 3.5(C4 H8 O)
Sum formula	C156 H156 Cu8 N16 O7 P4 S8	C78 H78 Cu4 N8 O3.50 P2 S4
Mr	3255.74	1627.82
Dx,g cm-3	1.433	1.433
Z	1	2
Mu (mm-1)	3.130	3.130
F000	1680.0	1680.0
F000'	1671.50	
h,k,lmax	18,18,22	18,18,22
Nref	14304	14213
Tmin,Tmax	0.699,0.709	0.539,1.000
Tmin'	0.634	

Correction method= # Reported T Limits: Tmin=0.539 Tmax=1.000
AbsCorr = MULTI-SCAN

Data completeness= 0.994 Theta(max)= 69.993

R(reflections)= 0.0568(11445) wR2(reflections)= 0.1763(14213)

S = 1.076 Npar= 1058

The following ALERTS were generated. Each ALERT has the format
test-name_ALERT_alert-type_alert-level.
Click on the hyperlinks for more details of the test.

Alert level C

```
PLAT042_ALERT_1_C Calc. and Reported MoietyFormula Strings  Differ     Please Check
              Calc: 2(C64 H50 Cu4 N8 P2 S4), 7(C4 H8 O)
              Rep.: C64 H50 Cu4 N8 P2 S4, 3.5(C4 H8 O)
PLAT341_ALERT_3_C Low Bond Precision on  C-C Bonds ...............     0.0063 Ang.
PLAT906_ALERT_3_C Large K Value in the Analysis of Variance ......      2.358 Check
PLAT911_ALERT_3_C Missing FCF Refl Between Thmin & STh/L=    0.600         16 Report
                0  3  0,    0 -2  1,    0  2  1,  -2  0  2,  -1  1  2,  10 17  9,
              -13-11 12,    6 -8 14,    8 15 14, -10 -7 17,    4 -7 17,   4 10 19,
                3  9 20,    4  9 20,  -1  3 21,    3  5 21,
```

Alert level G

```
PLAT002_ALERT_2_G Number of Distance or Angle Restraints on AtSite         35 Note
PLAT003_ALERT_2_G Number of Uiso or U(i,j) Restrained non-H Atoms          44 Report
PLAT007_ALERT_5_G Number of Unrefined Donor-H Atoms ..............          4 Report
              H2A   H4A   H6A   H8A
PLAT045_ALERT_1_G Calculated and Reported Z Differ by a Factor ...      0.500 Check
PLAT172_ALERT_4_G The CIF-Embedded .res File Contains DFIX Records         15 Report
PLAT177_ALERT_4_G The CIF-Embedded .res File Contains DELU Records          2 Report
PLAT178_ALERT_4_G The CIF-Embedded .res File Contains SIMU Records          2 Report
PLAT188_ALERT_3_G A Non-default SIMU Restraint Value has been used     0.0020 Report
PLAT188_ALERT_3_G A Non-default SIMU Restraint Value has been used     0.0050 Report
PLAT192_ALERT_3_G A Non-default DELU Restraint Value for First Par     0.0020 Report
PLAT192_ALERT_3_G A Non-default DELU Restraint Value for SecondPar     0.0020 Report
PLAT192_ALERT_3_G A Non-default DELU Restraint Value for First Par     0.0050 Report
PLAT192_ALERT_3_G A Non-default DELU Restraint Value for SecondPar     0.0050 Report
PLAT232_ALERT_2_G Hirshfeld Test Diff (M-X)  Cu1      --S4        .      13.6 s.u.
PLAT232_ALERT_2_G Hirshfeld Test Diff (M-X)  Cu2      --S3        .      11.4 s.u.
PLAT232_ALERT_2_G Hirshfeld Test Diff (M-X)  Cu2      --S4        .      16.0 s.u.
PLAT232_ALERT_2_G Hirshfeld Test Diff (M-X)  Cu3      --S1        .      27.8 s.u.
PLAT232_ALERT_2_G Hirshfeld Test Diff (M-X)  Cu3      --N7        .       6.3 s.u.
PLAT232_ALERT_2_G Hirshfeld Test Diff (M-X)  Cu4      --S2        .       5.6 s.u.
PLAT232_ALERT_2_G Hirshfeld Test Diff (M-X)  Cu4      --S4        .      55.6 s.u.
PLAT299_ALERT_4_G Atom Site Occupancy Constrained at .............        0.5 Check
              O2      C69     C70     C71     C72     H69A    H69B    H70A
              H70B    H71A    H71B    H72A    H72B
PLAT302_ALERT_4_G Anion/Solvent/Minor-Residue Disorder (Resd    2)       100% Note
PLAT302_ALERT_4_G Anion/Solvent/Minor-Residue Disorder (Resd    3)       100% Note
PLAT302_ALERT_4_G Anion/Solvent/Minor-Residue Disorder (Resd    4)       100% Note
PLAT302_ALERT_4_G Anion/Solvent/Minor-Residue Disorder (Resd    5)       100% Note
PLAT302_ALERT_4_G Anion/Solvent/Minor-Residue Disorder (Resd    6)       100% Note
PLAT302_ALERT_4_G Anion/Solvent/Minor-Residue Disorder (Resd    7)       100% Note
PLAT302_ALERT_4_G Anion/Solvent/Minor-Residue Disorder (Resd    8)       100% Note
PLAT304_ALERT_4_G Non-Integer Number of Atoms in ..... (Resd    2)      11.10 Check
PLAT304_ALERT_4_G Non-Integer Number of Atoms in ..... (Resd    3)       6.50 Check
PLAT304_ALERT_4_G Non-Integer Number of Atoms in ..... (Resd    4)       7.72 Check
PLAT304_ALERT_4_G Non-Integer Number of Atoms in ..... (Resd    5)       9.53 Check
PLAT304_ALERT_4_G Non-Integer Number of Atoms in ..... (Resd    6)       1.90 Check
PLAT304_ALERT_4_G Non-Integer Number of Atoms in ..... (Resd    7)       5.28 Check
PLAT304_ALERT_4_G Non-Integer Number of Atoms in ..... (Resd    8)       3.47 Check
```

```
PLAT398_ALERT_2_G Deviating  C-O-C   Angle From 120 for O1       .      106.2 Degree
PLAT398_ALERT_2_G Deviating  C-O-C   Angle From 120 for O2       .      107.3 Degree
PLAT398_ALERT_2_G Deviating  C-O-C   Angle From 120 for O3       .      105.3 Degree
PLAT398_ALERT_2_G Deviating  C-O-C   Angle From 120 for O4       .      108.2 Degree
PLAT398_ALERT_2_G Deviating  C-O-C   Angle From 120 for O1'      .      108.7 Degree
PLAT398_ALERT_2_G Deviating  C-O-C   Angle From 120 for O3'      .      108.7 Degree
PLAT398_ALERT_2_G Deviating  C-O-C   Angle From 120 for O4'      .      108.3 Degree
PLAT411_ALERT_2_G Short Inter H...H Contact  H28A     ..H66C     .       2.14 Ang.
                                                      x,y,z  =      1_555 Check
PLAT790_ALERT_4_G Centre of Gravity not Within Unit Cell: Resd.  #          2 Note
              C4 H8 O
PLAT790_ALERT_4_G Centre of Gravity not Within Unit Cell: Resd.  #          6 Note
              C4 H8 O
PLAT794_ALERT_5_G Tentative Bond Valency for Cu3       (I)       .       0.96 Info
PLAT860_ALERT_3_G Number of Least-Squares Restraints ............        721 Note
PLAT883_ALERT_1_G No Info/Value for _atom_sites_solution_primary .     Please Do !
PLAT899_ALERT_4_G SHELXL2018 is Deprecated and Succeeded by SHELXL     2019/3 Note
PLAT910_ALERT_3_G Missing # of FCF Reflection(s) Below Theta(Min).          4 Note
                1  0  0,   0  1  0,   1  1  0,   0  0  1,
PLAT912_ALERT_4_G Missing # of FCF Reflections Above STh/L=  0.600         71 Note
PLAT933_ALERT_2_G Number of HKL-OMIT Records in Embedded .res File          5 Note
               -1  1  2,   0  2  1,  -2  0  2,   0  3  0,   0 -2  1,
PLAT941_ALERT_3_G Average HKL Measurement Multiplicity ..........        2.3 Low
PLAT961_ALERT_5_G Dataset Contains no Negative Intensities .......     Please Check
PLAT967_ALERT_5_G Note: Two-Theta Cutoff Value in Embedded .res ..      140.0 Degree
PLAT969_ALERT_5_G The 'Henn et al.' R-Factor-gap value ..........      4.415 Note
              Predicted wR2: Based on SigI**2  3.99 or SHELX Weight 16.39
PLAT978_ALERT_2_G Number C-C Bonds with Positive Residual Density.          1 Info
PLAT992_ALERT_5_G Repd & Actual _reflns_number_gt Values Differ by          2 Check
```

---

0 **ALERT level A** = Most likely a serious problem - resolve or explain
0 **ALERT level B** = A potentially serious problem, consider carefully
4 **ALERT level C** = Check. Ensure it is not caused by an omission or oversight
58 **ALERT level G** = General information/check it is not something unexpected

3 ALERT type 1 CIF construction/syntax error, inconsistent or missing data
19 ALERT type 2 Indicator that the structure model may be wrong or deficient
12 ALERT type 3 Indicator that the structure quality may be low
22 ALERT type 4 Improvement, methodology, query or suggestion
6 ALERT type 5 Informative message, check

It is advisable to attempt to resolve as many as possible of the alerts in all categories. Often the minor alerts point to easily fixed oversights, errors and omissions in your CIF or refinement strategy, so attention to these fine details can be worthwhile. In order to resolve some of the more serious problems it may be necessary to carry out additional measurements or structure refinements. However, the purpose of your study may justify the reported deviations and the more serious of these should normally be commented upon in the discussion or experimental section of a paper or in the "special_details" fields of the CIF. checkCIF was carefully designed to identify outliers and unusual parameters, but every test has its limitations and alerts that are not important in a particular case may appear. Conversely, the absence of alerts does not guarantee there are no aspects of the results needing attention. It is up to the individual to critically assess their own results and, if necessary, seek expert advice.

Publication of your CIF in IUCr journals

A basic structural check has been run on your CIF. These basic checks will be run on all CIFs submitted for publication in IUCr journals (*Acta Crystallographica*, *Journal of Applied Crystallography*, *Journal of Synchrotron Radiation*); however, if you intend to submit to *Acta Crystallographica Section C* or *E* or *IUCrData*, you should make sure that full publication checks are run on the final version of your CIF prior to submission.

Publication of your CIF in other journals

Please refer to the *Notes for Authors* of the relevant journal for any special instructions relating to CIF submission.

PLATON version of 15/07/2024; check.def file version of 15/07/2024

Datablock full - ellipsoid plot

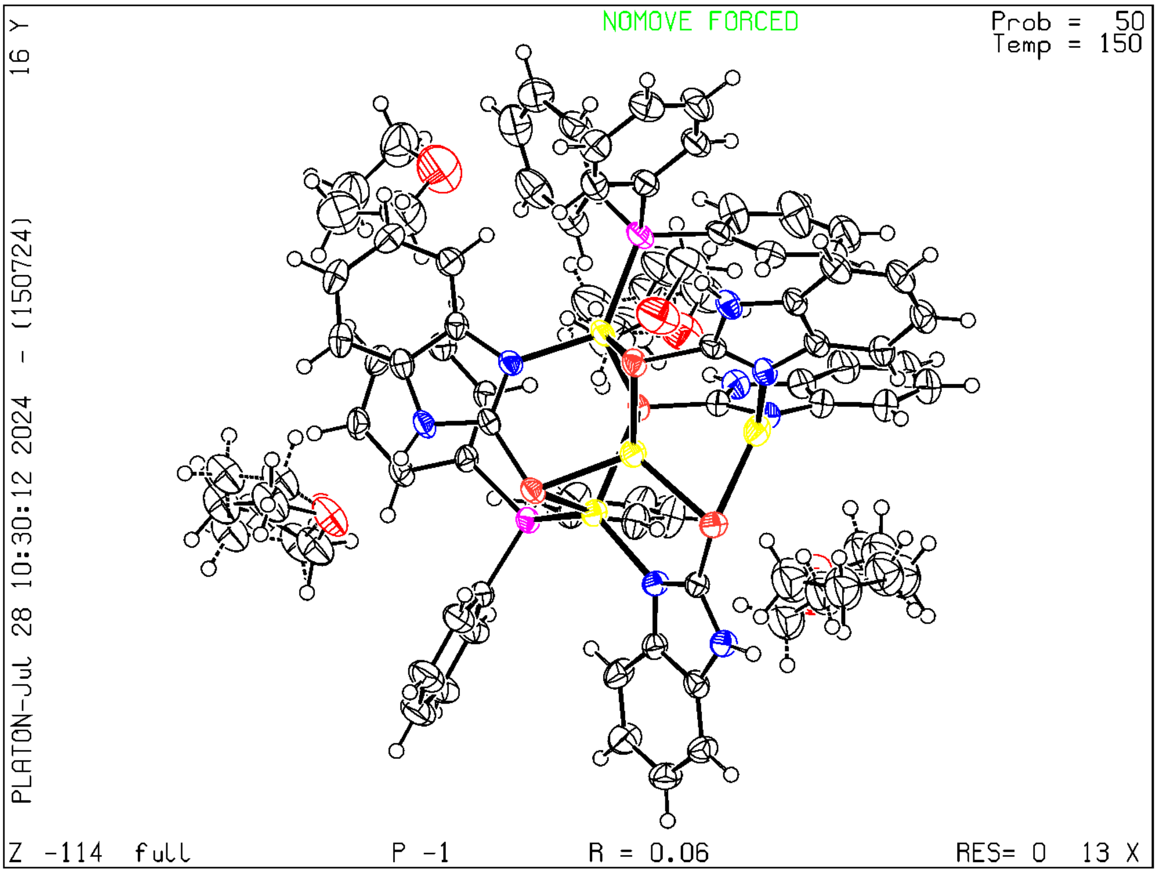

checkCIF/PLATON report

Structure factors have been supplied for datablock(s) full

THIS REPORT IS FOR GUIDANCE ONLY. IF USED AS PART OF A REVIEW PROCEDURE FOR PUBLICATION, IT SHOULD NOT REPLACE THE EXPERTISE OF AN EXPERIENCED CRYSTALLOGRAPHIC REFEREE.

No syntax errors found.   CIF dictionary   Interpreting this report

Datablock: full   Complex 3

Bond precision: C-C = 0.0151 A   Wavelength=1.54184

Cell:	a=16.9492(6)	b=16.9492(6)	c=17.6895(8)
	alpha=90	beta=90	gamma=120
Temperature:	150 K		

	Calculated	Reported
Volume	4400.9(4)	4400.9(4)
Space group	P -3	P -3
Hall group	-P 3	-P 3
Moiety formula	C75 H60 Cu4 I N6 P3 S3, 3(C4 H8 O), C2 H3 N	C75 H60 Cu4 I N6 P3 S3, 3(C4 H8 O), C2 H3 N
Sum formula	C89 H87 Cu4 I N7 O3 P3 S3	C89 H87 Cu4 I N7 O3 P3 S3
Mr	1872.85	1872.80
Dx,g cm-3	1.413	1.413
Z	2	2
Mu (mm-1)	5.422	5.422
F000	1912.0	1912.0
F000'	1903.19	
h,k,lmax	20,20,21	20,20,21
Nref	5488	5472
Tmin,Tmax	0.548,0.648	0.459,1.000
Tmin'	0.322	

Correction method= # Reported T Limits: Tmin=0.459 Tmax=1.000
AbsCorr = MULTI-SCAN

Data completeness= 0.997   Theta(max)= 68.997

R(reflections)= 0.0802(4898)   wR2(reflections)= 0.2195(5472)

S = 1.042   Npar= 332

The following ALERTS were generated. Each ALERT has the format
test-name_ALERT_alert-type_alert-level.
Click on the hyperlinks for more details of the test.

Alert level B

```
PLAT601_ALERT_2_B Unit Cell Contains Solvent Accessible VOIDS of .        172 Ang**3
```

Author Response: The highest difference peak 2.60 (Q1) is located 0.82 angstrom away from the I1 atom. The peaks Q3 to Q5 are located 0.82-0.84 angstrom away from the Cu1 and Cu2 atoms. They should be the residual peaks of the heavy atoms I and Cu by the copper radiation source. The second difference peak 1.87 (Q2) could be defined as a solvent atom. However, it is located at a special position, from which a six atoms ring will be formed by "grow" with the other five atoms gained by symmetry. The distances between adjacent peaks are 1.32 angstroms. The ring seems like a cyclohexane ring with bond lengths being 1.32 angstrom. It is a bit odd, since cyclohexane was not used in our reaction or crystalization. Thus, the peak Q2 is not defined in our structure.

Alert level C

```
PLAT241_ALERT_2_C High   'MainMol' Ueq as Compared to Neighbors of        C24 Check
PLAT260_ALERT_2_C Large Average Ueq of Residue Including        O1      0.125 Check
PLAT342_ALERT_3_C Low Bond Precision on  C-C Bonds ..............    0.01507 Ang.
PLAT360_ALERT_2_C Short  C(sp3)-C(sp3) Bond  C29       - C30       .      1.38 Ang.
PLAT911_ALERT_3_C Missing FCF Refl Between Thmin & STh/L=    0.600         14 Report
               -1  2  0,   0  2  0,  -1  3  0,  -8 16  1,  -1  1  2,   0  1  2,
                1  1  2,  -2  2  2,   0  2  2,  -1  3  2,   0  1  4,  -1  2  4,
                0  3  4,   0  2  6,
PLAT918_ALERT_3_C Reflection(s) with I(obs) much Smaller I(calc) .          1 Check
PLAT971_ALERT_2_C Check Calcd Resid. Dens.  0.73Ang From I1             2.47 eA-3
PLAT971_ALERT_2_C Check Calcd Resid. Dens.  0.73Ang From I1             1.77 eA-3
PLAT972_ALERT_2_C Check Calcd Resid. Dens.  0.66Ang From I1            -1.74 eA-3
```

Alert level G

```
PLAT003_ALERT_2_G Number of Uiso or U(i,j) Restrained non-H Atoms           5 Report
PLAT007_ALERT_5_G Number of Unrefined Donor-H Atoms .............           1 Report
              H2A
PLAT083_ALERT_2_G SHELXL Second Parameter in WGHT  Unusually Large      27.94 Why ?
PLAT177_ALERT_4_G The CIF-Embedded .res File Contains DELU Records          1 Report
PLAT178_ALERT_4_G The CIF-Embedded .res File Contains SIMU Records          1 Report
PLAT188_ALERT_3_G A Non-default SIMU Restraint Value has been used     0.0050 Report
PLAT192_ALERT_3_G A Non-default DELU Restraint Value for First Par     0.0050 Report
PLAT192_ALERT_3_G A Non-default DELU Restraint Value for SecondPar     0.0050 Report
PLAT232_ALERT_2_G Hirshfeld Test Diff (M-X)  I1       --Cu1       .      17.1 s.u.
PLAT232_ALERT_2_G Hirshfeld Test Diff (M-X)  I1       --Cu2       .      12.7 s.u.
PLAT860_ALERT_3_G Number of Least-Squares Restraints ............          40 Note
PLAT883_ALERT_1_G No Info/Value for _atom_sites_solution_primary .     Please Do !
PLAT899_ALERT_4_G SHELXL2018 is Deprecated and Succeeded by SHELXL     2019/3 Note
```

```
PLAT910_ALERT_3_G Missing # of FCF Reflection(s) Below Theta(Min).          2 Note
                0  1  0,   0  0  1,
PLAT933_ALERT_2_G Number of HKL-OMIT Records in Embedded .res File         14 Note
                0  1  2,  -1  2  0,   0  2  6,   0  2  0,   1  1  2,  -2  2  2,
                0  1  4,  -1  3  0,   0  3  4,  -1  1  2,   0  2  2,  -1  2  4,
               -1  3  2,  -8 16  1,
PLAT961_ALERT_5_G Dataset Contains no Negative Intensities .......     Please Check
PLAT967_ALERT_5_G Note: Two-Theta Cutoff Value in Embedded .res ..      138.0 Degree
PLAT969_ALERT_5_G The 'Henn et al.' R-Factor-gap value ..........      8.061 Note
              Predicted wR2: Based on SigI**2  2.72 or SHELX Weight 21.07
PLAT978_ALERT_2_G Number C-C Bonds with Positive Residual Density.          1 Info
```

```
   0 ALERT level A = Most likely a serious problem - resolve or explain
   1 ALERT level B = A potentially serious problem, consider carefully
   9 ALERT level C = Check. Ensure it is not caused by an omission or oversight
  19 ALERT level G = General information/check it is not something unexpected

   1 ALERT type 1 CIF construction/syntax error, inconsistent or missing data
  13 ALERT type 2 Indicator that the structure model may be wrong or deficient
   8 ALERT type 3 Indicator that the structure quality may be low
   3 ALERT type 4 Improvement, methodology, query or suggestion
   4 ALERT type 5 Informative message, check
```

It is advisable to attempt to resolve as many as possible of the alerts in all categories. Often the minor alerts point to easily fixed oversights, errors and omissions in your CIF or refinement strategy, so attention to these fine details can be worthwhile. In order to resolve some of the more serious problems it may be necessary to carry out additional measurements or structure refinements. However, the purpose of your study may justify the reported deviations and the more serious of these should normally be commented upon in the discussion or experimental section of a paper or in the "special_details" fields of the CIF. checkCIF was carefully designed to identify outliers and unusual parameters, but every test has its limitations and alerts that are not important in a particular case may appear. Conversely, the absence of alerts does not guarantee there are no aspects of the results needing attention. It is up to the individual to critically assess their own results and, if necessary, seek expert advice.

Publication of your CIF in IUCr journals

A basic structural check has been run on your CIF. These basic checks will be run on all CIFs submitted for publication in IUCr journals (*Acta Crystallographica*, *Journal of Applied Crystallography*, *Journal of Synchrotron Radiation*); however, if you intend to submit to *Acta Crystallographica Section C* or *E* or *IUCrData*, you should make sure that full publication checks are run on the final version of your CIF prior to submission.

Publication of your CIF in other journals

Please refer to the *Notes for Authors* of the relevant journal for any special instructions relating to CIF submission.

PLATON version of 15/07/2024; check.def file version of 15/07/2024

Datablock full - ellipsoid plot

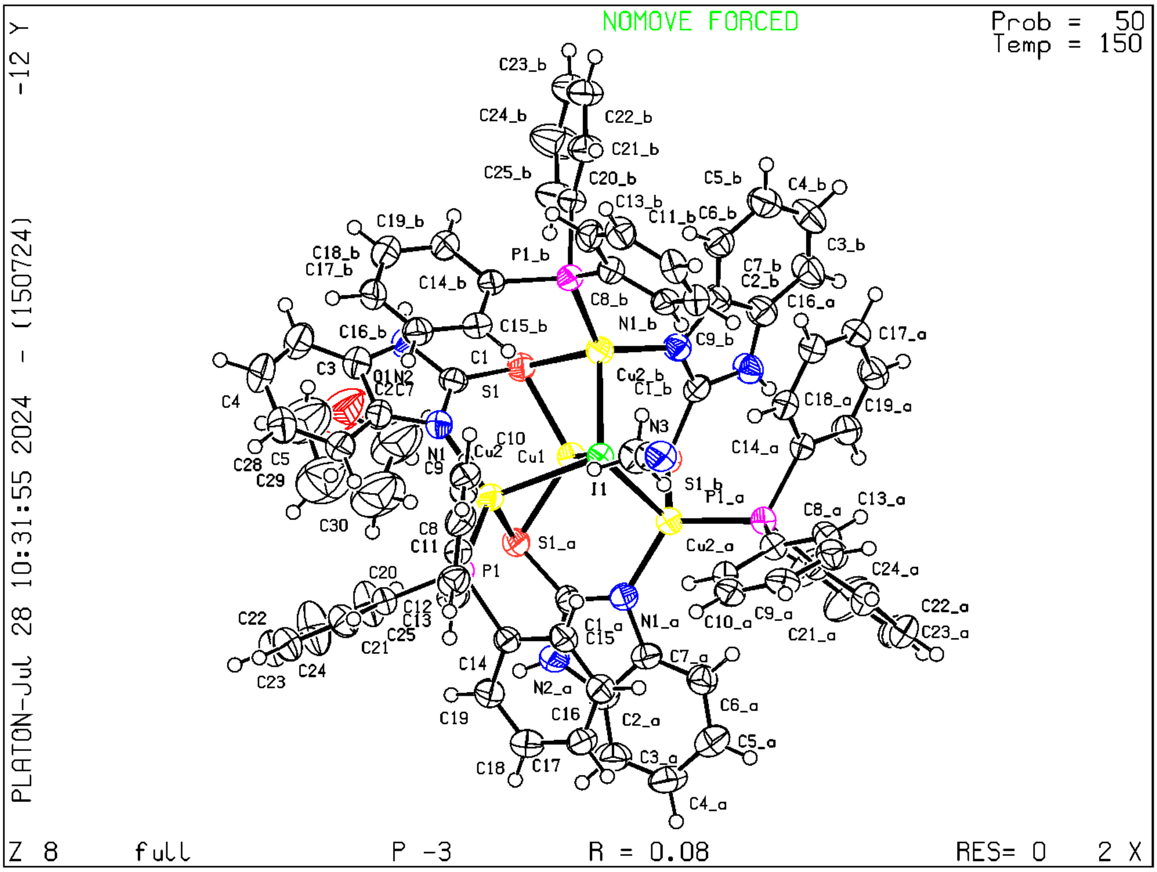

checkCIF/PLATON report

Structure factors have been supplied for datablock(s) full

THIS REPORT IS FOR GUIDANCE ONLY. IF USED AS PART OF A REVIEW PROCEDURE FOR PUBLICATION, IT SHOULD NOT REPLACE THE EXPERTISE OF AN EXPERIENCED CRYSTALLOGRAPHIC REFEREE.

No syntax errors found. CIF dictionary Interpreting this report

Datablock: full Complex 4'

Bond precision: C-C = 0.0076 A Wavelength=1.54184

Cell:	a=16.4700(3)	b=17.0044(2)	c=28.5015(4)
	alpha=81.4516(11)	beta=74.1699(13)	gamma=61.1106(16)
Temperature:	150 K		

	Calculated	Reported
Volume	6722.5(2)	6722.4(2)
Space group	P -1	P -1
Hall group	-P 1	-P 1
Moiety formula	2(C126 H120 Cu4 N12 P4 S6), 7.1(B F4), 2(B0.25 F1.05), 2(B0.20	C126 H120 Cu4 N12 P4 S6, 4(B F4), C2 H3 N, 1.5(H2 O)
Sum formula	C256 H252 B8 Cu8 F32 N26 O3 P8 S12	C128 H126 B4 Cu4 F16 N13 O1.50 P4 S6
Mr	5576.21	2788.05
Dx,g cm-3	1.377	1.377
Z	1	2
Mu (mm-1)	2.667	2.667
F000	2866.0	2866.0
F000'	2864.19	
h,k,lmax	20,20,34	20,20,34
Nref	25498	25399
Tmin,Tmax	0.650,0.766	0.449,1.000
Tmin'	0.559	

Correction method= # Reported T Limits: Tmin=0.449 Tmax=1.000
AbsCorr = MULTI-SCAN

Data completeness= 0.996 Theta(max)= 69.998

R(reflections)= 0.0672(21103) wR2(reflections)= 0.2205(25399)

S = 1.060 Npar= 2058

The following ALERTS were generated. Each ALERT has the format
test-name_ALERT_alert-type_alert-level.
Click on the hyperlinks for more details of the test.

Alert level C

```
DIFMX02_ALERT_1_C  The maximum difference density is > 0.1*ZMAX*0.75
            The relevant atom site should be identified.
PLAT042_ALERT_1_C Calc. and Reported MoietyFormula Strings  Differ     Please Check
              Calc: 2(C126 H120 Cu4 N12 P4 S6), 7.1(B F4), 2(B0.25 F1.05), 2(B0.
              Rep.: C126 H120 Cu4 N12 P4 S6, 4(B F4), C2 H3 N, 1.5
                    (H2 O)
```

Author Response: The structure was solved by a poor-quality diffraction data. Many atoms were disordered into two or three positions. Their bond lengths and bond angles were constrained using DFIX, accompanying with the constraint of the temperature factors by DELU/SIMU.

```
PLAT094_ALERT_2_C Ratio of Maximum / Minimum Residual Density ....       2.21 Report
PLAT097_ALERT_2_C Large Reported Max.  (Positive) Residual Density       2.45 eA-3
```

Author Response: The structure was solved by a poor-quality diffraction data. The max residual density 2.45 eA-3 is located near to copper center with a distance about 0.91 angstrom. It should not belong to any value atom.

```
PLAT220_ALERT_2_C NonSolvent   Resd 1  C   Ueq(max)/Ueq(min) Range        3.9 Ratio
PLAT222_ALERT_3_C NonSolvent Resd 1  H   Uiso(max)/Uiso(min) Range        4.7 Ratio
PLAT241_ALERT_2_C High   'MainMol' Ueq as Compared to Neighbors of        C52 Check
PLAT241_ALERT_2_C High   'MainMol' Ueq as Compared to Neighbors of       C125 Check
PLAT242_ALERT_2_C Low    'MainMol' Ueq as Compared to Neighbors of        C50 Check
PLAT242_ALERT_2_C Low    'MainMol' Ueq as Compared to Neighbors of        C53 Check
PLAT242_ALERT_2_C Low    'MainMol' Ueq as Compared to Neighbors of        C60 Check
PLAT260_ALERT_2_C Large Average Ueq of Residue Including      F13      0.106 Check
PLAT260_ALERT_2_C Large Average Ueq of Residue Including     F13'      0.129 Check
PLAT260_ALERT_2_C Large Average Ueq of Residue Including      N13      0.104 Check
PLAT260_ALERT_2_C Large Average Ueq of Residue Including       O2      0.146 Check
PLAT341_ALERT_3_C Low Bond Precision on  C-C Bonds ...............    0.00762 Ang.
PLAT906_ALERT_3_C Large K Value in the Analysis of Variance ......      2.343 Check
PLAT911_ALERT_3_C Missing FCF Refl Between Thmin & STh/L=    0.600        32 Report
                1  1  1,  17  1  1,  -2 -2  2,  17  0  2, -16  1  2,  -7 13  2,
                4  2  3,  -6 13  4,   0 -2  5,  -1  1  5,  -6 13  5,  -5 14  5,
               -6 13  6,  -5 14  6,  -4 15  6,  -6 13  7,  -5 14  7,  -6 13  8,
               -9  8 14,  -9  7 15,  -7 10 15,  -9  7 16,  -8  8 16,  -8  8 17,
               -7  9 17,  -6  4 26,  -5  4 27,   6 14 27,  -5  3 28,  -4  3 29,
               -3  3 30,  -2  3 31,
PLAT918_ALERT_3_C Reflection(s) with I(obs) much Smaller I(calc) .          5 Check
PLAT971_ALERT_2_C Check Calcd Resid. Dens.  0.94Ang From Cu1             2.37 eA-3
```

```
PLAT971_ALERT_2_C Check Calcd Resid. Dens.  0.15Ang From F5'           1.85 eA-3
PLAT971_ALERT_2_C Check Calcd Resid. Dens.  0.20Ang From F6'           1.61 eA-3
PLAT975_ALERT_2_C Check Calcd Resid. Dens.  0.80Ang From O2      .     1.14 eA-3
PLAT977_ALERT_2_C Check Negative Difference Density on H1A       .    -0.41 eA-3
```

Alert level G

```
PLAT002_ALERT_2_G Number of Distance or Angle Restraints on AtSite         53 Note
PLAT003_ALERT_2_G Number of Uiso or U(i,j) Restrained non-H Atoms         103 Report
PLAT007_ALERT_5_G Number of Unrefined Donor-H Atoms .............          22 Report
              H1    H2    H3    H4    H5    H6    H7'   H8'   H9    H10D  H11H
              H12H  H7    H8    H9'   H10'  H11I  H12I  H1A   H1B   H2A   H2B
PLAT045_ALERT_1_G Calculated and Reported Z Differ by a Factor ...      0.500 Check
PLAT083_ALERT_2_G SHELXL Second Parameter in WGHT  Unusually Large       7.63 Why ?
PLAT172_ALERT_4_G The CIF-Embedded .res File Contains DFIX Records         16 Report
PLAT176_ALERT_4_G The CIF-Embedded .res File Contains SADI Records         16 Report
PLAT177_ALERT_4_G The CIF-Embedded .res File Contains DELU Records          4 Report
PLAT178_ALERT_4_G The CIF-Embedded .res File Contains SIMU Records          4 Report
PLAT186_ALERT_4_G The CIF-Embedded .res File Contains ISOR Records          3 Report
PLAT188_ALERT_3_G A Non-default SIMU Restraint Value has been used     0.0050 Report
PLAT188_ALERT_3_G A Non-default SIMU Restraint Value has been used     0.0050 Report
PLAT188_ALERT_3_G A Non-default SIMU Restraint Value has been used     0.0050 Report
PLAT188_ALERT_3_G A Non-default SIMU Restraint Value has been used     0.0080 Report
PLAT191_ALERT_3_G A Non-default SADI Restraint Value has been used     0.0400 Report
PLAT191_ALERT_3_G A Non-default SADI Restraint Value has been used     0.0400 Report
PLAT191_ALERT_3_G A Non-default SADI Restraint Value has been used     0.0400 Report
PLAT191_ALERT_3_G A Non-default SADI Restraint Value has been used     0.0400 Report
PLAT191_ALERT_3_G A Non-default SADI Restraint Value has been used     0.0400 Report
PLAT191_ALERT_3_G A Non-default SADI Restraint Value has been used     0.0400 Report
PLAT191_ALERT_3_G A Non-default SADI Restraint Value has been used     0.0400 Report
PLAT191_ALERT_3_G A Non-default SADI Restraint Value has been used     0.0400 Report
PLAT192_ALERT_3_G A Non-default DELU Restraint Value for First Par     0.0050 Report
PLAT192_ALERT_3_G A Non-default DELU Restraint Value for SecondPar     0.0050 Report
PLAT192_ALERT_3_G A Non-default DELU Restraint Value for First Par     0.0050 Report
PLAT192_ALERT_3_G A Non-default DELU Restraint Value for SecondPar     0.0050 Report
PLAT192_ALERT_3_G A Non-default DELU Restraint Value for First Par     0.0050 Report
PLAT192_ALERT_3_G A Non-default DELU Restraint Value for SecondPar     0.0050 Report
PLAT192_ALERT_3_G A Non-default DELU Restraint Value for First Par     0.0080 Report
PLAT192_ALERT_3_G A Non-default DELU Restraint Value for SecondPar     0.0080 Report
PLAT232_ALERT_2_G Hirshfeld Test Diff (M-X)  Cu1      --S1       .      24.8 s.u.
PLAT232_ALERT_2_G Hirshfeld Test Diff (M-X)  Cu1      --S2       .       6.8 s.u.
PLAT232_ALERT_2_G Hirshfeld Test Diff (M-X)  Cu1      --S3       .      17.6 s.u.
PLAT232_ALERT_2_G Hirshfeld Test Diff (M-X)  Cu2      --S4       .       8.0 s.u.
PLAT232_ALERT_2_G Hirshfeld Test Diff (M-X)  Cu2      --S6       .       5.6 s.u.
PLAT232_ALERT_2_G Hirshfeld Test Diff (M-X)  Cu3      --S5       .       6.8 s.u.
PLAT232_ALERT_2_G Hirshfeld Test Diff (M-X)  Cu4      --S3       .       9.0 s.u.
PLAT232_ALERT_2_G Hirshfeld Test Diff (M-X)  Cu4      --S5       .       9.6 s.u.
PLAT232_ALERT_2_G Hirshfeld Test Diff (M-X)  Cu4      --S6       .       6.2 s.u.
PLAT299_ALERT_4_G Atom Site Occupancy Constrained at ............         0.5 Check
              O2      H2A     H2B
PLAT300_ALERT_4_G Atom Site Occupancy of F5         Constrained at       0.55 Check
PLAT300_ALERT_4_G Atom Site Occupancy of F6         Constrained at       0.55 Check
PLAT300_ALERT_4_G Atom Site Occupancy of F7         Constrained at       0.55 Check
PLAT300_ALERT_4_G Atom Site Occupancy of F8         Constrained at       0.55 Check
PLAT300_ALERT_4_G Atom Site Occupancy of B2         Constrained at       0.55 Check
PLAT300_ALERT_4_G Atom Site Occupancy of F5'        Constrained at       0.25 Check
PLAT300_ALERT_4_G Atom Site Occupancy of F6'        Constrained at        0.3 Check
```

```
PLAT300_ALERT_4_G Atom Site Occupancy of F7'        Constrained at       0.25 Check
PLAT300_ALERT_4_G Atom Site Occupancy of F8'        Constrained at       0.25 Check
PLAT300_ALERT_4_G Atom Site Occupancy of B2'        Constrained at       0.25 Check
PLAT300_ALERT_4_G Atom Site Occupancy of F5"        Constrained at        0.2 Check
PLAT300_ALERT_4_G Atom Site Occupancy of F6"        Constrained at       0.15 Check
PLAT300_ALERT_4_G Atom Site Occupancy of F7"        Constrained at        0.2 Check
PLAT300_ALERT_4_G Atom Site Occupancy of F8"        Constrained at        0.2 Check
PLAT300_ALERT_4_G Atom Site Occupancy of B2"        Constrained at        0.2 Check
PLAT301_ALERT_3_G Main Residue  Disorder .............(Resd    1)         18% Note
PLAT302_ALERT_4_G Anion/Solvent/Minor-Residue Disorder (Resd    2)        100% Note
PLAT302_ALERT_4_G Anion/Solvent/Minor-Residue Disorder (Resd    3)        100% Note
PLAT302_ALERT_4_G Anion/Solvent/Minor-Residue Disorder (Resd    4)        100% Note
PLAT302_ALERT_4_G Anion/Solvent/Minor-Residue Disorder (Resd    5)        100% Note
PLAT302_ALERT_4_G Anion/Solvent/Minor-Residue Disorder (Resd    6)        100% Note
PLAT302_ALERT_4_G Anion/Solvent/Minor-Residue Disorder (Resd    7)        100% Note
PLAT302_ALERT_4_G Anion/Solvent/Minor-Residue Disorder (Resd    8)        100% Note
PLAT302_ALERT_4_G Anion/Solvent/Minor-Residue Disorder (Resd    9)        100% Note
PLAT302_ALERT_4_G Anion/Solvent/Minor-Residue Disorder (Resd   10)        100% Note
PLAT302_ALERT_4_G Anion/Solvent/Minor-Residue Disorder (Resd   13)        100% Note
PLAT304_ALERT_4_G Non-Integer Number of Atoms in ..... (Resd    2)       3.67 Check
PLAT304_ALERT_4_G Non-Integer Number of Atoms in ..... (Resd    3)       2.75 Check
PLAT304_ALERT_4_G Non-Integer Number of Atoms in ..... (Resd    4)       2.54 Check
PLAT304_ALERT_4_G Non-Integer Number of Atoms in ..... (Resd    5)       3.42 Check
PLAT304_ALERT_4_G Non-Integer Number of Atoms in ..... (Resd    6)       1.33 Check
PLAT304_ALERT_4_G Non-Integer Number of Atoms in ..... (Resd    7)       1.30 Check
PLAT304_ALERT_4_G Non-Integer Number of Atoms in ..... (Resd    8)       0.95 Check
PLAT304_ALERT_4_G Non-Integer Number of Atoms in ..... (Resd    9)       2.46 Check
PLAT304_ALERT_4_G Non-Integer Number of Atoms in ..... (Resd   10)       1.58 Check
PLAT304_ALERT_4_G Non-Integer Number of Atoms in ..... (Resd   13)       1.50 Check
PLAT417_ALERT_2_G Short Inter D-H..H-D       H1A      ..H9       .       1.00 Ang.
                                                       x,y,z  =      1_555 Check
PLAT417_ALERT_2_G Short Inter D-H..H-D       H1A      ..H9'      .       1.28 Ang.
                                                       x,y,z  =      1_555 Check
PLAT432_ALERT_2_G Short Inter X...Y Contact  F3'      ..C6       .       2.97 Ang.
                                                       x,y,z  =      1_555 Check
PLAT790_ALERT_4_G Centre of Gravity not Within Unit Cell: Resd.  #          2 Note
              B F4
PLAT790_ALERT_4_G Centre of Gravity not Within Unit Cell: Resd.  #          6 Note
              B F4
PLAT790_ALERT_4_G Centre of Gravity not Within Unit Cell: Resd.  #         10 Note
              B F4
PLAT790_ALERT_4_G Centre of Gravity not Within Unit Cell: Resd.  #         11 Note
              C2 H3 N
PLAT790_ALERT_4_G Centre of Gravity not Within Unit Cell: Resd.  #         13 Note
              H2 O
PLAT802_ALERT_4_G CIF Input Record(s) with more than 80 Characters          1 Info
PLAT811_ALERT_5_G No ADDSYM Analysis: Too Many Excluded Atoms ....          ! Info
PLAT860_ALERT_3_G Number of Least-Squares Restraints ............        2182 Note
PLAT883_ALERT_1_G No Info/Value for _atom_sites_solution_primary .     Please Do !
PLAT899_ALERT_4_G SHELXL2018 is Deprecated and Succeeded by SHELXL     2019/3 Note
PLAT910_ALERT_3_G Missing # of FCF Reflection(s) Below Theta(Min).          1 Note
                0  0  1,
PLAT912_ALERT_4_G Missing # of FCF Reflections Above STh/L=  0.600         66 Note
PLAT933_ALERT_2_G Number of HKL-OMIT Records in Embedded .res File          5 Note
               -2 -2  2,  -1  1  5,   0 -2  5,   1  1  1,   4  2  3,
PLAT941_ALERT_3_G Average HKL Measurement Multiplicity ..........         4.5 Low
PLAT961_ALERT_5_G Dataset Contains no Negative Intensities ......     Please Check
```

```
PLAT967_ALERT_5_G Note: Two-Theta Cutoff Value in Embedded .res ..      140.0 Degree
PLAT969_ALERT_5_G The 'Henn et al.' R-Factor-gap value ..........      5.044 Note
              Predicted wR2: Based on SigI**2  4.37 or SHELX Weight 20.81
PLAT978_ALERT_2_G Number C-C Bonds with Positive Residual Density.          0 Info
```

```
   0 ALERT level A = Most likely a serious problem - resolve or explain
   0 ALERT level B = A potentially serious problem, consider carefully
  24 ALERT level C = Check. Ensure it is not caused by an omission or oversight
  97 ALERT level G = General information/check it is not something unexpected

   4 ALERT type 1 CIF construction/syntax error, inconsistent or missing data
  34 ALERT type 2 Indicator that the structure model may be wrong or deficient
  29 ALERT type 3 Indicator that the structure quality may be low
  49 ALERT type 4 Improvement, methodology, query or suggestion
   5 ALERT type 5 Informative message, check
```

It is advisable to attempt to resolve as many as possible of the alerts in all categories. Often the minor alerts point to easily fixed oversights, errors and omissions in your CIF or refinement strategy, so attention to these fine details can be worthwhile. In order to resolve some of the more serious problems it may be necessary to carry out additional measurements or structure refinements. However, the purpose of your study may justify the reported deviations and the more serious of these should normally be commented upon in the discussion or experimental section of a paper or in the "special_details" fields of the CIF. checkCIF was carefully designed to identify outliers and unusual parameters, but every test has its limitations and alerts that are not important in a particular case may appear. Conversely, the absence of alerts does not guarantee there are no aspects of the results needing attention. It is up to the individual to critically assess their own results and, if necessary, seek expert advice.

Publication of your CIF in IUCr journals

A basic structural check has been run on your CIF. These basic checks will be run on all CIFs submitted for publication in IUCr journals (*Acta Crystallographica*, *Journal of Applied Crystallography*, *Journal of Synchrotron Radiation*); however, if you intend to submit to *Acta Crystallographica Section C* or *E* or *IUCrData*, you should make sure that full publication checks are run on the final version of your CIF prior to submission.

Publication of your CIF in other journals

Please refer to the *Notes for Authors* of the relevant journal for any special instructions relating to CIF submission.

PLATON version of 15/07/2024; check.def file version of 15/07/2024

Datablock full - ellipsoid plot

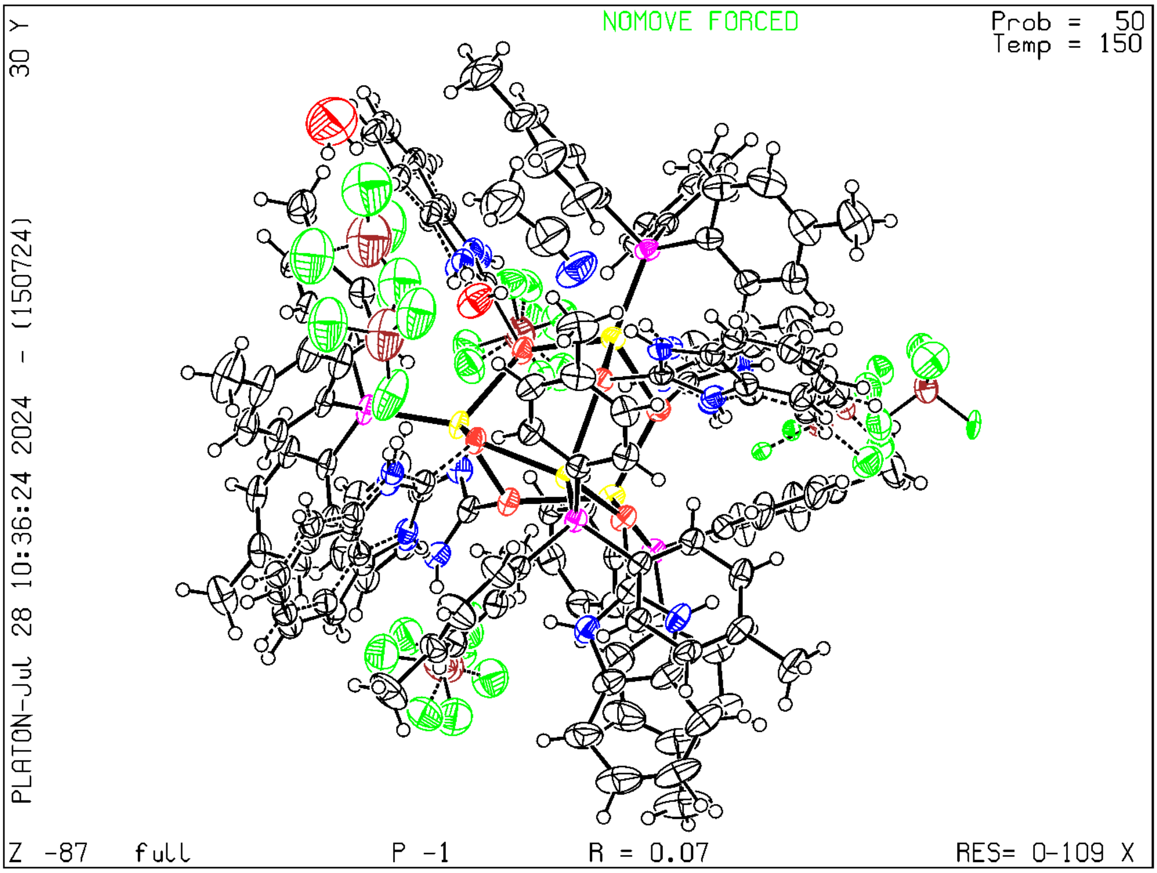

checkCIF/PLATON report

Structure factors have been supplied for datablock(s) full

THIS REPORT IS FOR GUIDANCE ONLY. IF USED AS PART OF A REVIEW PROCEDURE FOR PUBLICATION, IT SHOULD NOT REPLACE THE EXPERTISE OF AN EXPERIENCED CRYSTALLOGRAPHIC REFEREE.

No syntax errors found.   CIF dictionary   Interpreting this report

Datablock: full   Complex 6

```
Bond precision:   C-C = 0.0040 A          Wavelength=1.54184

Cell:          a=14.1645(3)       b=14.6533(3)       c=23.8066(5)
               alpha=81.6263(19)  beta=76.0245(19)   gamma=66.465(2)
Temperature:   150 K
```

	Calculated	Reported
Volume	4389.37(18)	4389.39(18)
Space group	P -1	P -1
Hall group	-P 1	-P 1
Moiety formula	C100 H76 Cu4 N4 O4 P4 S4	C100 H76 Cu4 N4 O4 P4 S4
Sum formula	C100 H76 Cu4 N4 O4 P4 S4	C100 H76 Cu4 N4 O4 P4 S4
Mr	1903.97	1903.92
Dx,g cm-3	1.441	1.441
Z	2	2
Mu (mm-1)	3.109	3.109
F000	1952.0	1952.0
F000’	1945.30	
h,k,lmax	17,17,29	17,17,29
Nref	16637	16607
Tmin,Tmax	0.742,0.856	0.753,1.000
Tmin’	0.733	

```
Correction method= # Reported T Limits: Tmin=0.753 Tmax=1.000
AbsCorr = MULTI-SCAN

Data completeness= 0.998          Theta(max)= 69.992

R(reflections)= 0.0335( 14655)          wR2(reflections)= 0.0913( 16607)

S = 1.060                 Npar= 1223
```

The following ALERTS were generated. Each ALERT has the format
test-name_ALERT_alert-type_alert-level.
Click on the hyperlinks for more details of the test.

Alert level C

```
PLAT213_ALERT_2_C Atom C97'             has ADP max/min Ratio .....        3.1 oblate
PLAT213_ALERT_2_C Atom C95              has ADP max/min Ratio .....        3.5 oblate
PLAT220_ALERT_2_C NonSolvent   Resd 1  C   Ueq(max)/Ueq(min) Range         3.3 Ratio
PLAT601_ALERT_2_C Unit Cell Contains Solvent Accessible VOIDS of .          38 Ang**3
```

Alert level G

```
PLAT002_ALERT_2_G Number of Distance or Angle Restraints on AtSite           2 Note
PLAT003_ALERT_2_G Number of Uiso or U(i,j) Restrained non-H Atoms          146 Report
PLAT172_ALERT_4_G The CIF-Embedded .res File Contains DFIX Records           1 Report
PLAT174_ALERT_4_G The CIF-Embedded .res File Contains FLAT Records           1 Report
PLAT177_ALERT_4_G The CIF-Embedded .res File Contains DELU Records           1 Report
PLAT178_ALERT_4_G The CIF-Embedded .res File Contains SIMU Records           1 Report
PLAT188_ALERT_3_G A Non-default SIMU Restraint Value has been used      0.0050 Report
PLAT192_ALERT_3_G A Non-default DELU Restraint Value for First Par      0.0050 Report
PLAT192_ALERT_3_G A Non-default DELU Restraint Value for SecondPar      0.0050 Report
PLAT230_ALERT_2_G Hirshfeld Test Diff for    O4'      --C94      .         5.7 s.u.
PLAT230_ALERT_2_G Hirshfeld Test Diff for    N4'      --C94      .         5.5 s.u.
PLAT230_ALERT_2_G Hirshfeld Test Diff for    N4'      --C95'     .         6.0 s.u.
PLAT232_ALERT_2_G Hirshfeld Test Diff (M-X)  Cu1      --S4       .         5.3 s.u.
PLAT232_ALERT_2_G Hirshfeld Test Diff (M-X)  Cu2      --S1       .         8.0 s.u.
PLAT232_ALERT_2_G Hirshfeld Test Diff (M-X)  Cu2      --S2       .        10.0 s.u.
PLAT232_ALERT_2_G Hirshfeld Test Diff (M-X)  Cu3      --S2       .         7.3 s.u.
PLAT232_ALERT_2_G Hirshfeld Test Diff (M-X)  Cu3      --S3       .         6.3 s.u.
PLAT232_ALERT_2_G Hirshfeld Test Diff (M-X)  Cu4      --S4       .         6.3 s.u.
PLAT301_ALERT_3_G Main Residue  Disorder .............(Resd    1)          22% Note
PLAT398_ALERT_2_G Deviating  C-O-C   Angle From 120 for O1       .       105.2 Degree
PLAT398_ALERT_2_G Deviating  C-O-C   Angle From 120 for O2       .       104.6 Degree
PLAT398_ALERT_2_G Deviating  C-O-C   Angle From 120 for O3       .       105.0 Degree
PLAT398_ALERT_2_G Deviating  C-O-C   Angle From 120 for O4'      .       104.4 Degree
PLAT398_ALERT_2_G Deviating  C-O-C   Angle From 120 for O4       .       102.0 Degree
PLAT720_ALERT_4_G Number of Unusual/Non-Standard Labels .........            2 Note
              C00     C00'
PLAT860_ALERT_3_G Number of Least-Squares Restraints ............         1882 Note
PLAT883_ALERT_1_G No Info/Value for _atom_sites_solution_primary .     Please Do !
PLAT899_ALERT_4_G SHELXL2018 is Deprecated and Succeeded by SHELXL      2019/3 Note
PLAT910_ALERT_3_G Missing # of FCF Reflection(s) Below Theta(Min).           2 Note
                0  1  0,   0  0  1,
PLAT912_ALERT_4_G Missing # of FCF Reflections Above STh/L=  0.600          27 Note
PLAT941_ALERT_3_G Average HKL Measurement Multiplicity ..........          4.9 Low
PLAT961_ALERT_5_G Dataset Contains no Negative Intensities ......     Please Check
PLAT967_ALERT_5_G Note: Two-Theta Cutoff Value in Embedded .res ..       140.0 Degree
PLAT969_ALERT_5_G The 'Henn et al.' R-Factor-gap value ..........        3.691 Note
              Predicted wR2: Based on SigI**2  2.47 or SHELX Weight  8.61
PLAT978_ALERT_2_G Number C-C Bonds with Positive Residual Density.          13 Info
```

0 **ALERT level A** = Most likely a serious problem - resolve or explain
0 **ALERT level B** = A potentially serious problem, consider carefully
4 **ALERT level C** = Check. Ensure it is not caused by an omission or oversight

```
35 ALERT level G = General information/check it is not something unexpected

 1 ALERT type 1 CIF construction/syntax error, inconsistent or missing data
21 ALERT type 2 Indicator that the structure model may be wrong or deficient
 7 ALERT type 3 Indicator that the structure quality may be low
 7 ALERT type 4 Improvement, methodology, query or suggestion
 3 ALERT type 5 Informative message, check
```

It is advisable to attempt to resolve as many as possible of the alerts in all categories. Often the minor alerts point to easily fixed oversights, errors and omissions in your CIF or refinement strategy, so attention to these fine details can be worthwhile. In order to resolve some of the more serious problems it may be necessary to carry out additional measurements or structure refinements. However, the purpose of your study may justify the reported deviations and the more serious of these should normally be commented upon in the discussion or experimental section of a paper or in the "special_details" fields of the CIF. checkCIF was carefully designed to identify outliers and unusual parameters, but every test has its limitations and alerts that are not important in a particular case may appear. Conversely, the absence of alerts does not guarantee there are no aspects of the results needing attention. It is up to the individual to critically assess their own results and, if necessary, seek expert advice.

Publication of your CIF in IUCr journals

A basic structural check has been run on your CIF. These basic checks will be run on all CIFs submitted for publication in IUCr journals (*Acta Crystallographica*, *Journal of Applied Crystallography*, *Journal of Synchrotron Radiation*); however, if you intend to submit to *Acta Crystallographica Section C* or *E* or *IUCrData*, you should make sure that full publication checks are run on the final version of your CIF prior to submission.

Publication of your CIF in other journals

Please refer to the *Notes for Authors* of the relevant journal for any special instructions relating to CIF submission.

PLATON version of 15/07/2024; check.def file version of 15/07/2024

Datablock full - ellipsoid plot

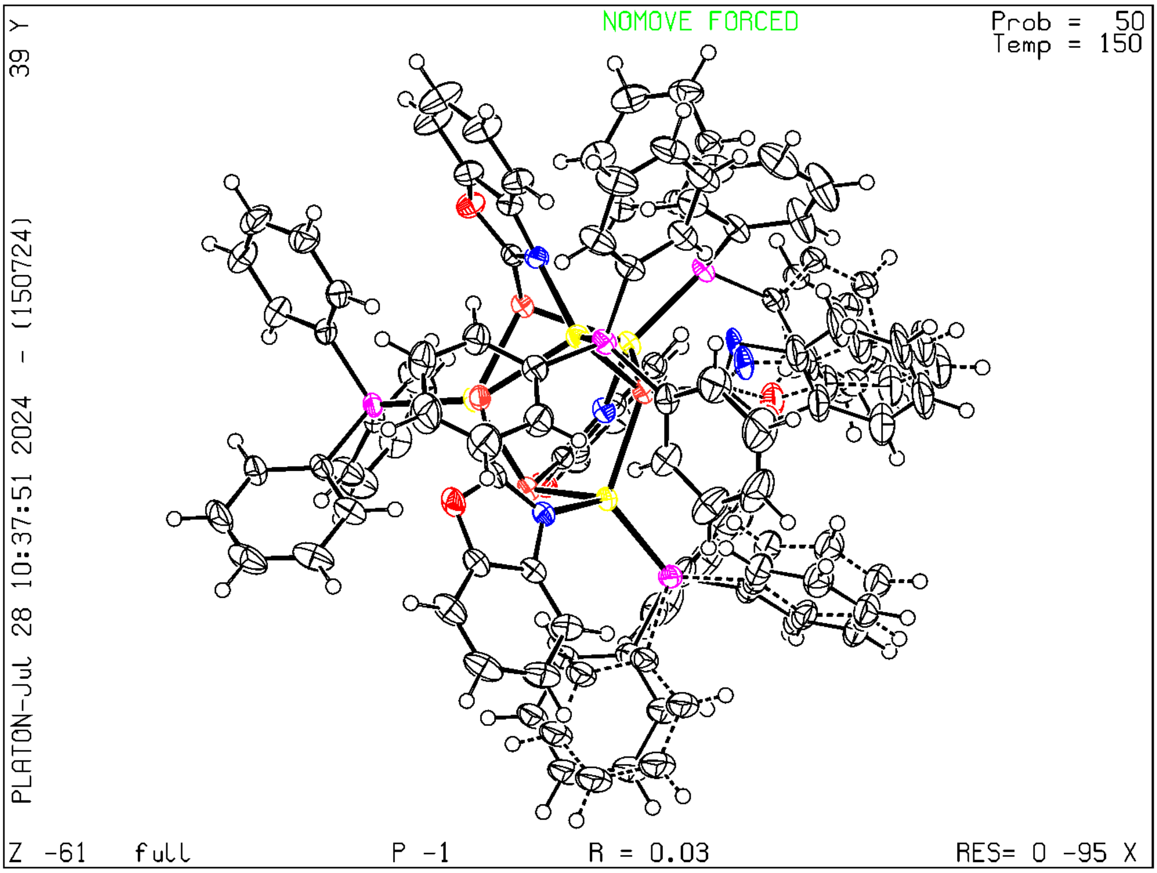

checkCIF/PLATON report

Structure factors have been supplied for datablock(s) full

THIS REPORT IS FOR GUIDANCE ONLY. IF USED AS PART OF A REVIEW PROCEDURE FOR PUBLICATION, IT SHOULD NOT REPLACE THE EXPERTISE OF AN EXPERIENCED CRYSTALLOGRAPHIC REFEREE.

No syntax errors found. CIF dictionary Interpreting this report

Datablock: full Complex 7

Bond precision: C-C = 0.0093 A Wavelength=1.54184

Cell:	a=15.8043(2)	b=22.2664(4)	c=21.4443(3)
	alpha=90	beta=101.3164(15)	gamma=90
Temperature:	150 K		

	Calculated	Reported
Volume	7399.6(2)	7399.7(2)
Space group	P 21/n	P 21/n
Hall group	-P 2yn	-P 2yn
Moiety formula	C75 H57 Cu4 I N3 O3 P3 S3, C H Cl3	C76 H58 Cl3 Cu4 I N3 O3 P3 S3
Sum formula	C76 H58 Cl3 Cu4 I N3 O3 P3 S3	C76 H58 Cl3 Cu4 I N3 O3 P3 S3
Mr	1737.80	1737.75
Dx,g cm-3	1.560	1.560
Z	4	4
Mu (mm-1)	7.360	7.360
F000	3488.0	3488.0
F000'	3473.61	
h,k,lmax	19,27,26	19,27,26
Nref	14028	13933
Tmin,Tmax	0.381,0.479	0.671,1.000
Tmin'	0.288	

Correction method= # Reported T Limits: Tmin=0.671 Tmax=1.000
AbsCorr = MULTI-SCAN

Data completeness= 0.993 Theta(max)= 69.990

R(reflections)= 0.0586(11455) wR2(reflections)= 0.1606(13933)

S = 1.035 Npar= 902

The following ALERTS were generated. Each ALERT has the format
test-name_ALERT_alert-type_alert-level.
Click on the hyperlinks for more details of the test.

Alert level B

```
PLAT601_ALERT_2_B Unit Cell Contains Solvent Accessible VOIDS of .        104 Ang**3
```

Author Response: No residual density could be found suitable for any solvent. The highest four Q peaks are near around the heavy anion I- with the residual density bewteen 1.51-1.67. The density of the fifth and more peaks are below 1.0, which would not belong to any solvent or anions.

Alert level C

```
PLAT042_ALERT_1_C Calc. and Reported MoietyFormula Strings  Differ     Please Check
              Calc: C75 H57 Cu4 I N3 O3 P3 S3, C H Cl3
              Rep.: C76 H58 Cl3 Cu4 I N3 O3 P3 S3
PLAT260_ALERT_2_C Large Average Ueq of Residue Including       Cl1      0.103 Check
PLAT342_ALERT_3_C Low Bond Precision on  C-C Bonds ...............    0.00926 Ang.
PLAT906_ALERT_3_C Large K Value in the Analysis of Variance ......      3.456 Check
PLAT911_ALERT_3_C Missing FCF Refl Between Thmin & STh/L=    0.600         26 Report
               -3  3  2, -18  8  3, -18  8  4,  -3 11  4, -18  8  5, -17 11  5,
              -18  6  7, -18  3  8, -16 12  8, -18  1  9, -18  2  9, -17  2  9,
              -17  3  9, -17  4  9, -16  5  9, -17  2 10, -17  3 10, -17  6 11,
               -4 22 14,  -6 21 15,  -2 10 16,  -2 11 16,   1  6 20,  -7  8 24,
               -5  9 24,  -4  9 24,
PLAT934_ALERT_3_C Number of (Iobs-Icalc)/Sigma(W) > 10 Outliers ..          1 Check
              -18  4  8,
PLAT971_ALERT_2_C Check Calcd Resid. Dens.  1.01Ang From I1           1.92 eA-3
PLAT971_ALERT_2_C Check Calcd Resid. Dens.  1.02Ang From I1           1.90 eA-3
PLAT971_ALERT_2_C Check Calcd Resid. Dens.  1.06Ang From I1           1.72 eA-3
PLAT971_ALERT_2_C Check Calcd Resid. Dens.  1.03Ang From I1           1.65 eA-3
PLAT972_ALERT_2_C Check Calcd Resid. Dens.  0.79Ang From I1          -1.63 eA-3
```

Alert level G

```
PLAT002_ALERT_2_G Number of Distance or Angle Restraints on AtSite          4 Note
PLAT003_ALERT_2_G Number of Uiso or U(i,j) Restrained non-H Atoms           8 Report
PLAT083_ALERT_2_G SHELXL Second Parameter in WGHT  Unusually Large      34.02 Why ?
PLAT172_ALERT_4_G The CIF-Embedded .res File Contains DFIX Records          1 Report
PLAT176_ALERT_4_G The CIF-Embedded .res File Contains SADI Records          2 Report
PLAT177_ALERT_4_G The CIF-Embedded .res File Contains DELU Records          1 Report
PLAT178_ALERT_4_G The CIF-Embedded .res File Contains SIMU Records          1 Report
PLAT188_ALERT_3_G A Non-default SIMU Restraint Value has been used     0.0070 Report
PLAT191_ALERT_3_G A Non-default SADI Restraint Value has been used     0.0400 Report
PLAT192_ALERT_3_G A Non-default DELU Restraint Value for First Par     0.0070 Report
PLAT192_ALERT_3_G A Non-default DELU Restraint Value for SecondPar     0.0070 Report
```

```
PLAT232_ALERT_2_G Hirshfeld Test Diff (M-X)  I1       --Cu1      .       39.5 s.u.
PLAT232_ALERT_2_G Hirshfeld Test Diff (M-X)  I1       --Cu2      .        7.2 s.u.
PLAT232_ALERT_2_G Hirshfeld Test Diff (M-X)  I1       --Cu3      .        8.0 s.u.
PLAT232_ALERT_2_G Hirshfeld Test Diff (M-X)  I1       --Cu4      .       11.5 s.u.
PLAT232_ALERT_2_G Hirshfeld Test Diff (M-X)  Cu3      --S2       .        5.4 s.u.
PLAT302_ALERT_4_G Anion/Solvent/Minor-Residue Disorder (Resd    2)       100% Note
PLAT302_ALERT_4_G Anion/Solvent/Minor-Residue Disorder (Resd    3)       100% Note
PLAT304_ALERT_4_G Non-Integer Number of Atoms in ..... (Resd    2)       4.11 Check
PLAT304_ALERT_4_G Non-Integer Number of Atoms in ..... (Resd    3)       0.90 Check
PLAT398_ALERT_2_G Deviating  C-O-C   Angle From 120 for O1       .      104.8 Degree
PLAT398_ALERT_2_G Deviating  C-O-C   Angle From 120 for O2       .      104.8 Degree
PLAT398_ALERT_2_G Deviating  C-O-C   Angle From 120 for O3       .      105.0 Degree
PLAT860_ALERT_3_G Number of Least-Squares Restraints ............        117 Note
PLAT883_ALERT_1_G No Info/Value for _atom_sites_solution_primary .     Please Do !
PLAT899_ALERT_4_G SHELXL2018 is Deprecated and Succeeded by SHELXL     2019/3 Note
PLAT912_ALERT_4_G Missing # of FCF Reflections Above STh/L=  0.600         69 Note
PLAT933_ALERT_2_G Number of HKL-OMIT Records in Embedded .res File         17 Note
              -19  0  7, -19  2  6, -19  2  7, -19  3  5, -19  3  6, -18  1  9,
              -18  2  9, -18  3  8, -18  6  7, -17  2  9, -17  2 10, -17  3  9,
              -17  3 10, -17  4  9, -17  6 11, -17  6 16, -16  5  9,
PLAT941_ALERT_3_G Average HKL Measurement Multiplicity ..........        2.8 Low
PLAT961_ALERT_5_G Dataset Contains no Negative Intensities .......     Please Check
PLAT965_ALERT_2_G The SHELXL WEIGHT Optimisation has not Converged     Please Check
PLAT967_ALERT_5_G Note: Two-Theta Cutoff Value in Embedded .res ..      140.0 Degree
PLAT969_ALERT_5_G The 'Henn et al.' R-Factor-gap value ..........      3.098 Note
              Predicted wR2: Based on SigI**2  5.18 or SHELX Weight 15.52
PLAT978_ALERT_2_G Number C-C Bonds with Positive Residual Density.          0 Info
```

---

   0 **ALERT level A** = Most likely a serious problem - resolve or explain
   1 **ALERT level B** = A potentially serious problem, consider carefully
  11 **ALERT level C** = Check. Ensure it is not caused by an omission or oversight
  34 **ALERT level G** = General information/check it is not something unexpected

   2 ALERT type 1 CIF construction/syntax error, inconsistent or missing data
  21 ALERT type 2 Indicator that the structure model may be wrong or deficient
  10 ALERT type 3 Indicator that the structure quality may be low
  10 ALERT type 4 Improvement, methodology, query or suggestion
   3 ALERT type 5 Informative message, check

It is advisable to attempt to resolve as many as possible of the alerts in all categories. Often the minor alerts point to easily fixed oversights, errors and omissions in your CIF or refinement strategy, so attention to these fine details can be worthwhile. In order to resolve some of the more serious problems it may be necessary to carry out additional measurements or structure refinements. However, the purpose of your study may justify the reported deviations and the more serious of these should normally be commented upon in the discussion or experimental section of a paper or in the "special_details" fields of the CIF. checkCIF was carefully designed to identify outliers and unusual parameters, but every test has its limitations and alerts that are not important in a particular case may appear. Conversely, the absence of alerts does not guarantee there are no aspects of the results needing attention. It is up to the individual to critically assess their own results and, if necessary, seek expert advice.

Publication of your CIF in IUCr journals

A basic structural check has been run on your CIF. These basic checks will be run on all CIFs submitted for publication in IUCr journals (*Acta Crystallographica*, *Journal of Applied Crystallography*, *Journal of Synchrotron Radiation*); however, if you intend to submit to *Acta Crystallographica Section C* or *E* or *IUCrData*, you should make sure that full publication checks are run on the final version of your CIF prior to submission.

Publication of your CIF in other journals

Please refer to the *Notes for Authors* of the relevant journal for any special instructions relating to CIF submission.

PLATON version of 15/07/2024; check.def file version of 15/07/2024

Datablock full - ellipsoid plot

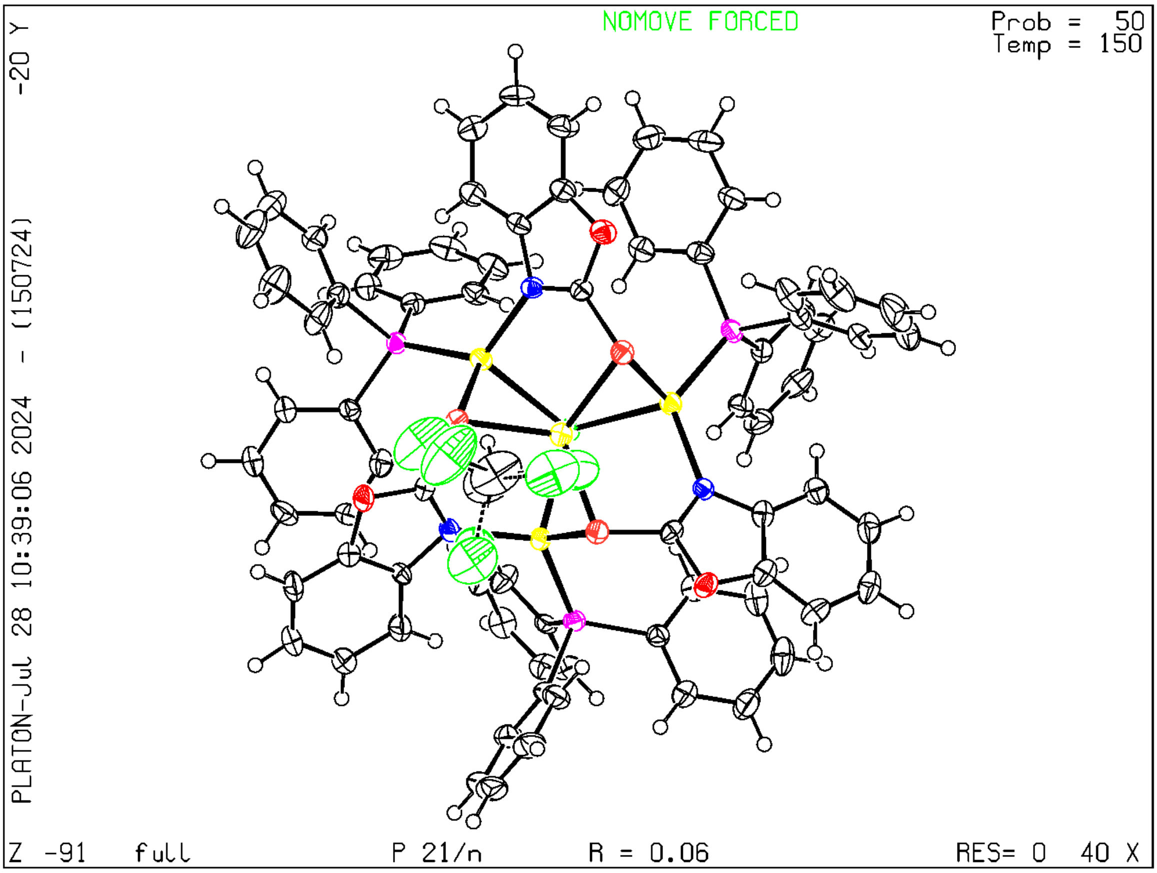

checkCIF/PLATON report

Structure factors have been supplied for datablock(s) full

THIS REPORT IS FOR GUIDANCE ONLY. IF USED AS PART OF A REVIEW PROCEDURE FOR PUBLICATION, IT SHOULD NOT REPLACE THE EXPERTISE OF AN EXPERIENCED CRYSTALLOGRAPHIC REFEREE.

No syntax errors found. CIF dictionary Interpreting this report

Datablock: full Complex 8

Bond precision: C-C = 0.0063 A Wavelength=1.54184

Cell:	a=25.6927(6)	b=9.2577(2)	c=20.2000(4)
	alpha=90	beta=100.058(2)	gamma=90
Temperature:	150 K		

	Calculated	Reported
Volume	4730.84(18)	4730.84(18)
Space group	C 2/c	C 2/c
Hall group	-C 2yc	-C 2yc
Moiety formula	C50 H40 Cu2 I2 N2 P2 S4	C50 H40 Cu2 I2 N2 P2 S4
Sum formula	C50 H40 Cu2 I2 N2 P2 S4	C50 H40 Cu2 I2 N2 P2 S4
Mr	1239.92	1239.90
Dx,g cm-3	1.741	1.741
Z	4	4
Mu (mm-1)	13.938	13.938
F000	2448.0	2448.0
F000'	2442.03	
h,k,lmax	31,11,24	31,11,24
Nref	4490	4445
Tmin,Tmax	0.310,0.328	0.471,1.000
Tmin'	0.199	

Correction method= # Reported T Limits: Tmin=0.471 Tmax=1.000
AbsCorr = MULTI-SCAN

Data completeness= 0.990 Theta(max)= 69.998

R(reflections)= 0.0332(3827) wR2(reflections)= 0.0893(4445)

S = 1.058 Npar= 284

The following ALERTS were generated. Each ALERT has the format
test-name_ALERT_alert-type_alert-level.
Click on the hyperlinks for more details of the test.

Alert level C

```
PLAT911_ALERT_3_C Missing FCF Refl Between Thmin & STh/L=    0.600         15 Report
                1 11  0,   3 11  0,  -3 11  1,  -1 11  1,   1 11  1,   3 11  1,
               -3 11  2,  -1 11  2,   1 11  2, -15  3  3,  -1 11  3,   1 11  3,
              -24  4 11,  12  8 12,  10  8 13,
```

Alert level G

```
PLAT232_ALERT_2_G Hirshfeld Test Diff (M-X)  I1       --Cu1      .        8.7 s.u.
PLAT232_ALERT_2_G Hirshfeld Test Diff (M-X)  Cu1      --S1       .        5.5 s.u.
PLAT232_ALERT_2_G Hirshfeld Test Diff (M-X)  Cu1      --S1_a     .       11.2 s.u.
PLAT883_ALERT_1_G No Info/Value for _atom_sites_solution_primary .     Please Do !
PLAT899_ALERT_4_G SHELXL2018 is Deprecated and Succeeded by SHELXL     2019/3 Note
PLAT910_ALERT_3_G Missing # of FCF Reflection(s) Below Theta(Min).          1 Note
                2  0  0,
PLAT912_ALERT_4_G Missing # of FCF Reflections Above STh/L=  0.600         29 Note
PLAT913_ALERT_3_G Missing # of Very Strong Reflections in FCF ....          1 Note
                2  0  0,
PLAT941_ALERT_3_G Average HKL Measurement Multiplicity ..........        2.7 Low
PLAT961_ALERT_5_G Dataset Contains no Negative Intensities ......     Please Check
PLAT965_ALERT_2_G The SHELXL WEIGHT Optimisation has not Converged     Please Check
PLAT967_ALERT_5_G Note: Two-Theta Cutoff Value in Embedded .res ..      140.0 Degree
PLAT969_ALERT_5_G The 'Henn et al.' R-Factor-gap value ..........      2.494 Note
              Predicted wR2: Based on SigI**2  3.58 or SHELX Weight  8.43
PLAT978_ALERT_2_G Number C-C Bonds with Positive Residual Density.          0 Info
```

0 **ALERT level A** = Most likely a serious problem - resolve or explain
0 **ALERT level B** = A potentially serious problem, consider carefully
1 **ALERT level C** = Check. Ensure it is not caused by an omission or oversight
14 **ALERT level G** = General information/check it is not something unexpected

1 ALERT type 1 CIF construction/syntax error, inconsistent or missing data
5 ALERT type 2 Indicator that the structure model may be wrong or deficient
4 ALERT type 3 Indicator that the structure quality may be low
2 ALERT type 4 Improvement, methodology, query or suggestion
3 ALERT type 5 Informative message, check

It is advisable to attempt to resolve as many as possible of the alerts in all categories. Often the minor alerts point to easily fixed oversights, errors and omissions in your CIF or refinement strategy, so attention to these fine details can be worthwhile. In order to resolve some of the more serious problems it may be necessary to carry out additional measurements or structure refinements. However, the purpose of your study may justify the reported deviations and the more serious of these should normally be commented upon in the discussion or experimental section of a paper or in the "special_details" fields of the CIF. checkCIF was carefully designed to identify outliers and unusual parameters, but every test has its limitations and alerts that are not important in a particular case may appear. Conversely, the absence of alerts does not guarantee there are no aspects of the results needing attention. It is up to the individual to critically assess their own results and, if necessary, seek expert advice.

Publication of your CIF in IUCr journals

A basic structural check has been run on your CIF. These basic checks will be run on all CIFs submitted for publication in IUCr journals (*Acta Crystallographica*, *Journal of Applied Crystallography*, *Journal of Synchrotron Radiation*); however, if you intend to submit to *Acta Crystallographica Section C* or *E* or *IUCrData*, you should make sure that full publication checks are run on the final version of your CIF prior to submission.

Publication of your CIF in other journals

Please refer to the *Notes for Authors* of the relevant journal for any special instructions relating to CIF submission.

PLATON version of 15/07/2024; check.def file version of 15/07/2024

Datablock full - ellipsoid plot

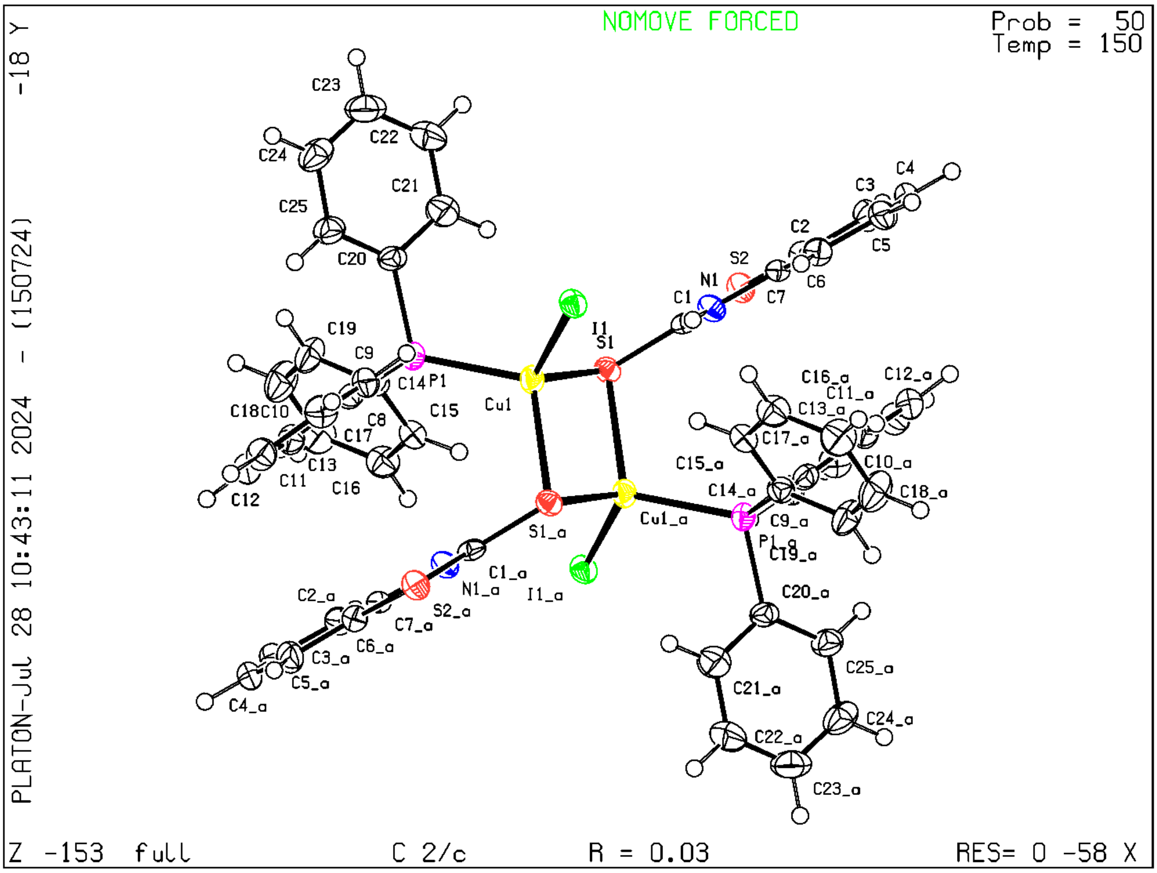

Supplement: Supplementary file 1 [file molecules-29-04228-s001.zip › cifreport.pdf]
