# Supplementary material for: Research on Synthesis, Structure, and Catalytic Performance of Tetranuclear Copper(I) Clusters Supported by 2-Mercaptobenz-zole-Type Ligands
Source: Molecules. 2024 Sep 6;29(17):4228. doi: 10.3390/molecules29174228 (PMC11396812; doi:10.3390/molecules29174228)
Supplement: Supplementary file 1 [file molecules-29-04228-s001.zip › Supporting Information-revised.pdf]

Supporting Information

Research on Synthesis, Structure and Catalytic Performance of Tetranuclear Copper(I) Clusters Supported by 2-Mercaptobenz-zole Type Ligands

Tingyu Zhu[a,b],Wangyuan Zhan[a,b], Weibin Fan[a] and Xiaofeng Zhang[*a]

[a]*State Key Laboratory of Structural Chemistry, Fujian Institute of Research on the Structure of Matter, Chinese Academy of Sciences, Fuzhou, Fujian 350002, China*

[b]*Fujian Normal University, College of Chemistry and Materials Science, Fuzhou 350007, China.*

[c]*Fujian College, University of Chinese Academy of Sciences, Fuzhou, Fujian 350002, China*

**To whom correspondence should be addressed. E-mail: dhuang@fjirsm.ac.cn*

Table of contents:

1. General information··· S2

2. Experimental procedure··· S2

3. XRD patterns of tetranuclear copper(I) clusters··· S3

4. Cyclic voltammetry tests for tetranuclear copper(I) clusters························ S6

5. DSC (differential scanning calorimetry) of complex **3**··································· S8

6. X-ray structure determinations··· S8

7. Crystallographic data of compounds··· S9

8. ^{1}H NMR and ^{13}C NMR spectra of compounds····································· S10

9. References··· S29

1. General Information

Chemicals. Unless otherwise stated, all inorganic reactions and manipulations are performed under Air atmosphere. Volume reduction and drying steps are performed in vacuo. All the reagents are purchased from commercial sources and used as received. *N,N'*-dimethylformamide and acetonitrile are freshly distilled over CaH_2. Tetrahydrofuran and 1,4-dioxiane are distilled over sodium under N_2. Complex [Cu(CH_3CN)$_4$]BF_4, 2,4,6-tris(5-bromothiophen-2-yl)-1,3,5-triazine and (4-(octyloxy)phenyl)boronic acid are prepared as described in the literature.[1-4]

General Physical Measurements. ^{1}H NMR and ^{13}C NMR were recorded on Bruker Avance III (400MHz) and chemical shifts were expressed in δ ppm values with reference to tetramethylsilane (TMS) as internal standard. The single crystal data were collected on an Oxford Diffraction Supernova dual diffractometer equipped with an Oxford Cryostream 700 low-temperature apparatus. UV−vis spectra are recorded with a Lambda365 (190−1100 nm) ultraviolet spectrophotometer. Fluorescence spectra are recorded with a FLS1000 Spectrometer. The XRD spectrum were recorded by X-ray diffractometer MiniFlex 600. Elemental analyses of C, H and N are performed with a Vario EL III CHNOS elemental analyzer. Cyclic voltammetry is carried out in 10^{-3} M solutions of the complexes under an atmosphere of Ar on a CHI630A potentiostat. A single compartmental cell is used with glassy-carbon, Pt net and Ag/AgCl functioning as the working, counter and reference electrodes, respectively. All potentials are quoted versus the ferrocenium-ferrocene couple.[5] Tetrabutylammonium hexafluorophosphate is used as the supporting electrolyte.

2. Experimental procedure

[Cu^I_6(MBIZ)$_6$]·THF (1). 2-mercaptobenzimidazole (MBIZ) (15.00 mg, 0.1 mmol) and Et_3N (10.38 mg, 0.15 mmol) were dissolved in THF (2 mL) stirring for 15 minutes. The solution of MBIZ was added to a solution of [Cu(CH_3CN)]BF_4 (31.50 mg, 0.1 mmol) in MeCN (2 mL) dropwise. The mixture was stirred at 25 ^{o}C for 15 minutes. The solution was filtered and evaporated at room temperature, then deposited as orange crystals in three days (17.26 mg, 76.78%). Anal. Calcd. for $C_{46}H_{38}Cu_6N_{12}S_6O$: C, 40.97; H, 2.84; N, 12.46. Found: C, 41.21; H, 2.85; N, 12.39.

[Cu^I_8(MBOZ)$_8$I](H_3O)·CH_3CN (5). 2-mercaptobenzoxazole (MBOZ) (15.00 mg, 0.1 mmol) and Et_3N (10.38 mg, 0.15 mmol) were dissolved in THF (2 mL) stirring for 15 minutes. The solution of MBOZ was added to the solution of CuI (19.05 mg, 0.1 mmol) in MeCN (2 mL) dropwise. The mixture was stirred at 25 ^{o}C for 15 minutes. The solution was filtered and evaporated at room temperature, then deposited product as colorless crystals in three days (10.25 mg, 44.18%). Anal. Calcd. for $C_{58}H_{38}Cu_8N_9O_9S_8I$: C, 36.73; H, 2.02; N, 6.65. Found: C, 36.38; H, 2.06; N, 6.58.

[Cu^I_2(MBTZ)$_2$(PPh_3)$_2$$I_2$] (8). The solution of CuI (19.05 mg, 0.1 mmol) in MeCN (2 mL) was added to the solution of 2-mercaptobenzothiazole (MBTZ, 16.70 mg, 0.1 mmol) in THF (2 mL) dropwise. The mixture was stirred at 25 ^{o}C for 15 minutes. The resulting light yellow solution was treated with PPh_3 (26.20 mg, 0.1 mmol) and stirred for 15 minutes. The solution was filtered and evaporated at room temperature, then deposited product as light yellow crystals in three days (37.12 mg, 59.86%). Anal. Calcd. for $C_{50}H_{40}Cu_2I_2N_2P_2S_4$: C, 48.43; H, 3.25; N, 2.26. Found: C, 48.91; H, 3.37; N, 2.28.

3. XRD patterns of Tetranuclear Copper(I) Clusters

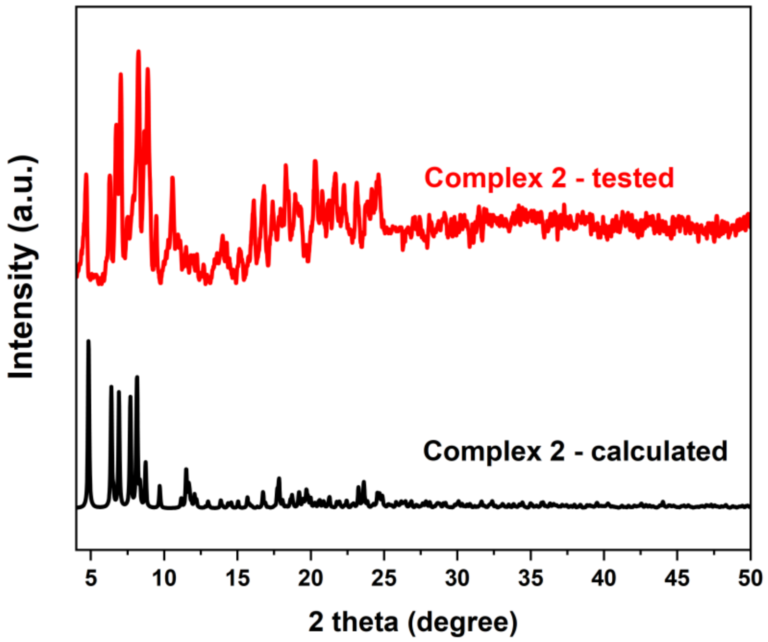

Fig. S1 XRD patterns of complex **2**.

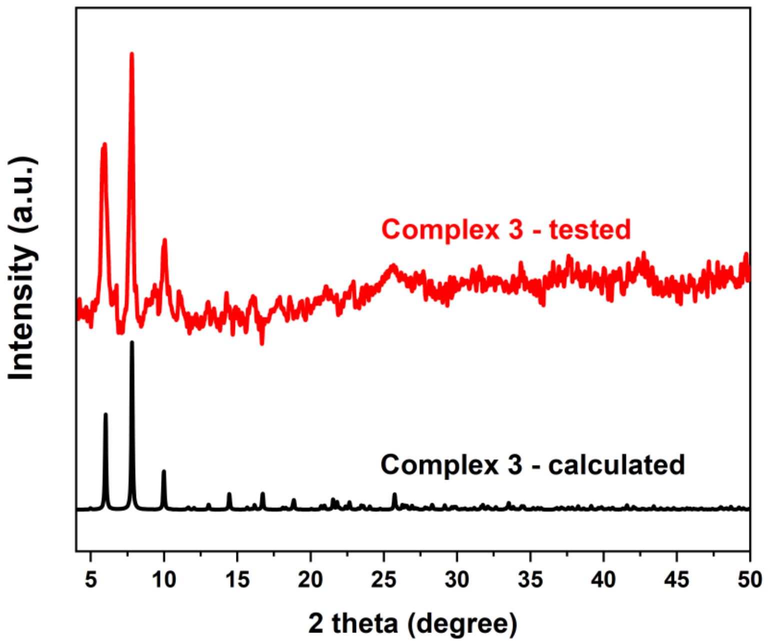

Fig. S2 XRD patterns of complex **3**.

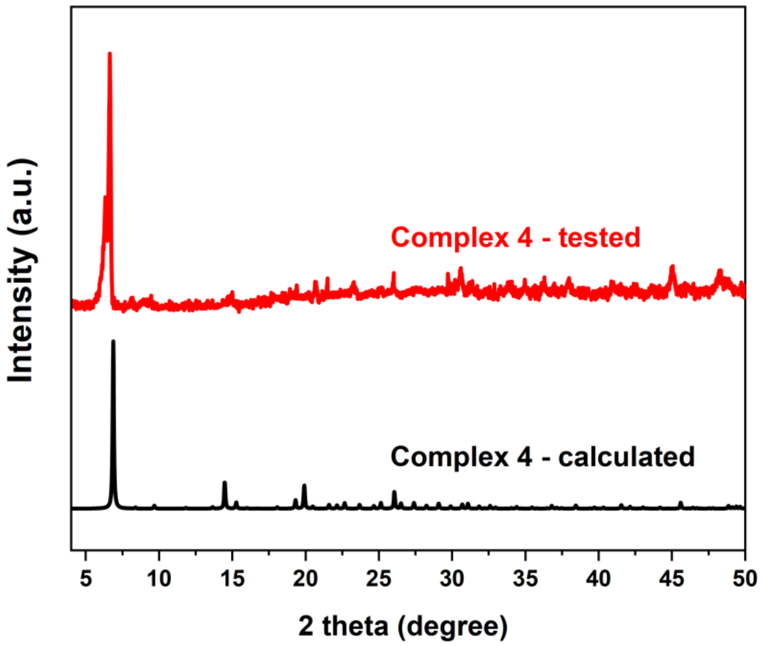

Fig. S3 XRD patterns of complex **4**.

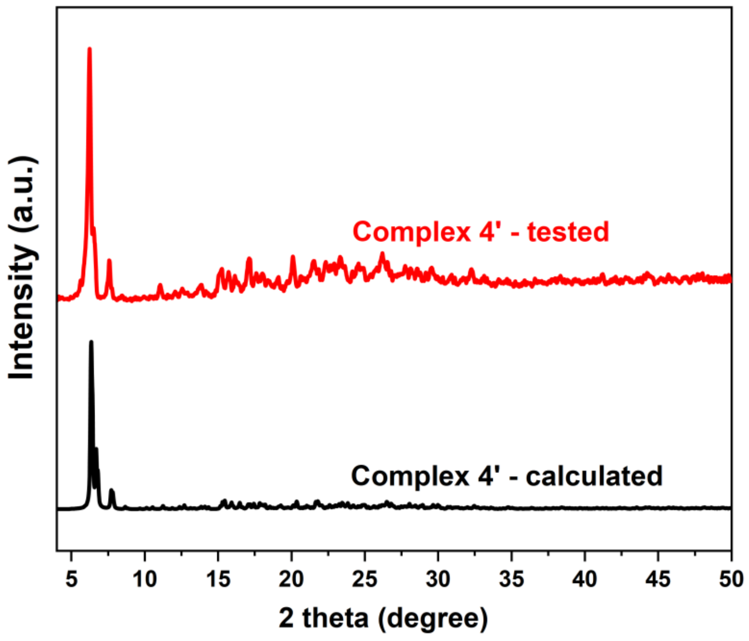

Fig. S4 XRD patterns of complex **4'**.

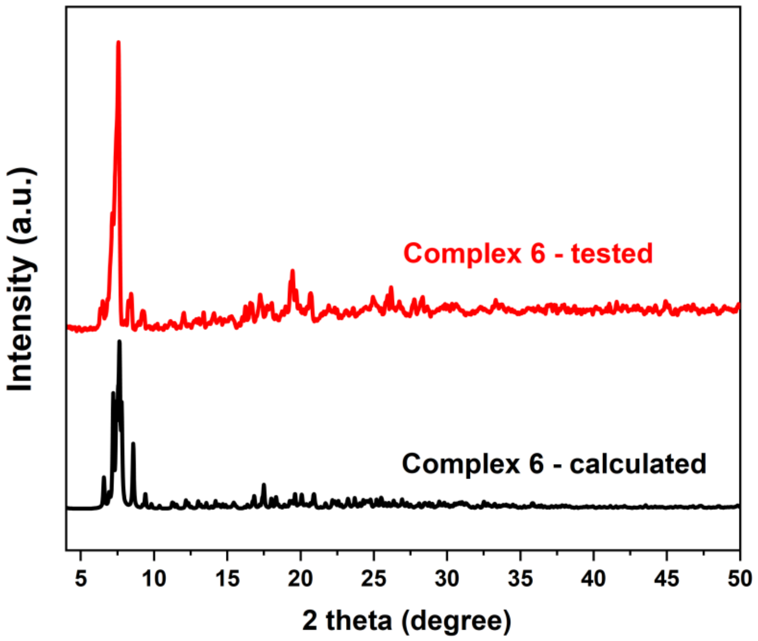

Fig. S5 XRD patterns of complex **6**.

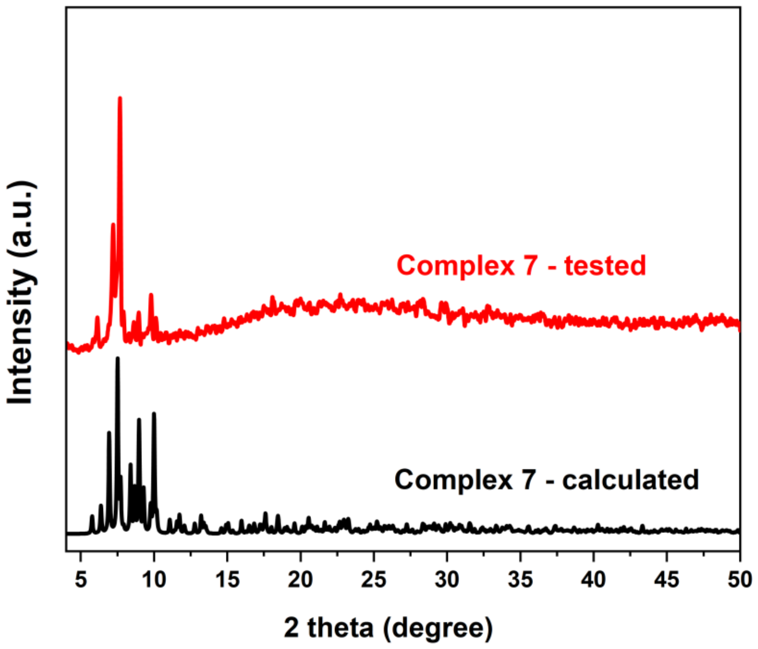

Fig. S6 XRD patterns of complex **7**.

4. Cyclic voltammetry tests for Tetranuclear Copper(I) Clusters

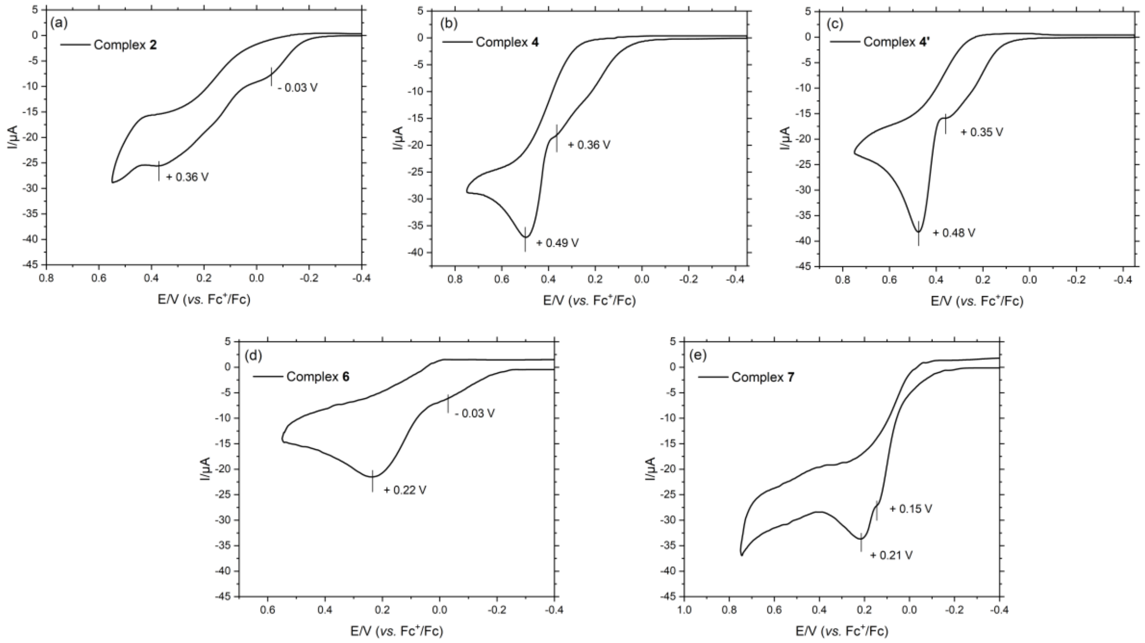

Fig. S7 Cyclic voltammogram of complex **2, 4, 4’, 6, and 7** in MeCN (0.1 M $^nBu_4PF_6$ 298 K) at a scan rate of 100 mV s^{-1}.

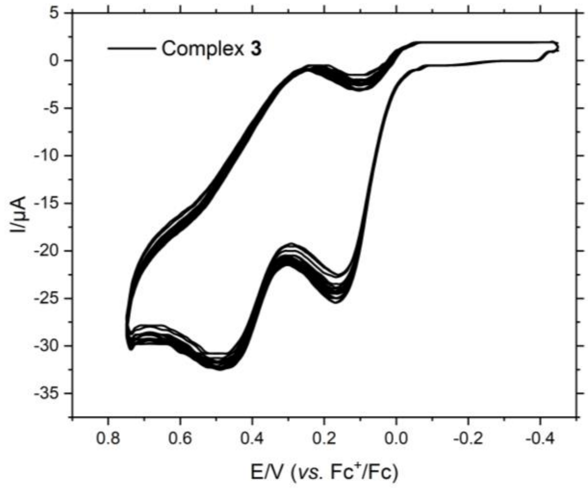

Fig. S8 Cyclic voltammogram of complex **3** in MeCN (0.1 M $^nBu_4PF_6$ 298 K) at a scan rate of 100 mV s^{-1} for 20 cycles.

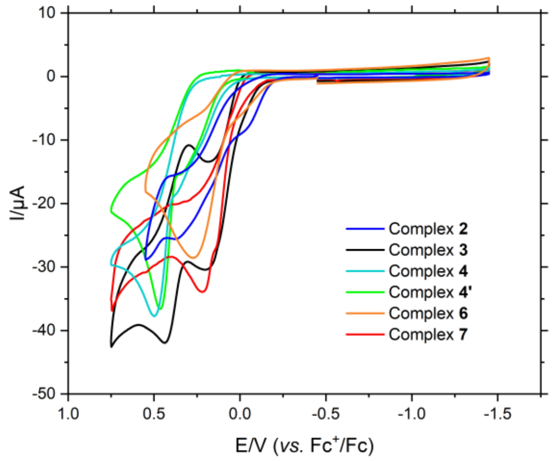

Fig. S9 Comparison of the oxidation process of complexes **2**, **3**, **4**, **4’**, **6**, and **7** from -1.5 eV-1.0 eV.

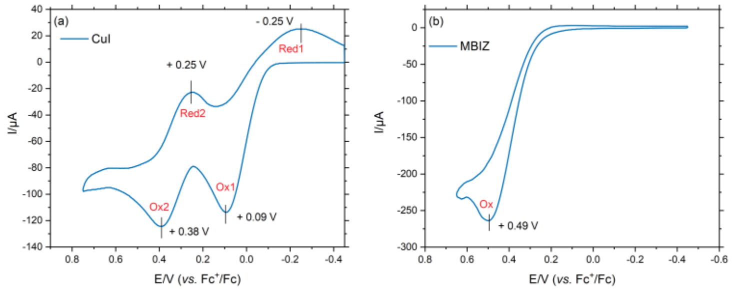

Fig. S10 Cyclic voltammogram of CuI and MBIZ in MeCN (0.1 M $^{n}Bu_4PF_6$ 298 K) at a scan rate of 100 mV s^{-1}.

Table S1. The reversibility of complex **3** and CuI for oxidation-reduction peak2

	I_{ox} ($\times10^{-5}$)	I_{red} ($\times10^{-5}$)	Reversibility (Reduction rate)
CuI	4.92	4.47	90.85%
Complex 3	1.11	0.73	65.77%

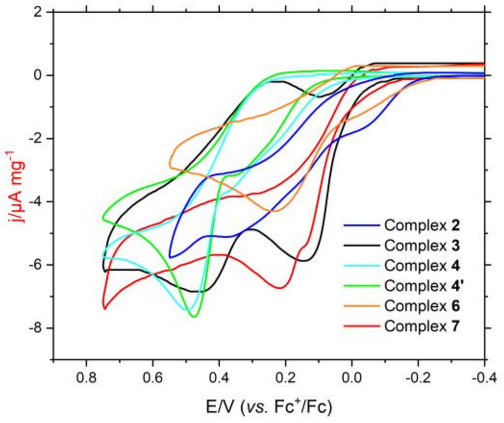

Fig. S11 Cyclic voltammogram of complexes **2**, **3**, **4**, **4’**, **6**, and **7.**

5. DSC (differential scanning calorimetry) of complex 3

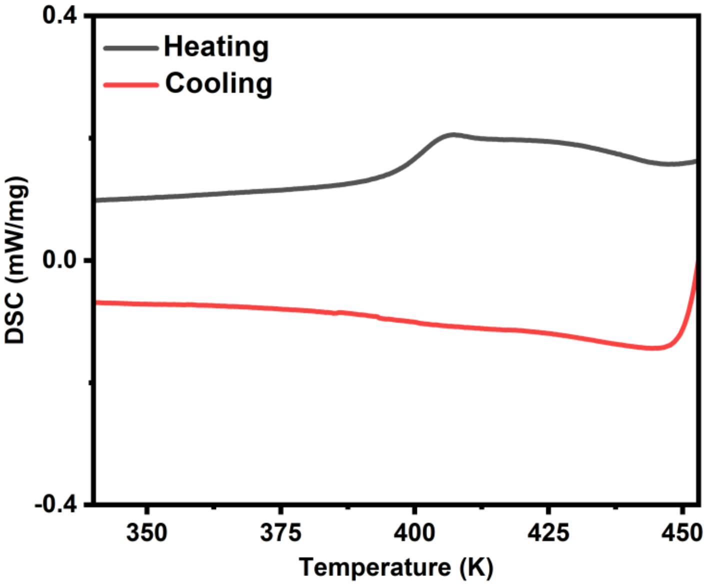

Fig. S12 Differential Scanning Calorimetry of Complex **3**.

6. X-ray Structure Determinations

Diffraction data were collected on an Oxford Diffraction Supernova dual diffractometer equipped with an Oxford Cryostream 700 low-temperature apparatus. Cu K\a radiation source (λ = 1.54184 Å) was used for the data collection. Single crystals were coated with Paratone-N oil and mounted on a Nylon loop for diffraction. The data reduction and cell refinement were processed using CrysAlisPro software.[6] Structures were solved by direct methods using the SHELXTL program packages.[7] All non-hydrogen atoms were refined anisotropically and hydrogen atoms were added geometrically. All the structures are finally refined using a modern refinement program.[8] Crystal data and refinement details were given in Tables S1. Other refinement details and explanations were included in individual CIF files.

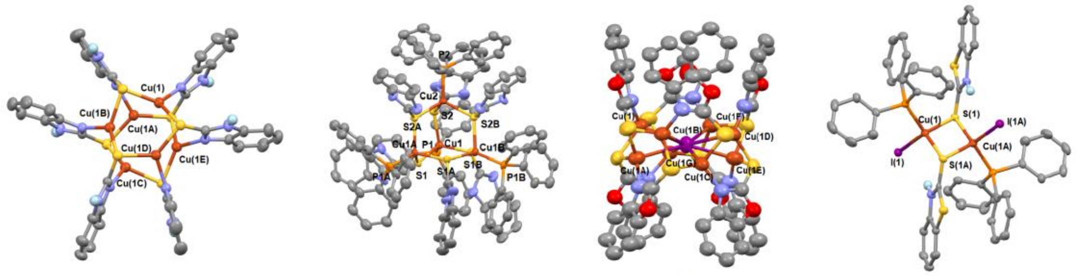

1 4 5 8

Fig. S13 Crystal structures of complexes **1** (fast data), **4** (fast data), **5** (fast data), **8** with all non-hydrogen atoms shown as 50% probability ellipsoids.

7. Crystallographic data of compounds

Table S2. Crystallographic data[a] for complex **2**, **3**, **4'**

	**2** (2336741)	**3** (2336735)	**4'** (2374006)
formula	$C_{78}H_{78}Cu_4N_8O_{3.5}P_2S_4$	$C_{89}H_{87}Cu_4IN_7O_3P_3S_3$	$C_{128}H_{126}B_4Cu_4F_{16}N_{13}O_{1.5}P_4S_6$
M	1627.82	1872.80	2788.05
crystal system	triclinic	trigonal	triclinic
space group	P -1	P-3	P -1
a, Å	14.9470(6)	16.9492(6)	16.4700(3)
b, Å	15.3365(4)	16.9492(6)	17.0044(2)
c, Å	18.2930(6)	17.6895(8)	28.5015(4)
α, deg	88.059(3)	90.00	81.4516(11)
β, deg	91.459(2)	90.00	74.1699(13)
γ, deg	64.577(3)	120.00	61.1106(16)
V, $Å^3$	4730.84(18)	4400.9(4)	6722.4(2)
Z	2	2	2
μ, mm^{-1}	3.130	5.422	2.667
independent data	14213	5472	25399
refined parameters	1058	332	2058
$R_1{}^b$, $wR_2{}^c(I>2\sigma(I))$	0.0568, 0.1630	0.0802, 0.2145	0.0672, 0.2073
R_1,wR_2 (all data)	0.0704, 0.1763	0.0872, 0.2195	0.0783, 0.2205

[a]T = 150(2) K, Cu Kα radiation (λ = 1.54178 Å). [b]$R_1 = \Sigma||F_o| - |F_c||/\Sigma|F_o|$. [c]$wR_2 = \{\Sigma[w(Fo^2 - F_c{}^2)^2/(F_o{}^2)^2]\}^{1/2}$.

Table S3. Crystallographic data[a] for complex **6**, **7**, **8**

	**6** (2374007)	**7** (2374008)	**8** (2336717)
formula	$C_{100}H_{76}Cu_4N_4O_4P_4S_4$	$C_{76}H_{58}Cl_3Cu_4IN_3O_3P_3S_3$	$C_{50}H_{40}Cu_2I_2N_2P_2S_4$
M	1903.92	1737.75	1239.90
crystal system	triclinic	monoclinic	monoclinic
space group	P -1	P 21/n	C 2/c
a, Å	14.1645(3)	15.8043(2)	25.6927(6)
b, Å	14.6533(3)	22.2664(4)	9.2577(2)
c, Å	23.8066(5)	21.4443(3)	20.2000(4)
α, deg	81.6263(19)	90	90
β, deg	76.0245(19)	101.3164(15)	100.058(2)
γ, deg	66.465(2)	90	90
V, $Å^3$	4389.39(18)	7399.7(2)	4730.84(18)
Z	2	4	4
μ, mm^{-1}	3.109	7.360	13.938
independent data	16607	13933	4445
refined parameters	1223	902	284
$R_1{}^b$, $wR_2{}^c(I>2\sigma(I))$	0.0335, 0.0877	0.0586, 0.1502	0.0332, 0.0840
R_1,wR_2 (all data)	0.0391, 0.0913	0.0747, 0.1606	0.0840, 0.0893

[a]T = 150(2) K, Cu Kα radiation (λ = 1.54178 Å). [b]$R_1 = \Sigma||F_o| - |F_c||/\Sigma|F_o|$. [c]$wR_2 = \{\Sigma[w(Fo^2 - F_c{}^2)^2/(F_o{}^2)^2]\}^{1/2}$.

8. ^{1}H NMR and ^{13}C NMR spectra of compounds

Complex **2**

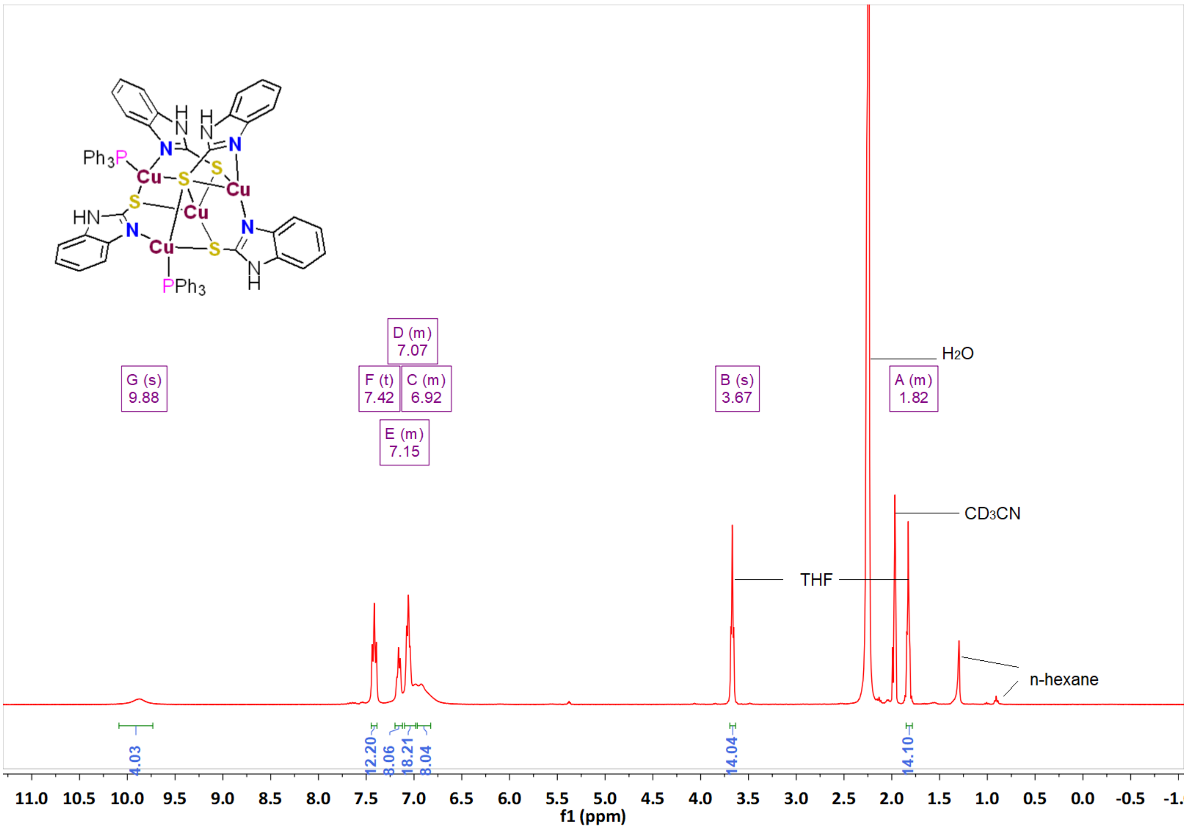

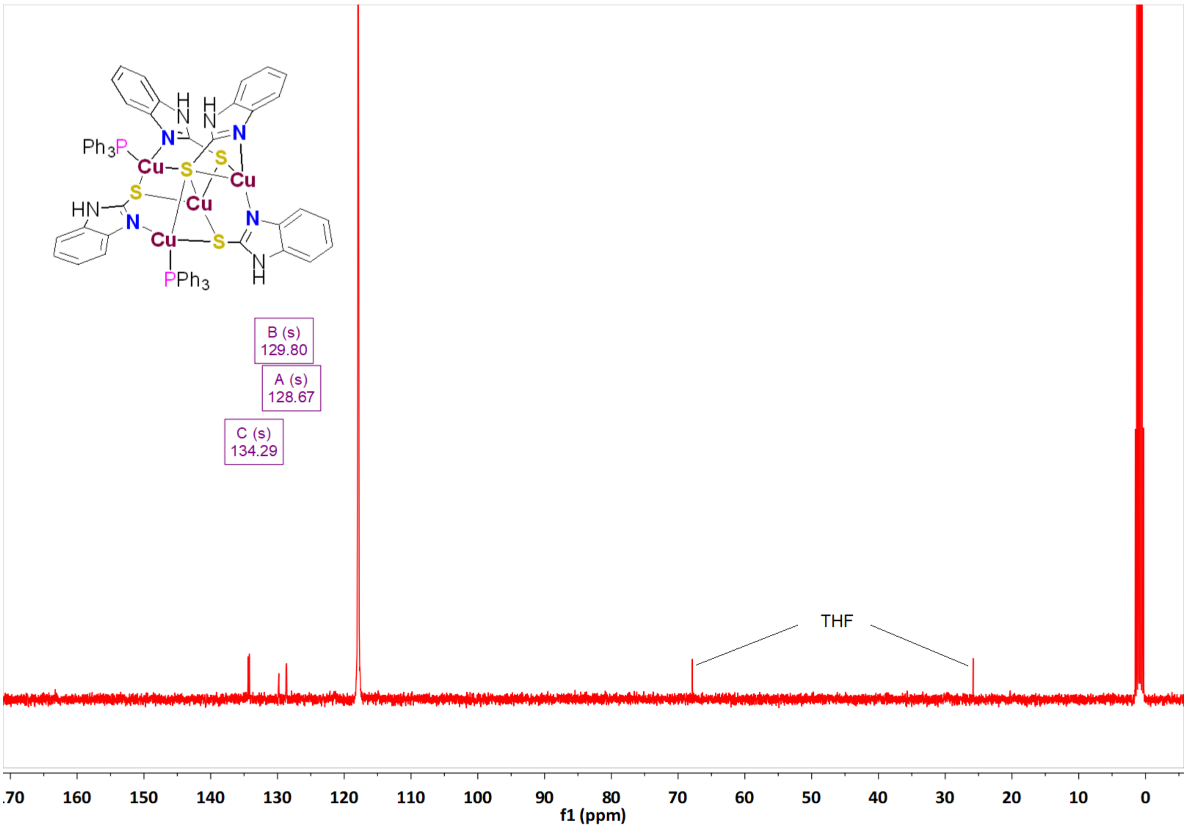

Complex **3**

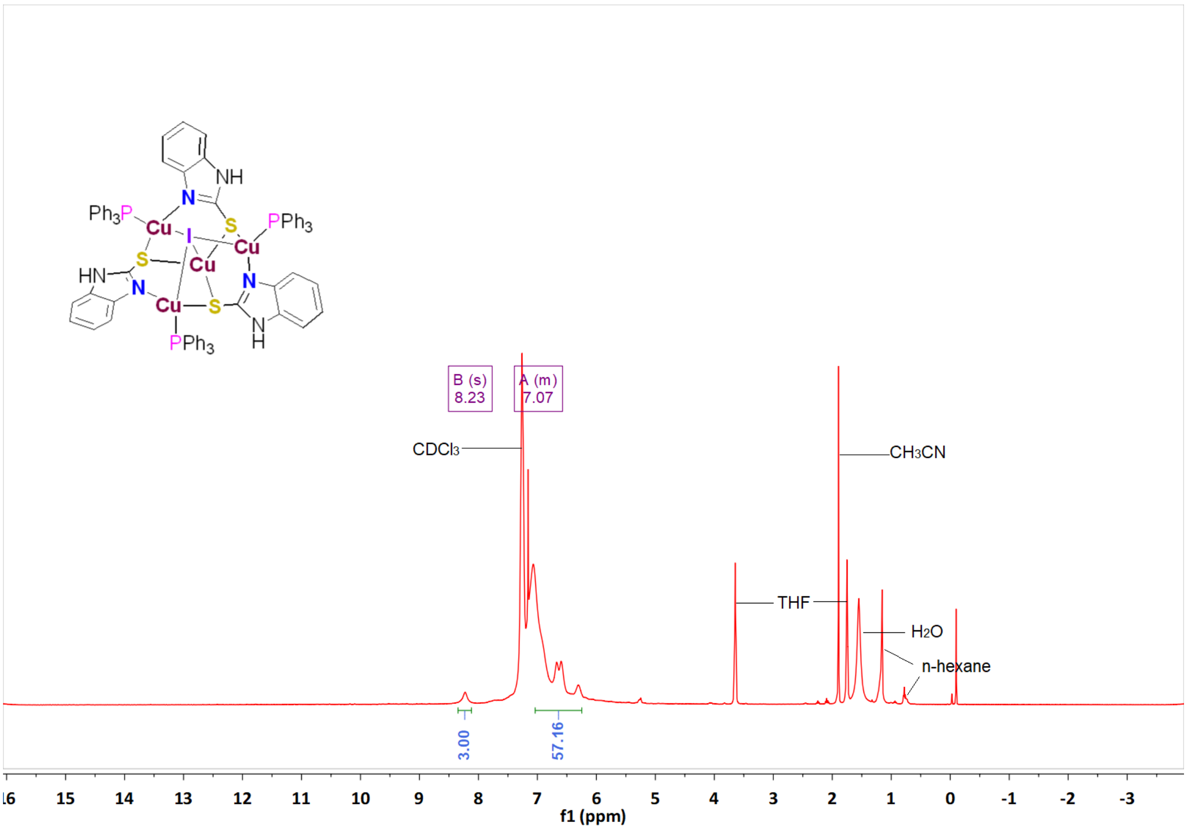

NH
Ph3P
PPh3
Cu
I
S
N
HN
PPh3
H
B (s)
8.23
A (m)
7.07
CDCl3
CH3CN
THF
H2O
n-hexane
3.00
57.16
16
15
14
13
12
11
10
9
8
7
6
5
4
3
2
1
0
-1
-2
-3
f1 (ppm)

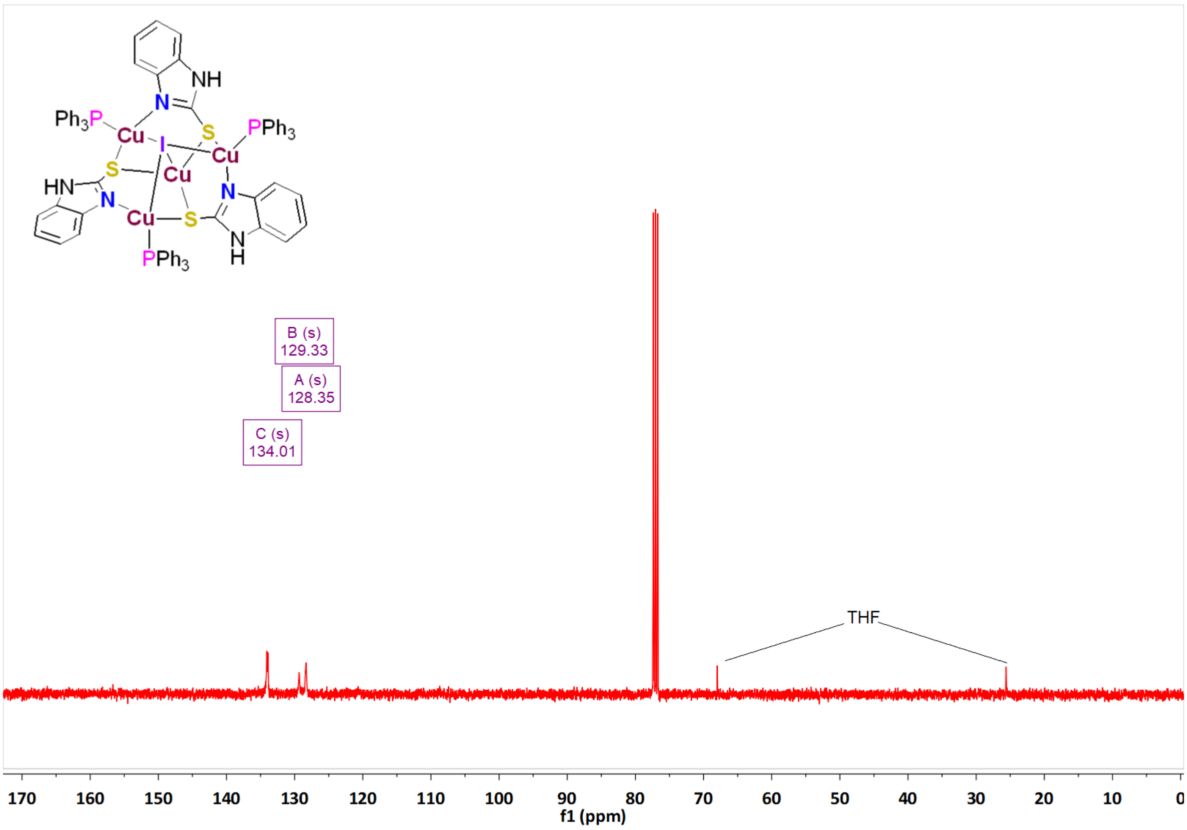

NH
Ph3P
PPh3
Cu
I
S
N
HN
PPh3
H
B (s)
129.33
A (s)
128.35
C (s)
134.01
THF
170
160
150
140
130
120
110
100
90
80
70
60
50
40
30
20
10
0
f1 (ppm)

Complex **4**

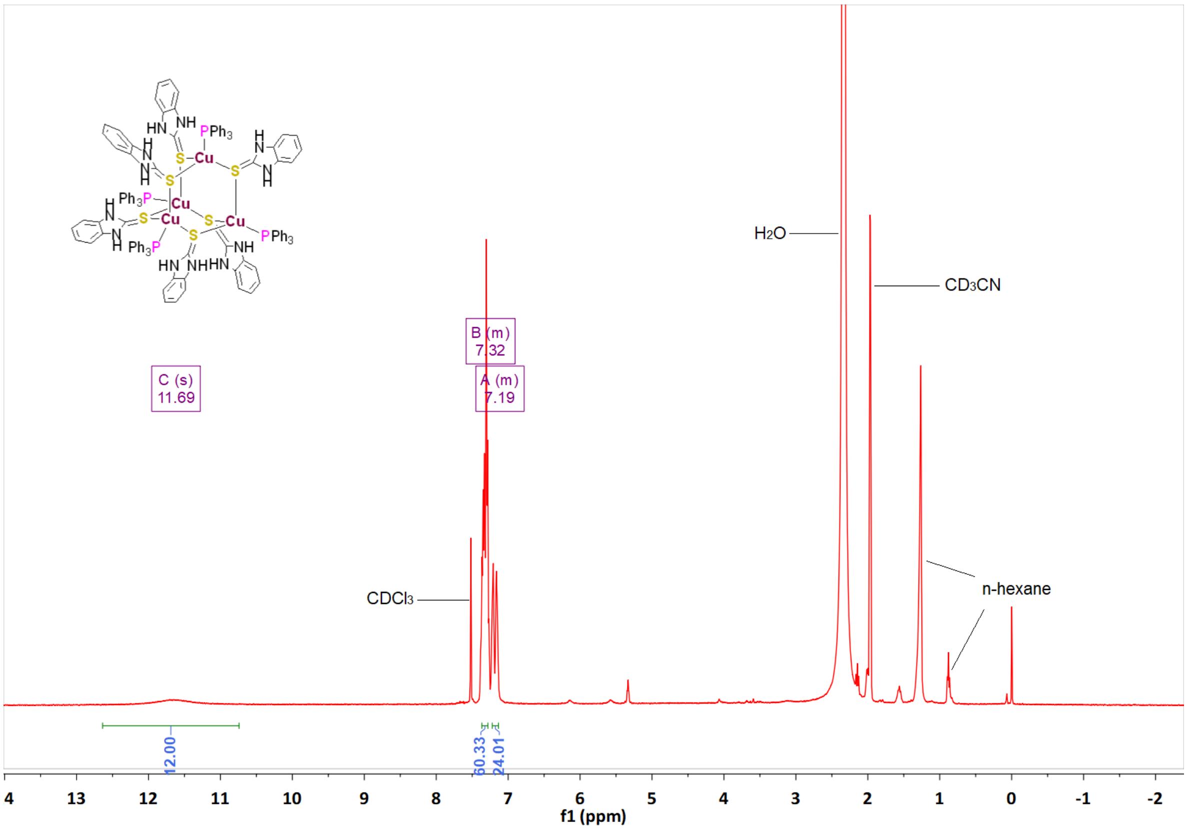

HN NH PPh3
S Cu
Ph3P Cu
S Cu S Cu PPh3
Ph3P
HN NHHN NH
H2O
CD3CN
B (m)
7.32
A (m)
7.19
C (s)
11.69
CDCl3
n-hexane
12.00
60.33
24.01
f1 (ppm)

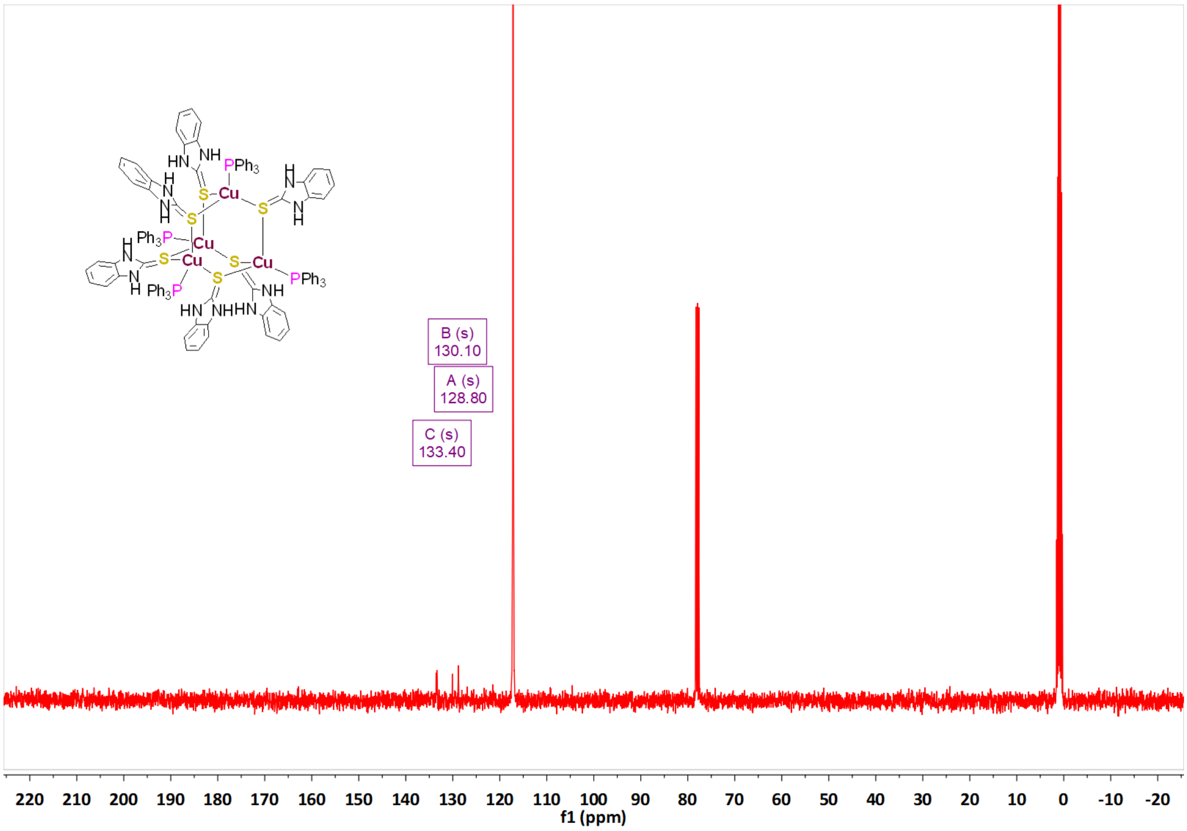

HN NH PPh3
S Cu
Ph3P Cu
S Cu S Cu PPh3
Ph3P
HN NHHN NH
B (s)
130.10
A (s)
128.80
C (s)
133.40
f1 (ppm)

Complex **4’**

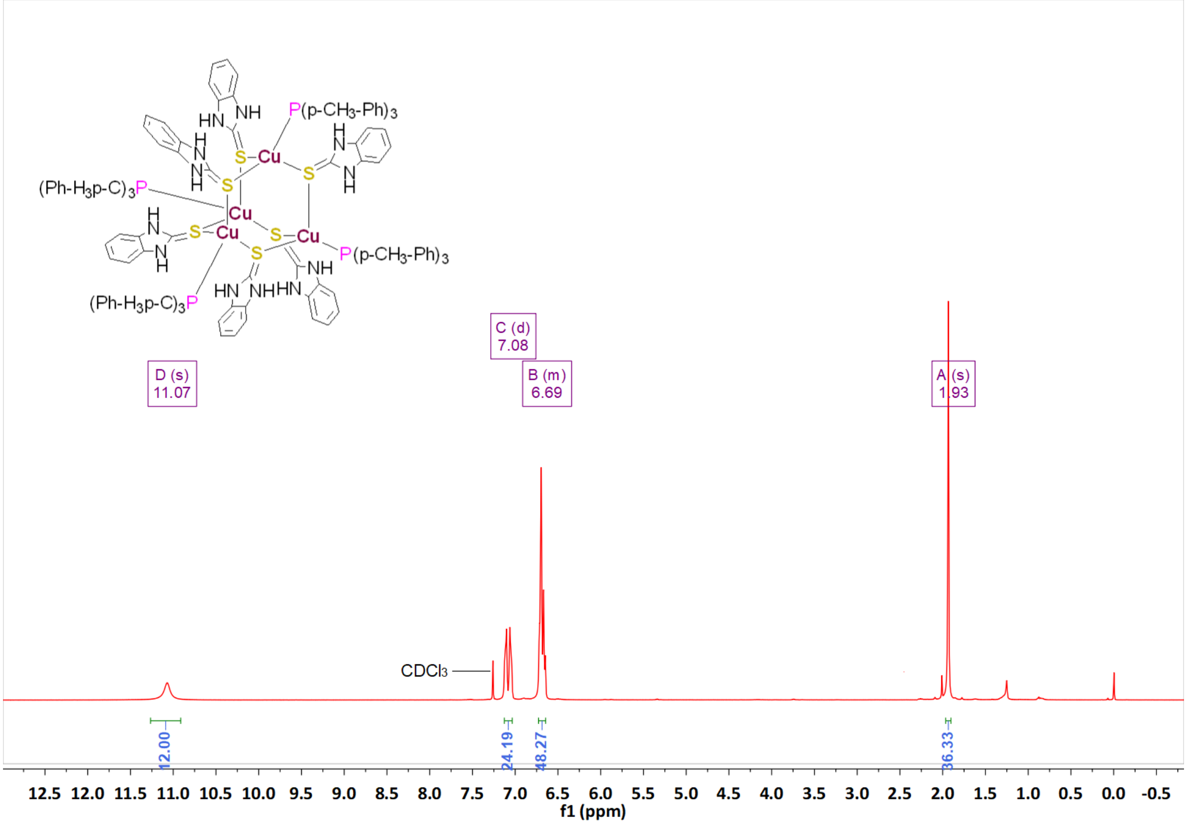
P(p-CH3-Ph)3
(Ph-H3p-C)3P
Cu
S
HN
NH
C (d)
7.08
D (s)
11.07
B (m)
6.69
A (s)
1.93
CDCl3
12.00
24.19
48.27
36.33
12.5 12.0 11.5 11.0 10.5 10.0 9.5 9.0 8.5 8.0 7.5 7.0 6.5 6.0 5.5 5.0 4.5 4.0 3.5 3.0 2.5 2.0 1.5 1.0 0.5 0.0 -0.5
f1 (ppm)

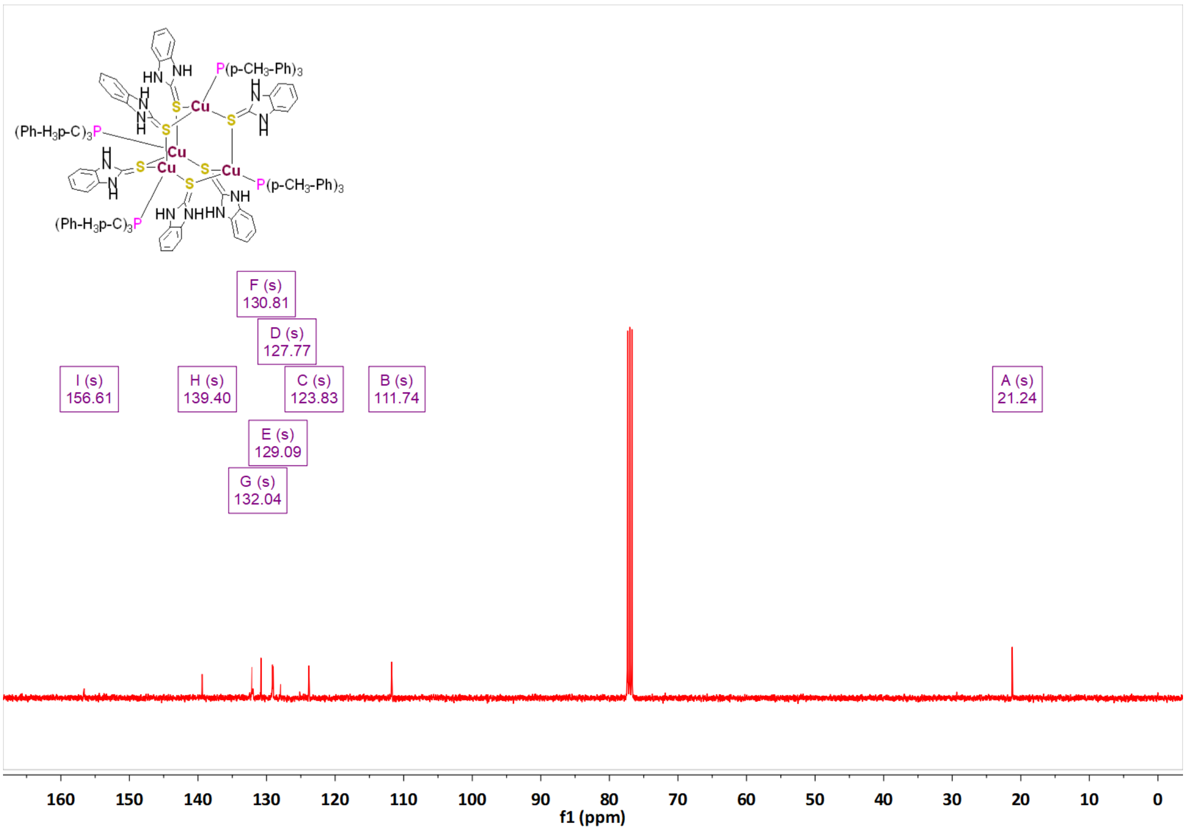
P(p-CH3-Ph)3
(Ph-H3p-C)3P
F (s)
130.81
D (s)
127.77
I (s)
156.61
H (s)
139.40
C (s)
123.83
B (s)
111.74
A (s)
21.24
E (s)
129.09
G (s)
132.04
160 150 140 130 120 110 100 90 80 70 60 50 40 30 20 10 0
f1 (ppm)

Complex **6**

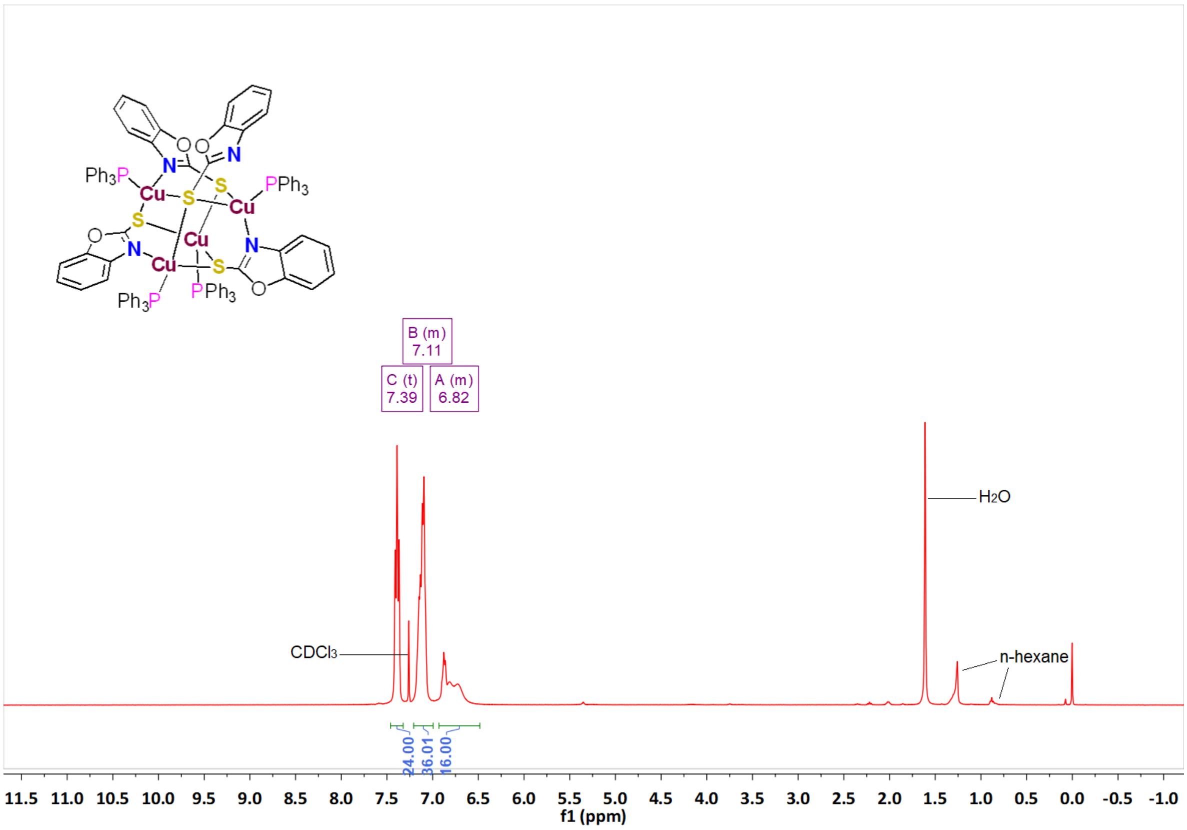
B (m)
7.11
C (t)
7.39
A (m)
6.82
H2O
CDCl3
n-hexane
24.00
36.01
16.00
11.5 11.0 10.5 10.0 9.5 9.0 8.5 8.0 7.5 7.0 6.5 6.0 5.5 5.0 4.5 4.0 3.5 3.0 2.5 2.0 1.5 1.0 0.5 0.0 -0.5 -1.0
f1 (ppm)

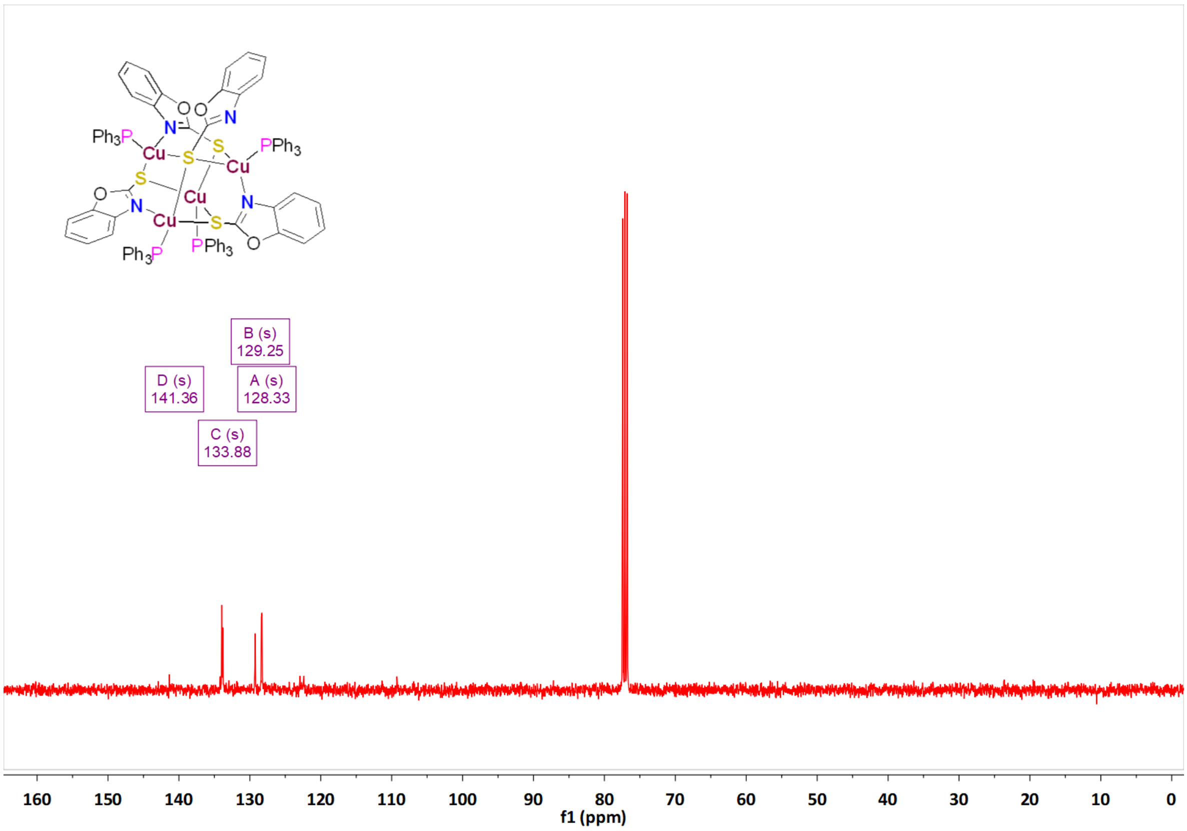
B (s)
129.25
D (s)
141.36
A (s)
128.33
C (s)
133.88
160 150 140 130 120 110 100 90 80 70 60 50 40 30 20 10 0
f1 (ppm)

Complex **7**

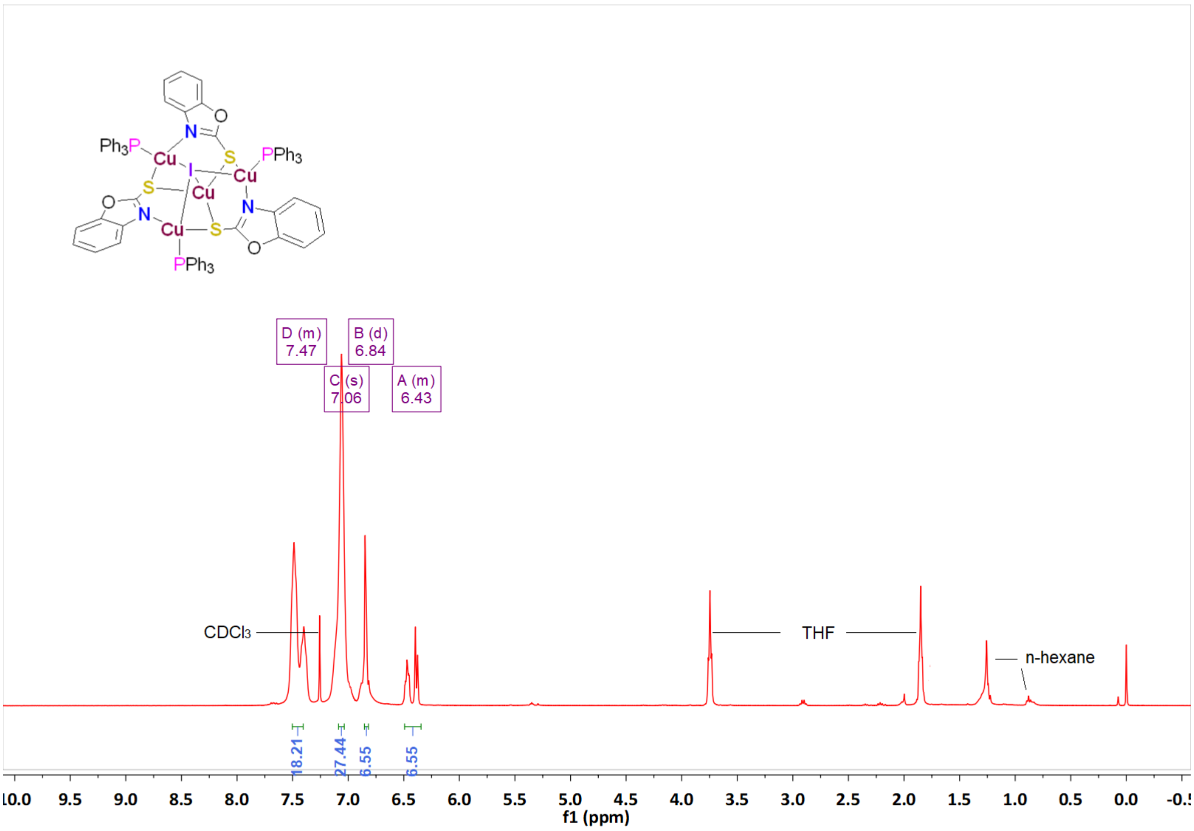

Ph3P
PPh3
PPh3
Cu
Cu
Cu
Cu
I
S
N
O
D (m)
7.47
B (d)
6.84
C (s)
7.06
A (m)
6.43
CDCl3
THF
n-hexane
18.21
27.44
6.55
6.55
10.0
9.5
9.0
8.5
8.0
7.5
7.0
6.5
6.0
5.5
5.0
4.5
4.0
3.5
3.0
2.5
2.0
1.5
1.0
0.5
0.0
f1 (ppm)

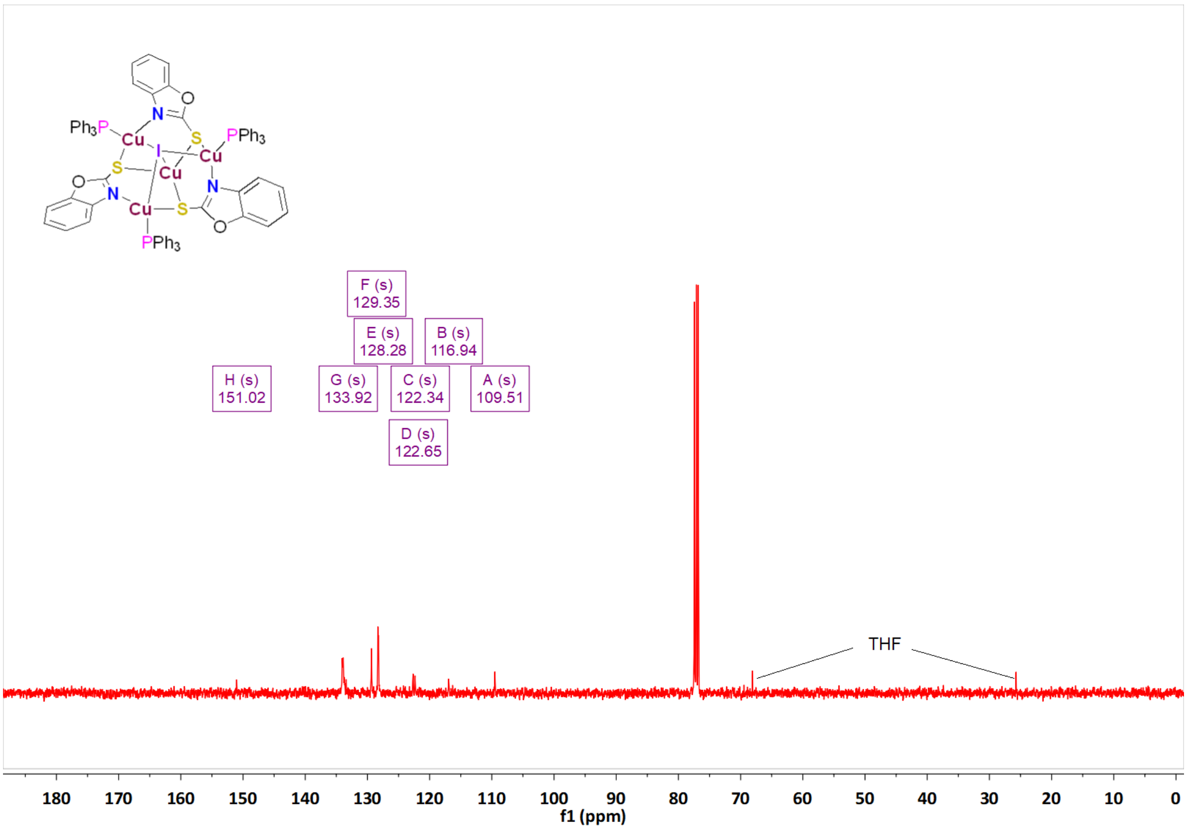

Ph3P
PPh3
PPh3
Cu
Cu
Cu
Cu
I
S
N
O
F (s)
129.35
E (s)
128.28
B (s)
116.94
H (s)
151.02
G (s)
133.92
C (s)
122.34
A (s)
109.51
D (s)
122.65
THF
180
170
160
150
140
130
120
110
100
90
80
70
60
50
40
30
20
10
0
f1 (ppm)

Compound **10a**

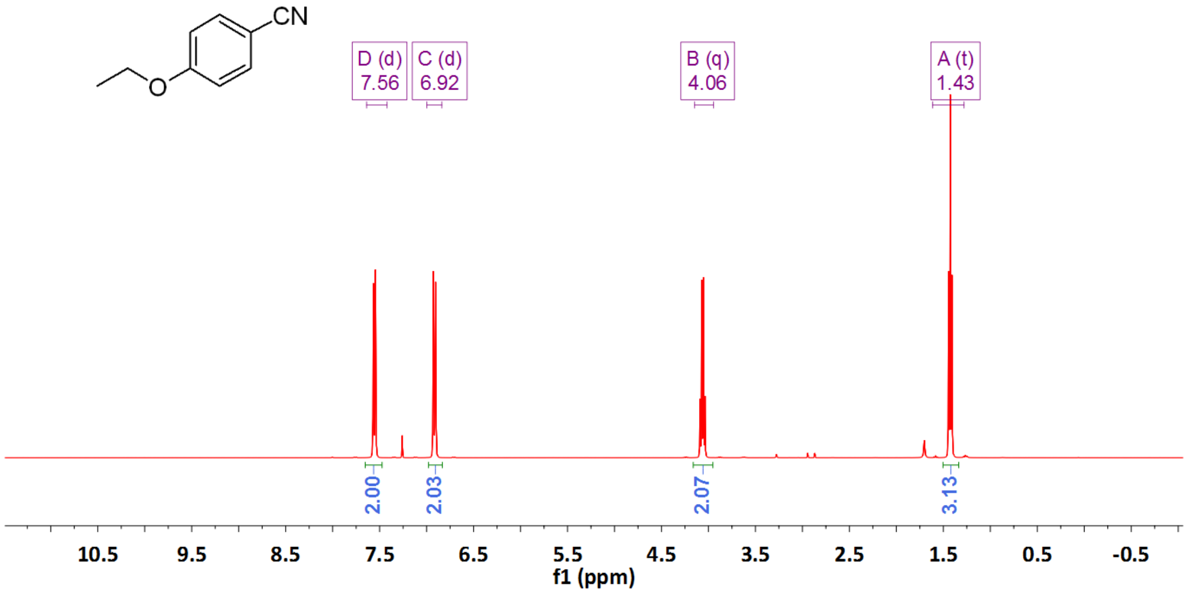

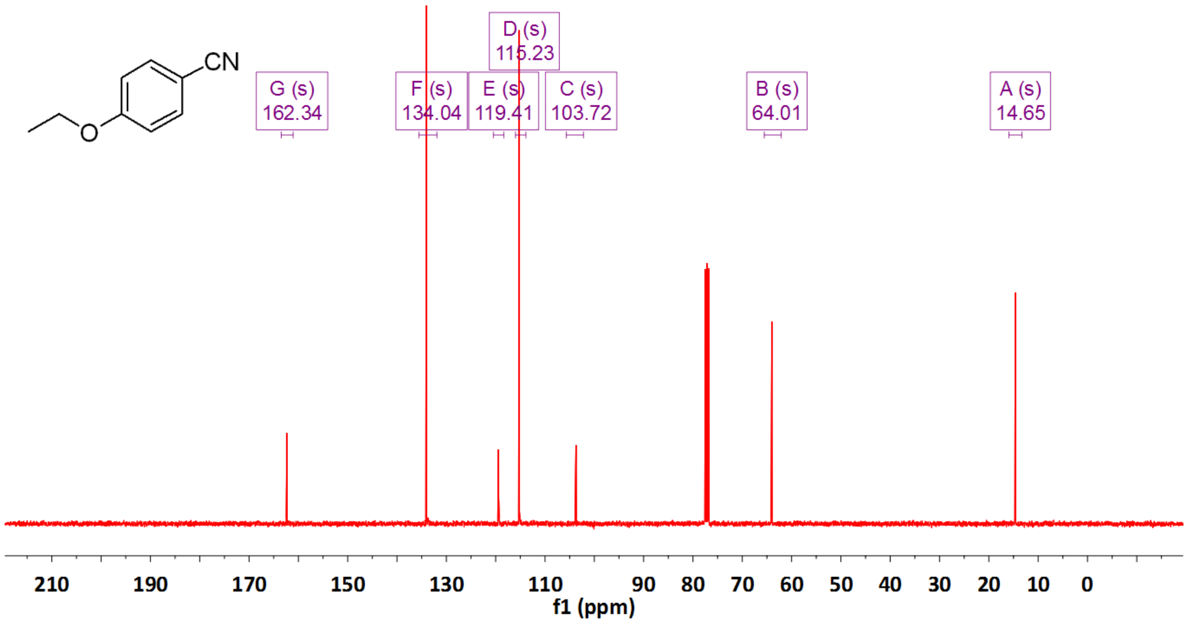

Compound **10b**

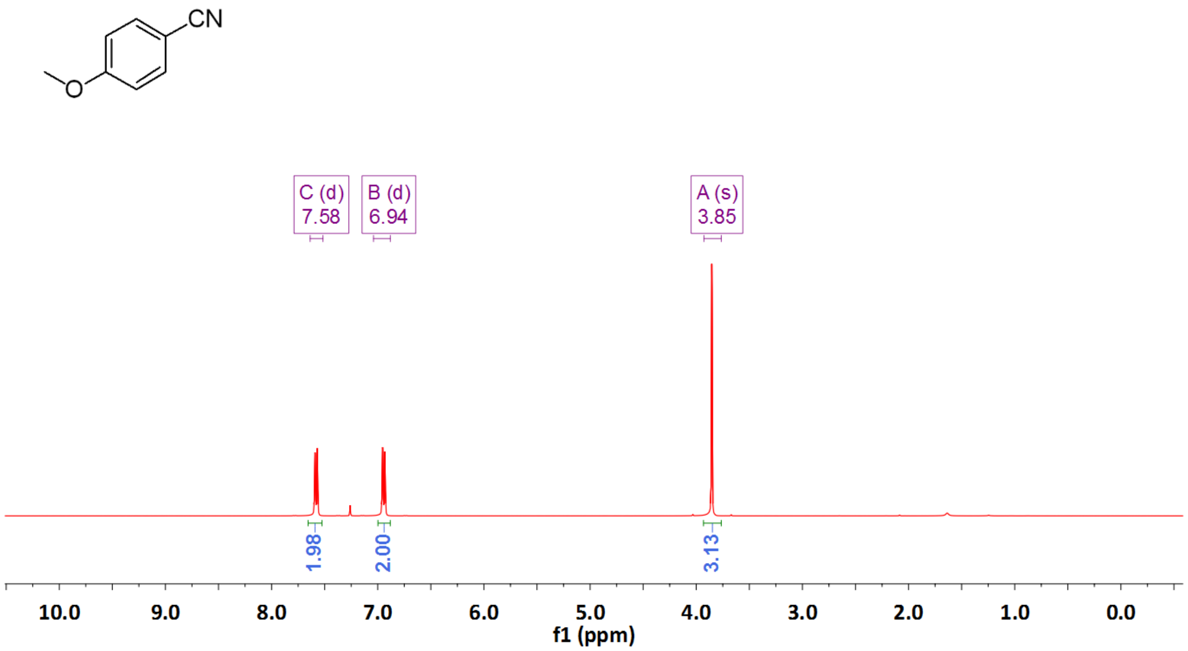

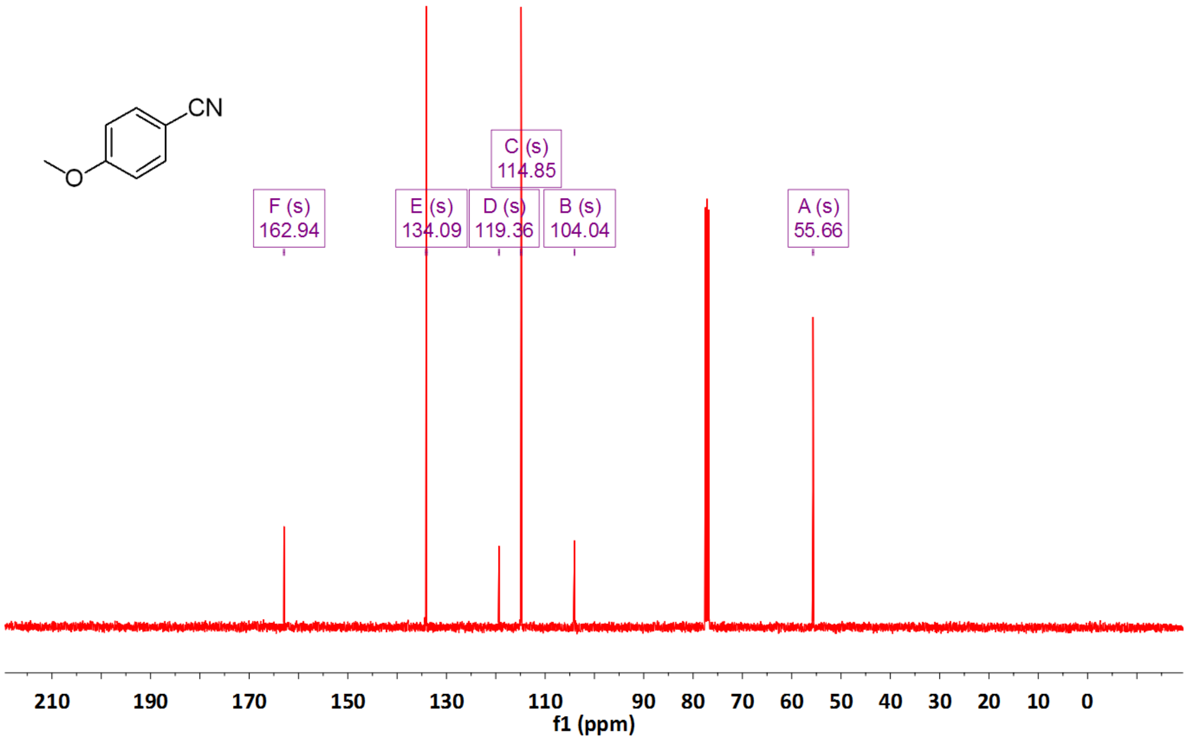

Compound **10c**

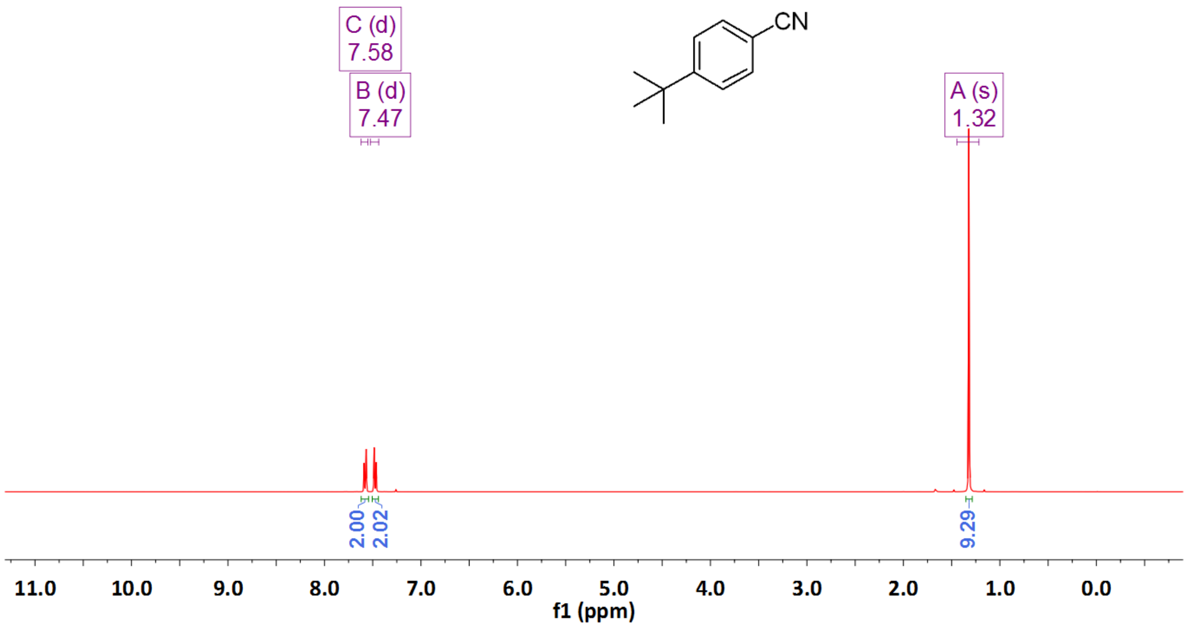

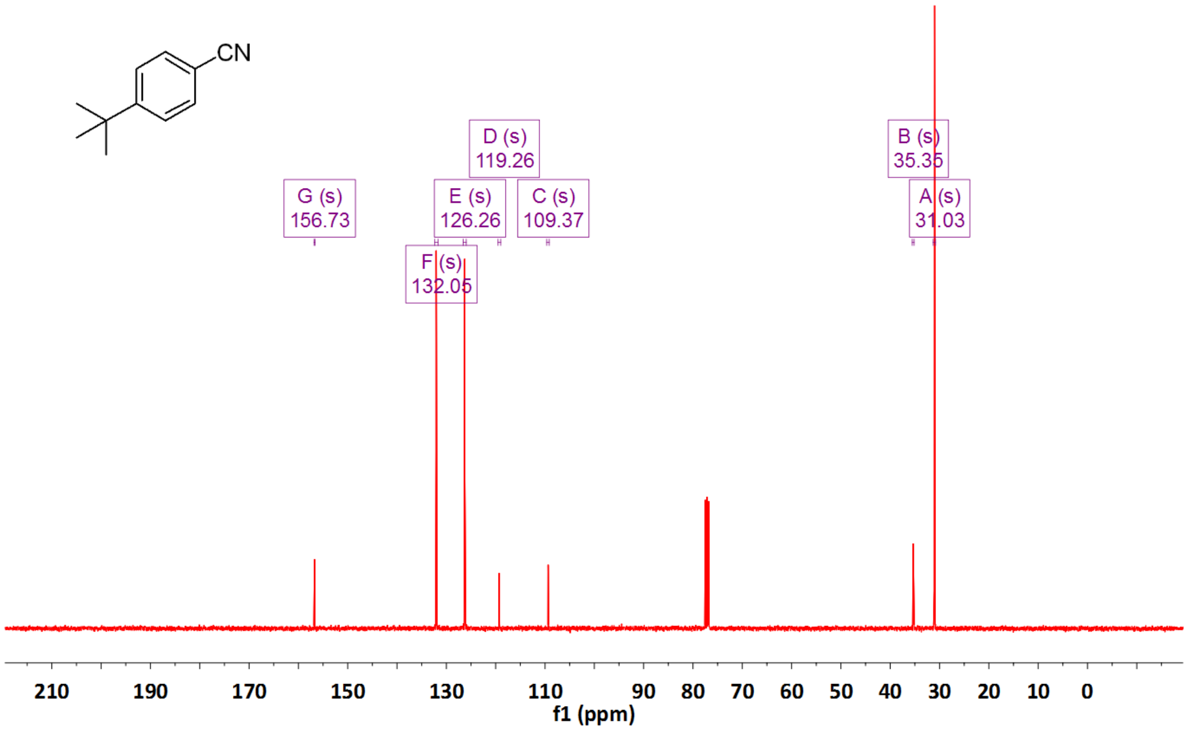

Compound **10d**

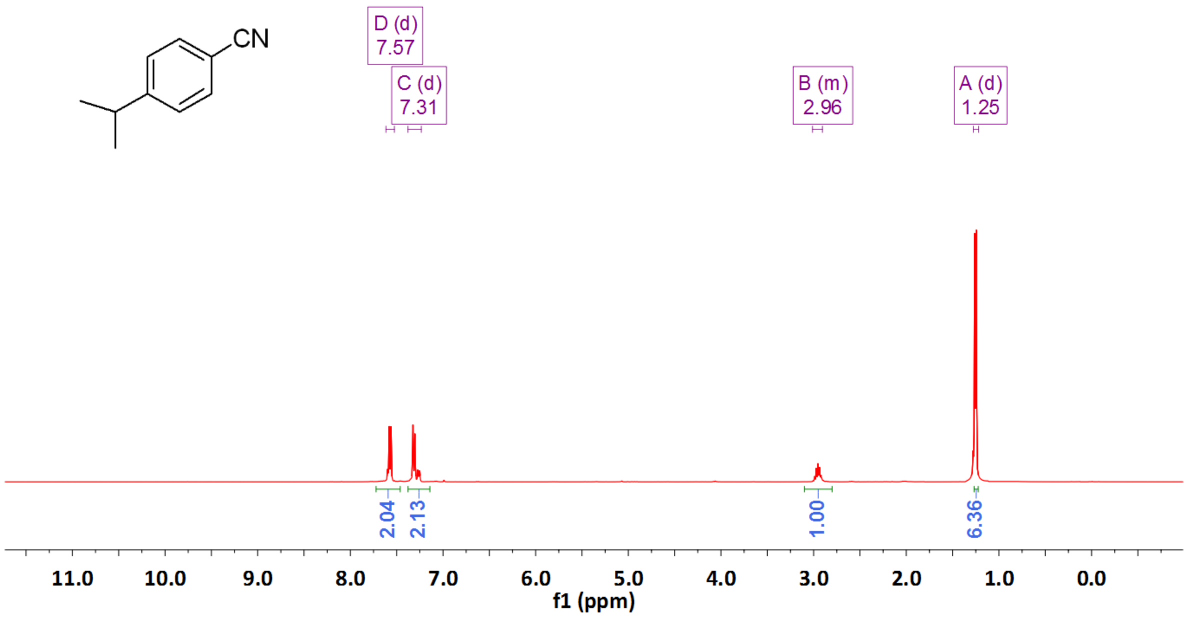

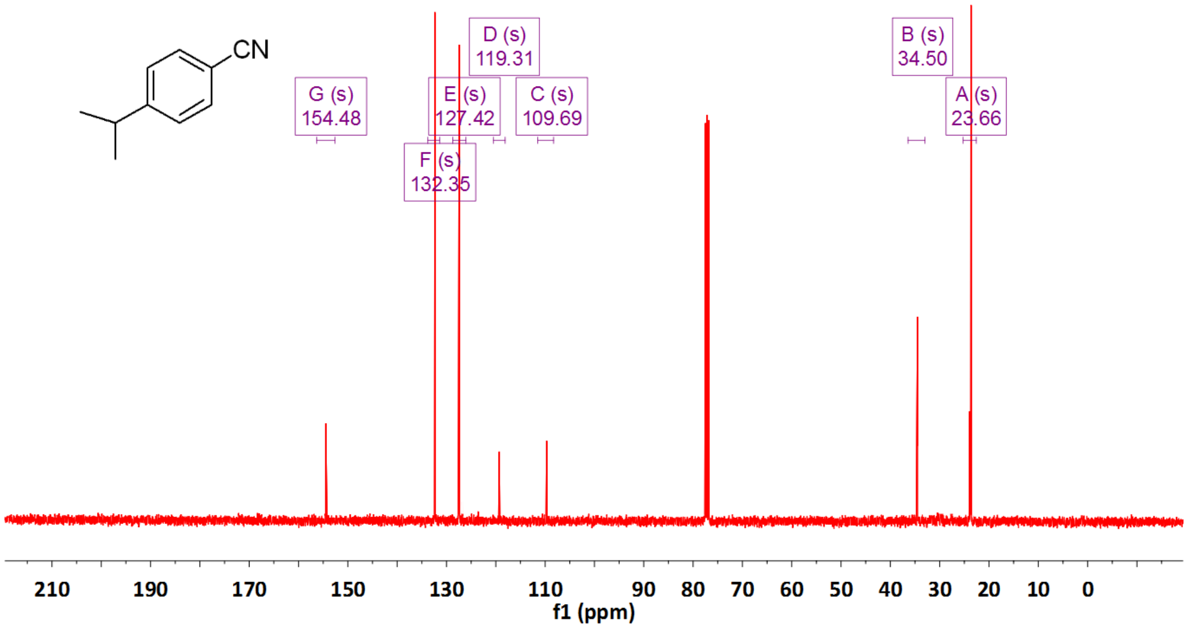

Compound **10e**

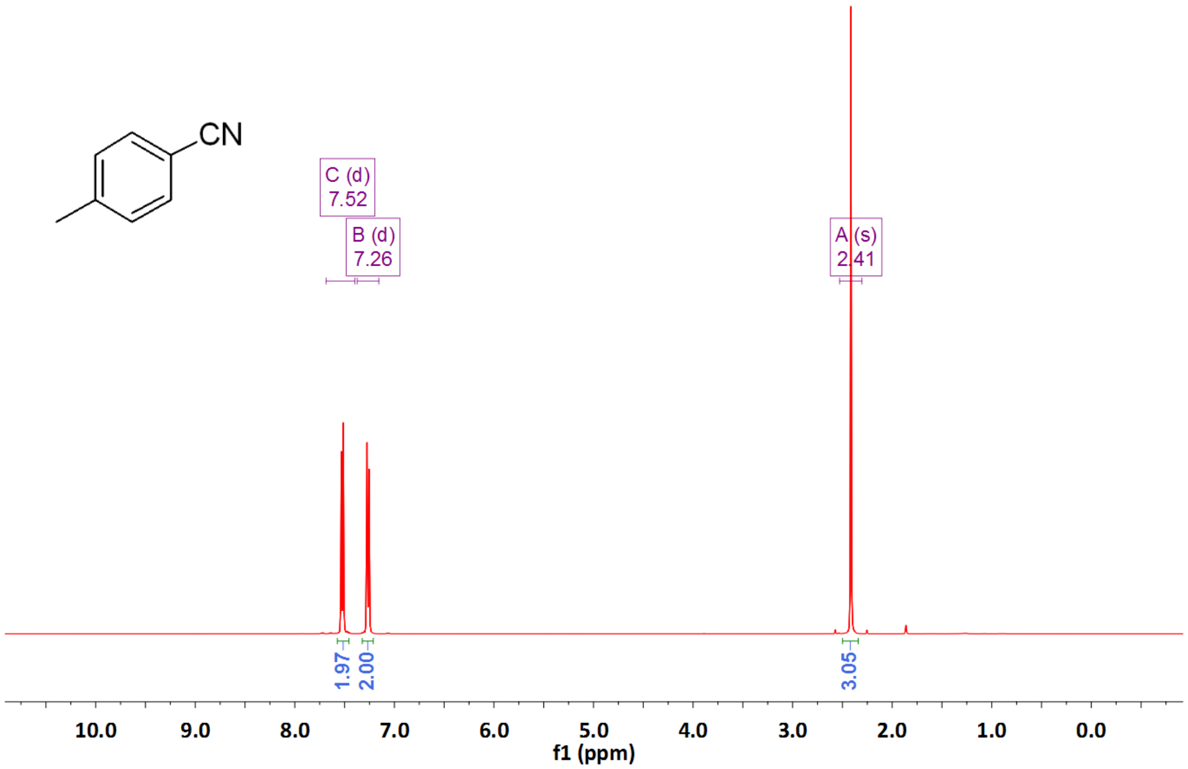

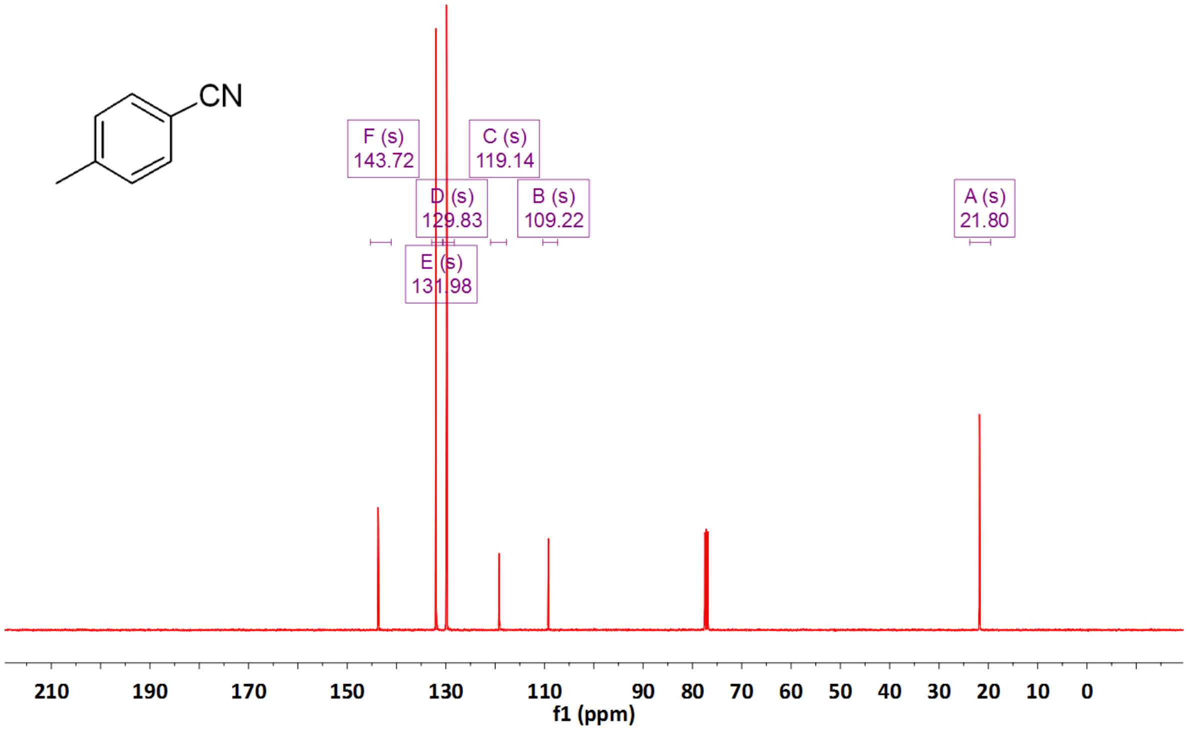

Compound **10f**

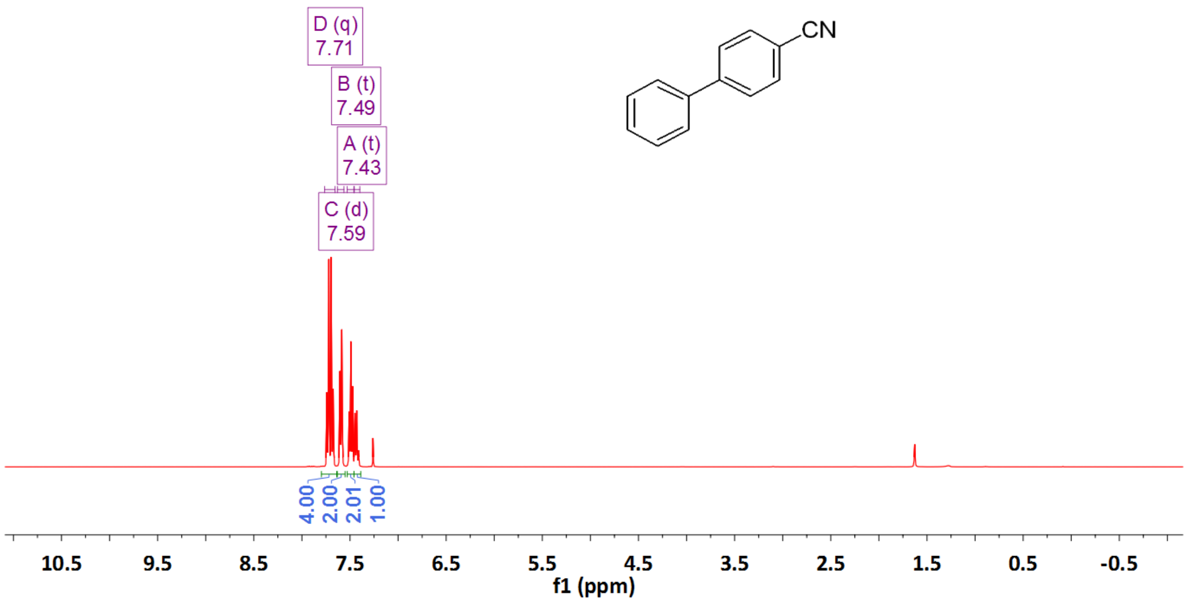

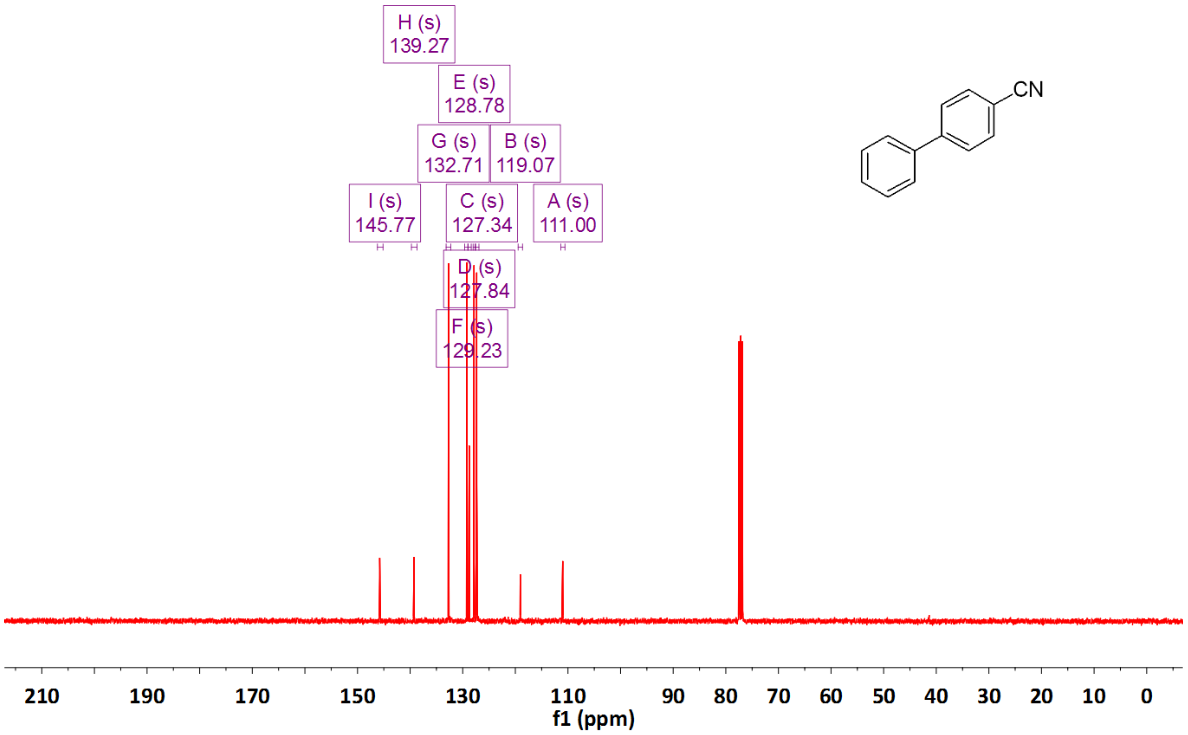

Compound **10g**

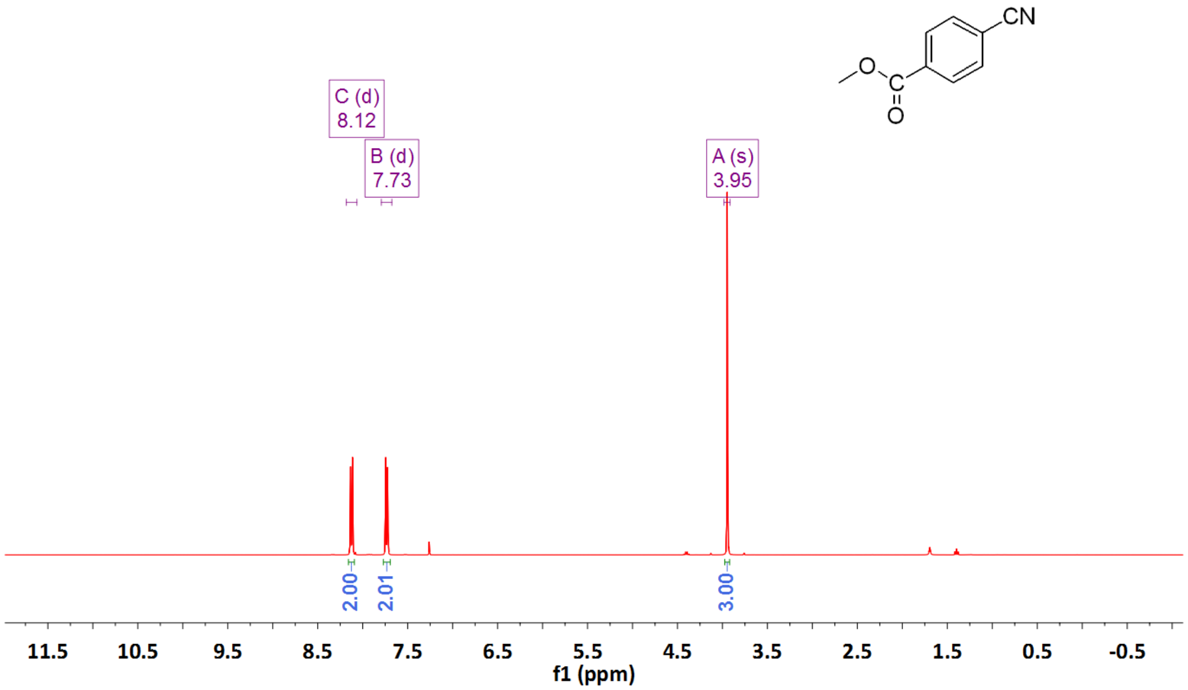

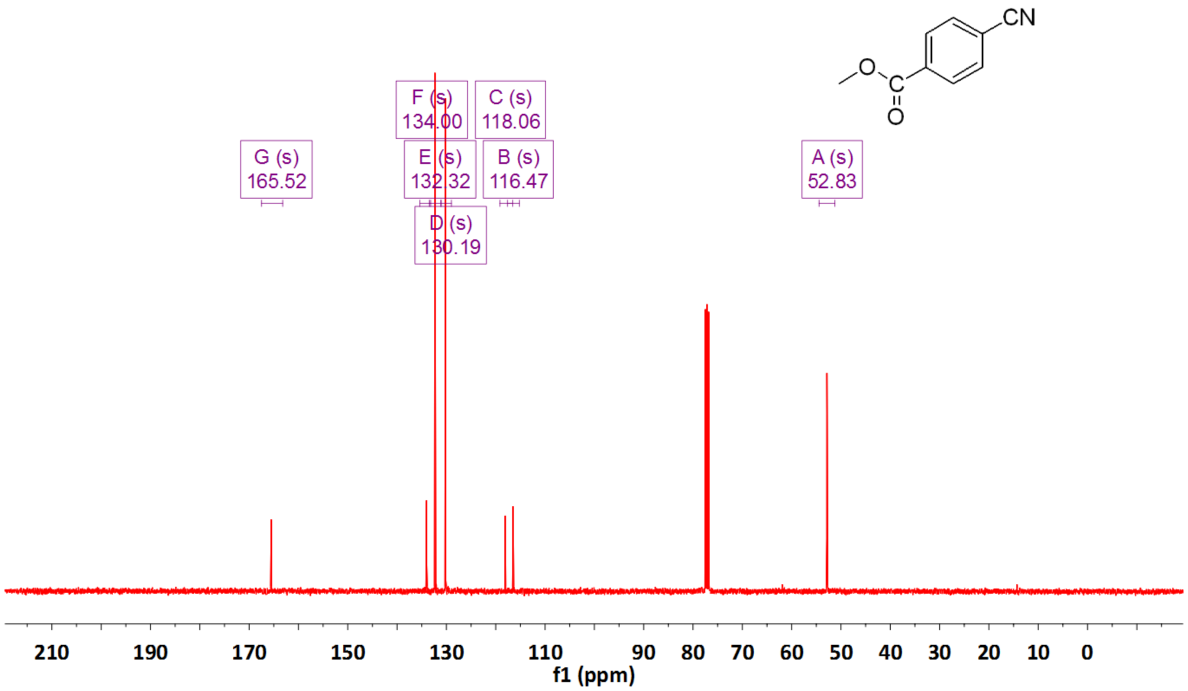

Compound **10h**

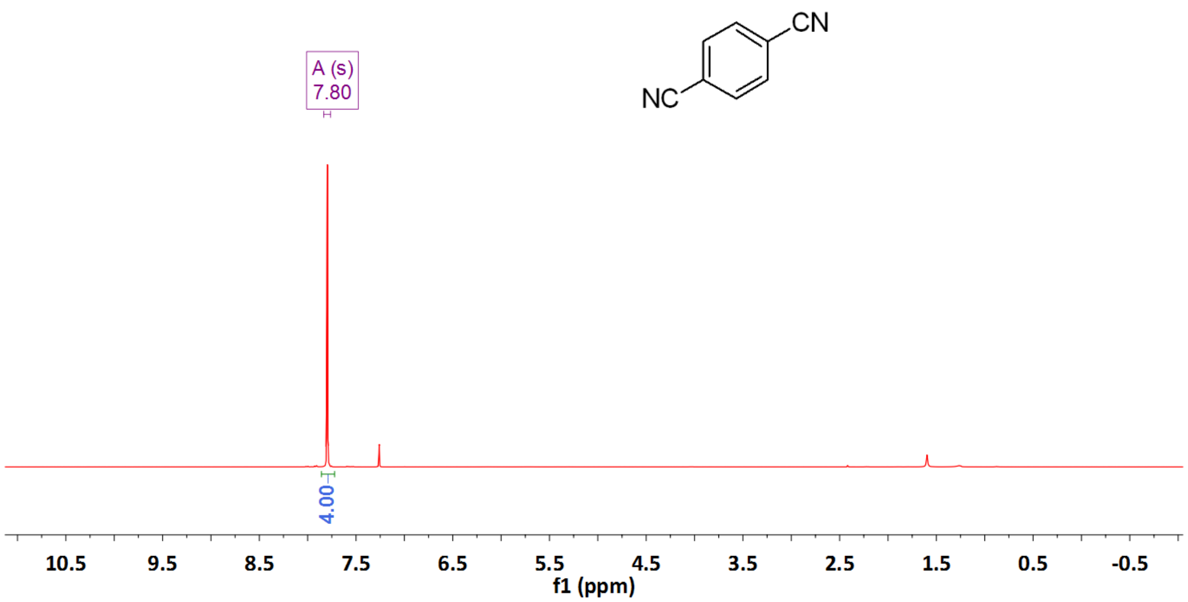

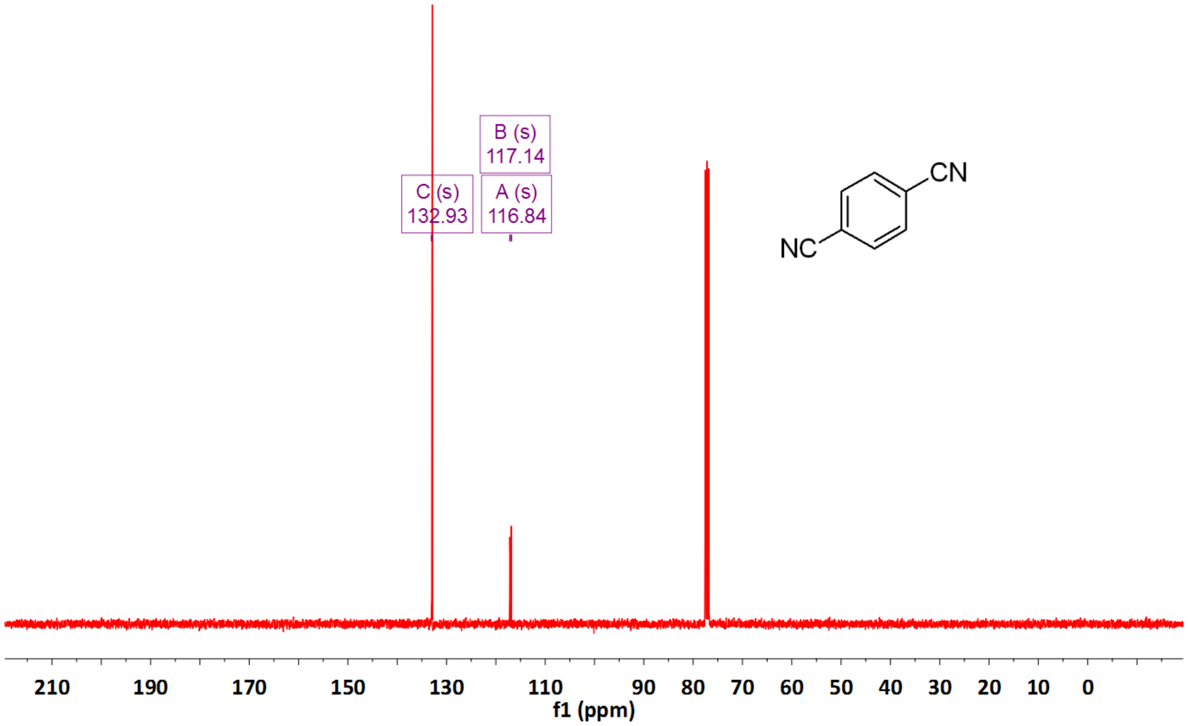

Compound **10i**

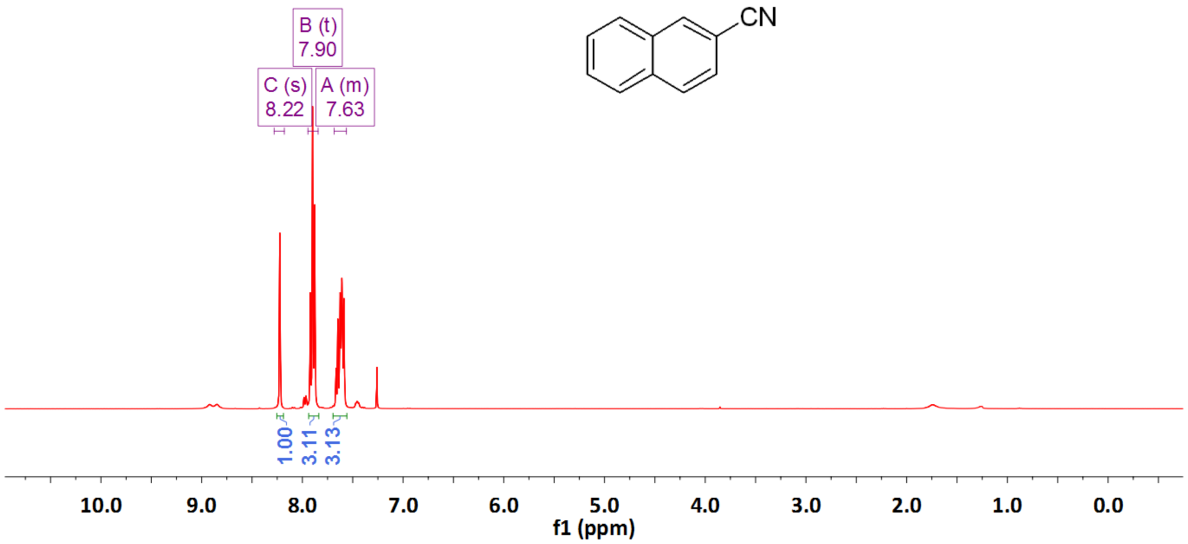

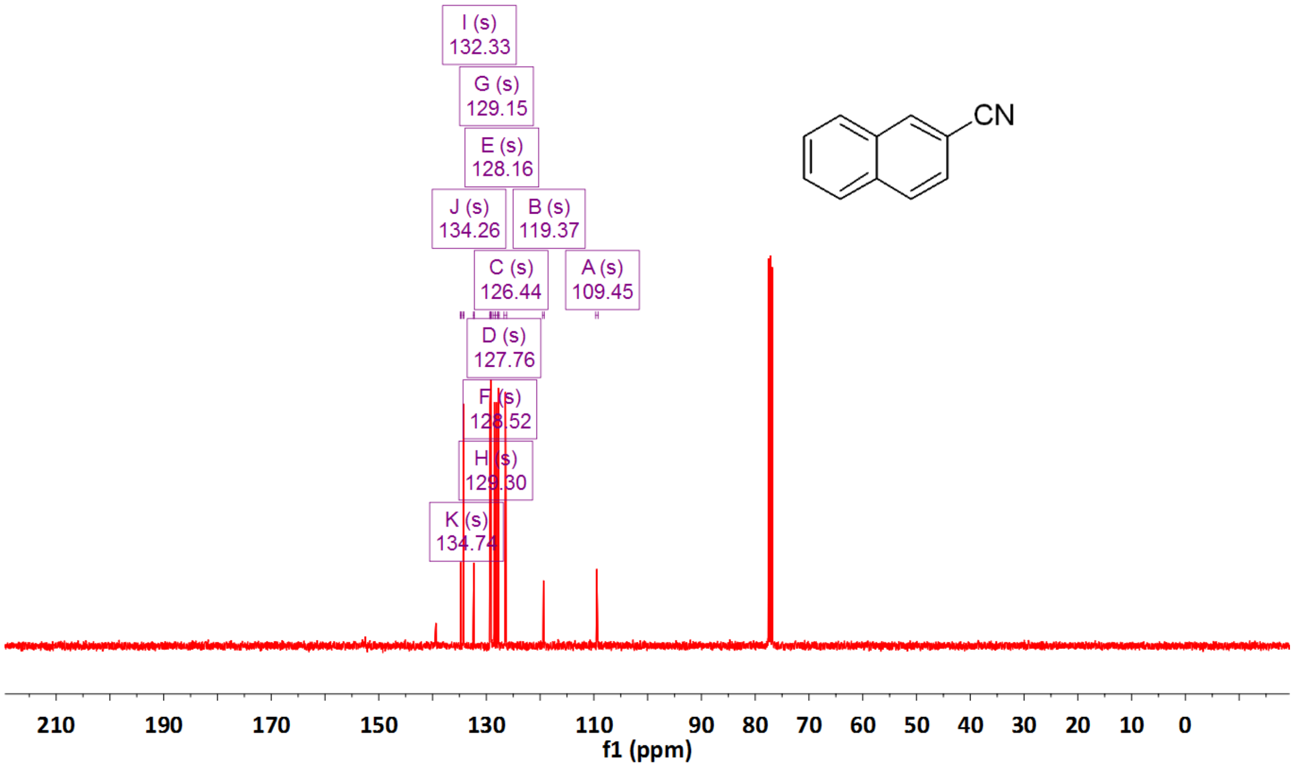

Compound **10j**

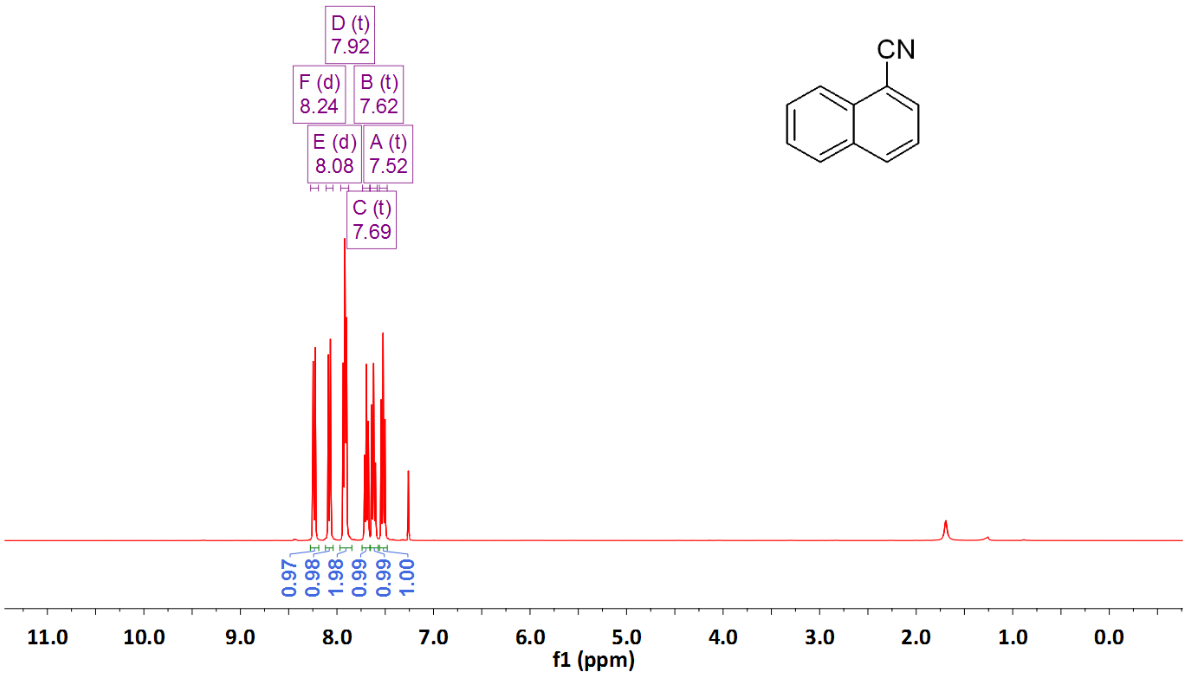

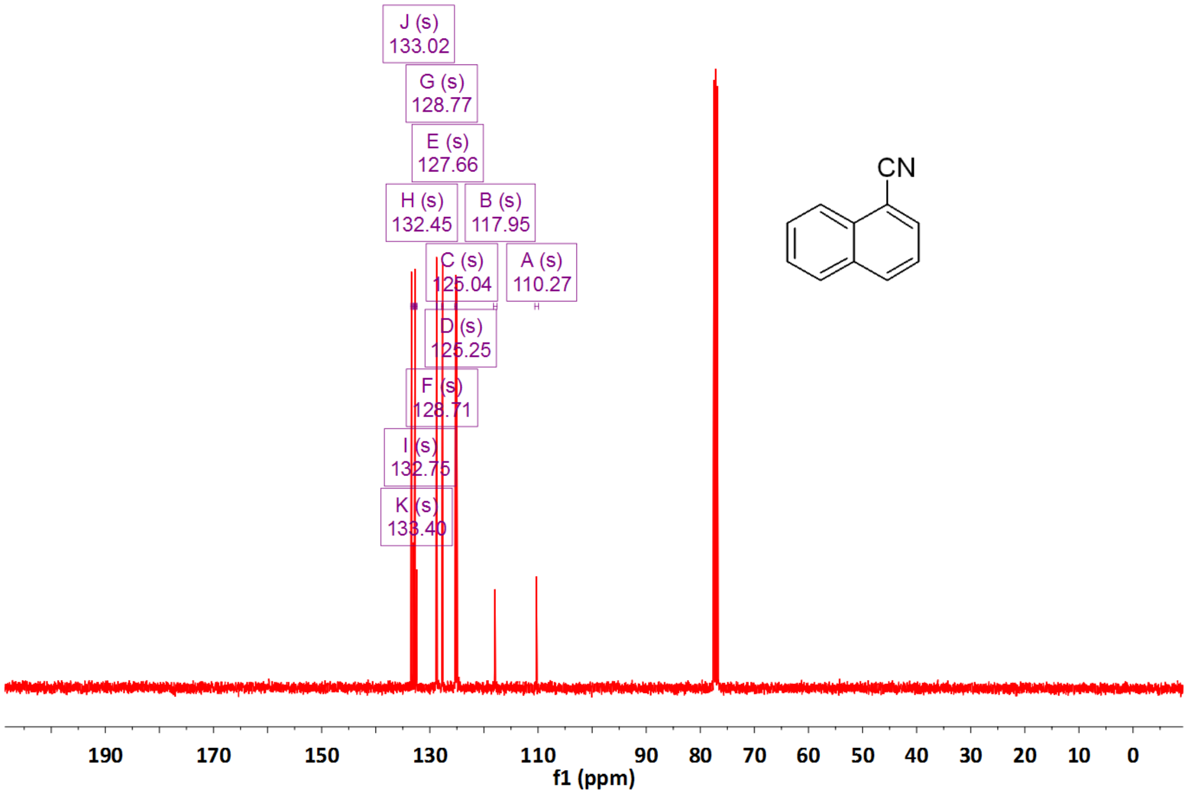

Compound **10k**

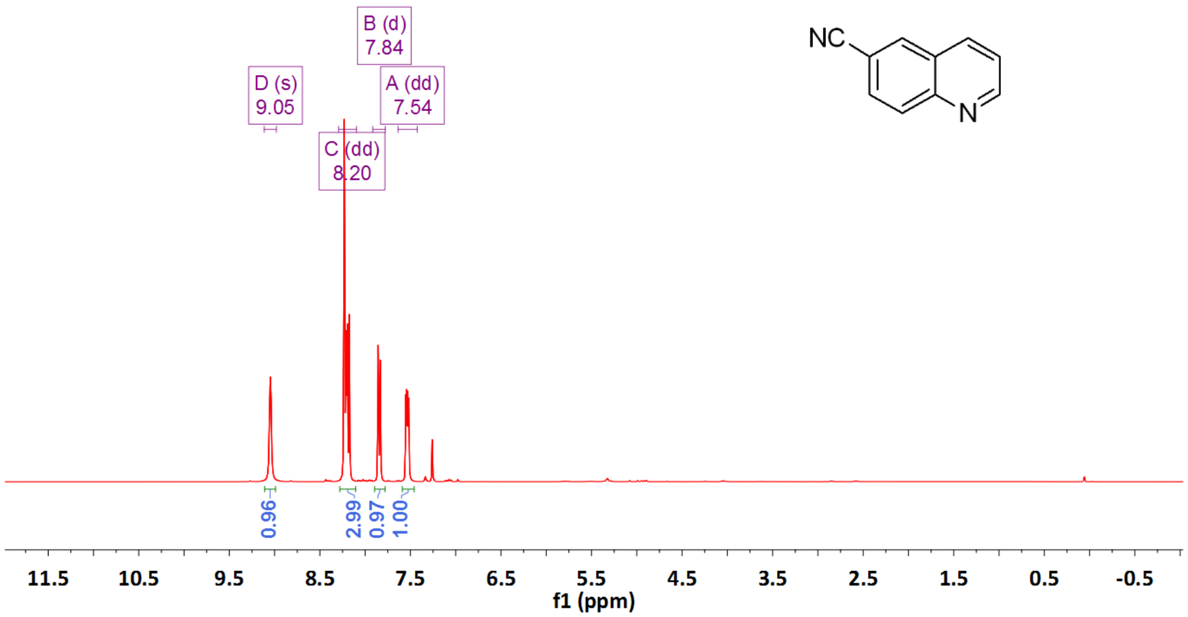

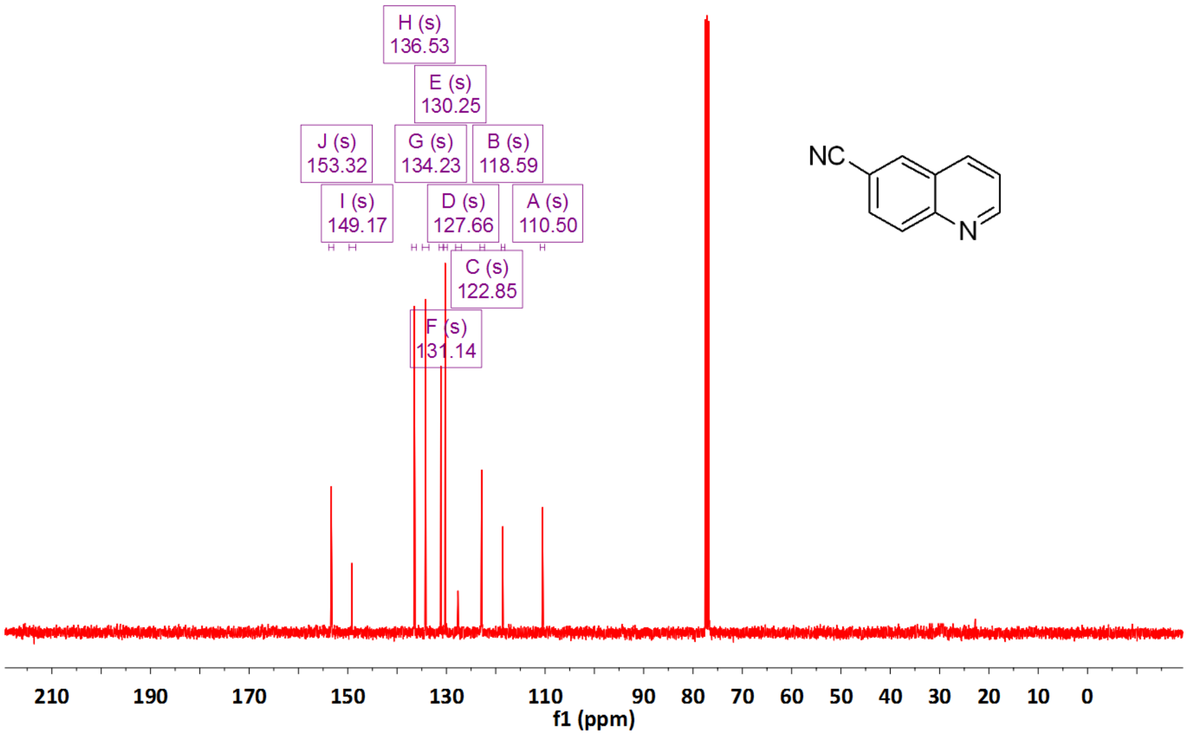

Compound **10l**

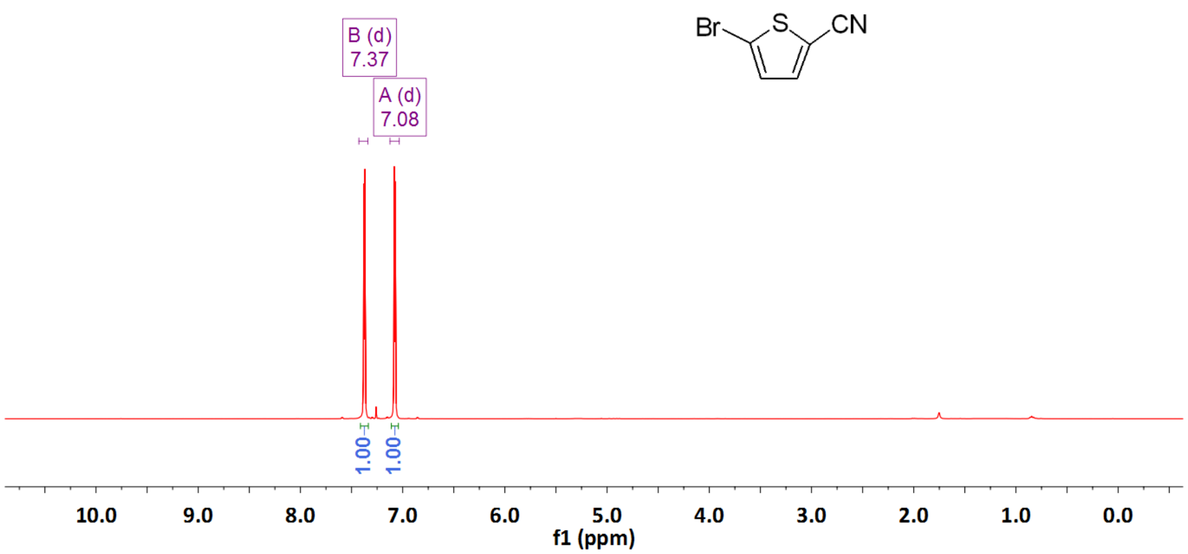

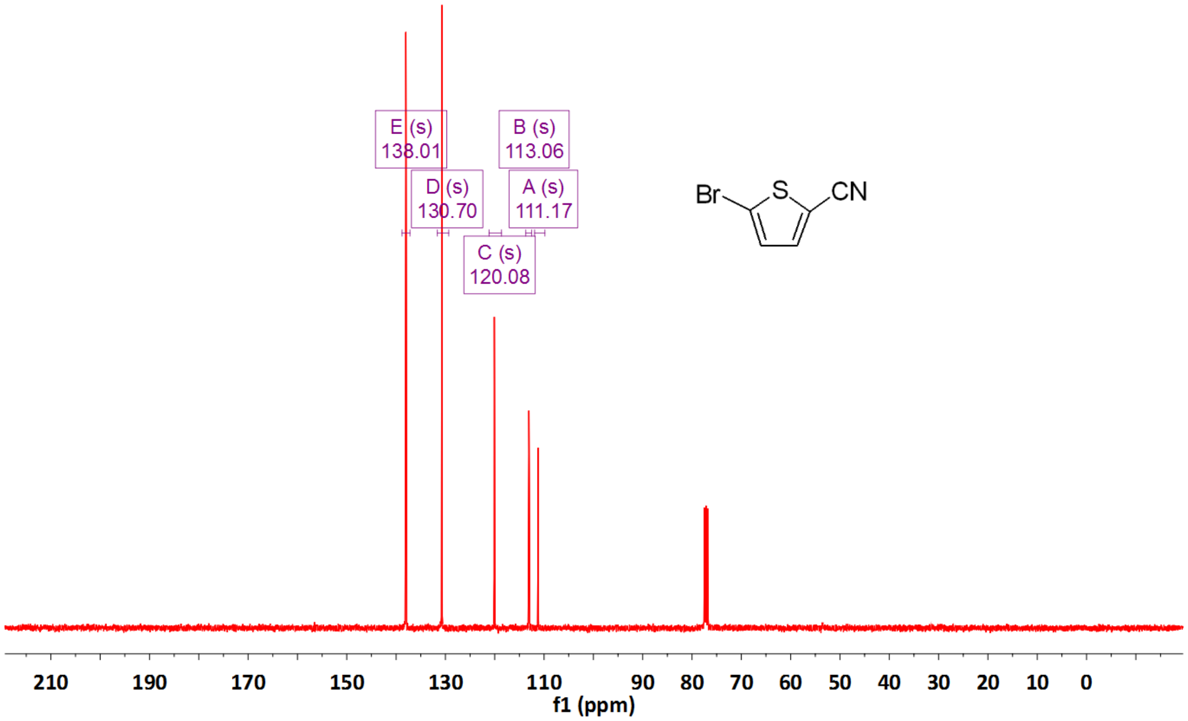

Compound **12**

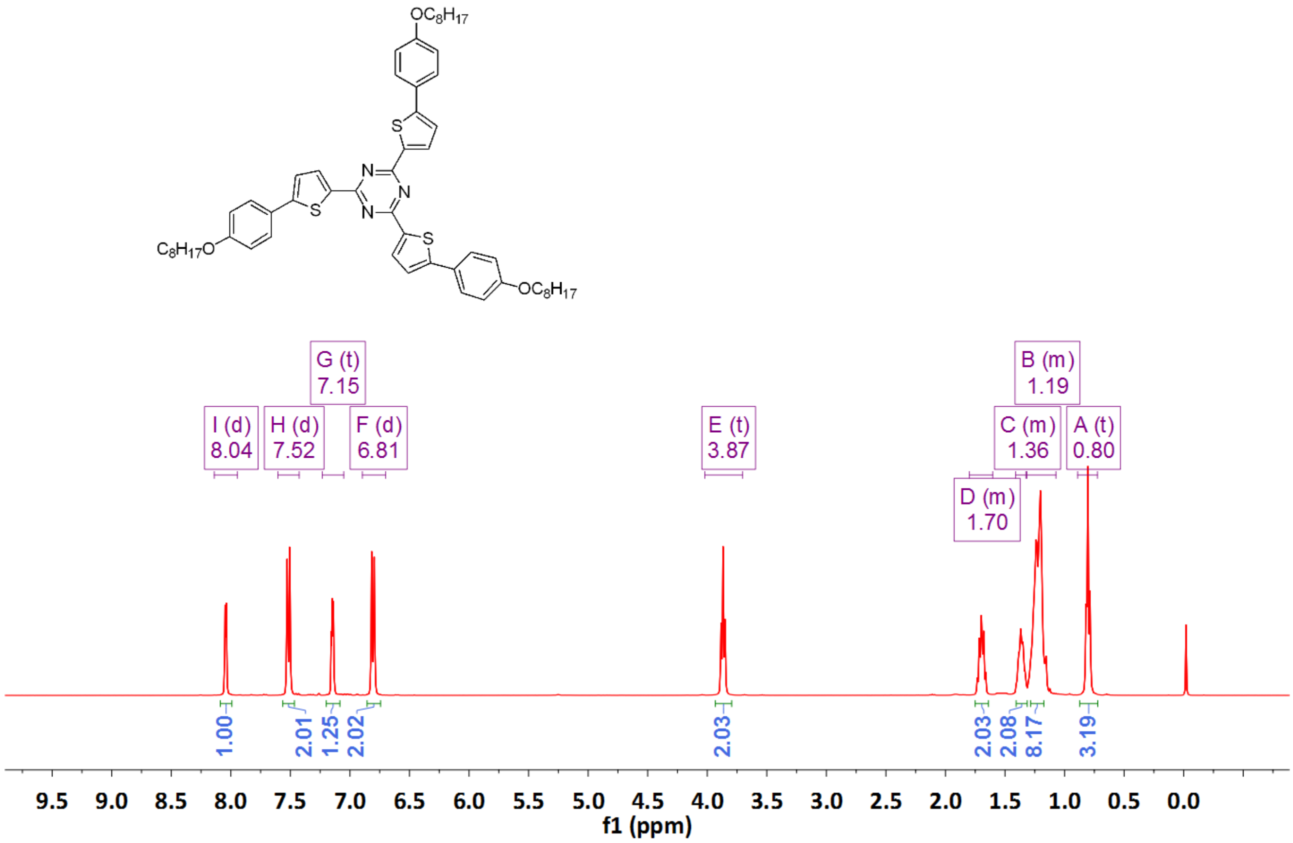

OC8H17
C8H17O
OC8H17
I (d) 8.04
H (d) 7.52
G (t) 7.15
F (d) 6.81
E (t) 3.87
D (m) 1.70
C (m) 1.36
B (m) 1.19
A (t) 0.80
1.00
2.01
1.25
2.02
2.03
2.03
2.08
8.17
3.19
9.5 9.0 8.5 8.0 7.5 7.0 6.5 6.0 5.5 5.0 4.5 4.0 3.5 3.0 2.5 2.0 1.5 1.0 0.5 0.0
f1 (ppm)

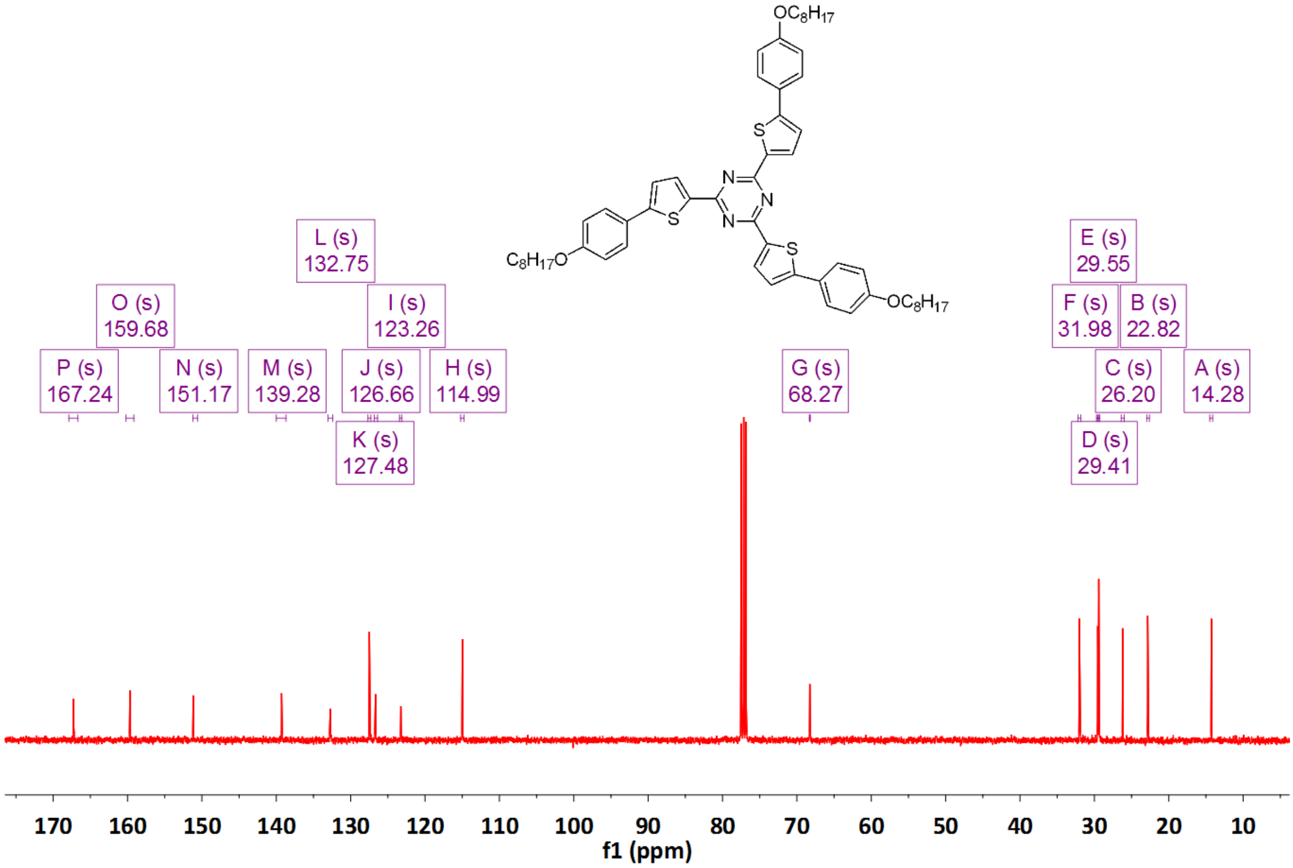

OC8H17
C8H17O
OC8H17
P (s) 167.24
O (s) 159.68
N (s) 151.17
M (s) 139.28
L (s) 132.75
K (s) 127.48
J (s) 126.66
I (s) 123.26
H (s) 114.99
G (s) 68.27
F (s) 31.98
E (s) 29.55
D (s) 29.41
C (s) 26.20
B (s) 22.82
A (s) 14.28
170 160 150 140 130 120 110 100 90 80 70 60 50 40 30 20 10
f1 (ppm)

9. References

1. Li, C.; Li, W.; Henwood, A.F.; Hall, D.; Cordes, D.B.; Slawin, A.M.Z.; Lemaur, V.; Olivier, Y.; Samuel, I.D.W.; Zysman-Colman, E. Luminescent Dinuclear Copper(I) Complexes Bearing an Imidazolylpyrimidine Bridging Ligand. *Inorg. Chem.* **2020**, *59*. 14772–14784. doi: 10.1021/acs.inorgchem.0c01866.
2. Yasuda, T.; Shimizu, T.; Liu, F.; Ungar, G.; Kato, T. Electro-functional octupolar π-conjugated columnar liquid crystals. *J. Am. Chem. Soc.* **2011**, *133*, 13437–13444. doi: 10.1021/ja2035255. Epub 2011 Aug 10. PMID: 21790175
3. Maeda, H., Eifuku, N. Alkoxy-substituted Derivatives of π-Conjugated Acyclic Anion Receptors: Effects of Substituted Positions. *Chem. Lett.* **2009**, *38*, 208–209. https://doi.org/10.1246/cl.2009.208
4. Percec, V.; Wang, S.; Huang, N.; Partridge, B.E.; Wang, X.; Sahoo, D.; Hoffman, D.J.; Malineni, J.; Peterca, M.; Jezorek, R.L.; et al. An Accelerated Modular-Orthogonal Ni-Catalyzed Methodology to Symmetric and Nonsymmetric Constitutional Isomeric AB2 to AB9 Dendrons Exhibiting Unprecedented Self-Organizing Principles. *J. Am. Chem. Soc.* **2021**, *143*, 17724–17743.
5. CrysAlis Pro; Oxford Diffraction: Yarnton, England, 2010.
6. Sheldrick, G.M. A short history of SHELX. *Acta Crystallogr.* A **2008**, *64*, 112–122.
7. Sheldrick, G.M. Crystal Structure Refinement with SHELXL. *Acta Crystallogr. C* **2015**, *C71*, 3–8.
8. Gagne, R.R.; Koval, C.A.; Lisensky, G.C. Ferrocene as an internal standard for electrochemical measurements. *Inorg. Chem.* **1980**, *19*, 2854–2855.
